# Caveolin-1 knockout improves CFA-induced inflammatory pain in adult mice through modulating the cGAS STING pathway and autophagy

**DOI:** 10.1371/journal.pone.0333646

**Published:** 2025-10-09

**Authors:** Huan Chang, Cancan Cheng, Ning Zheng, Haiyan Zhu, Hui Yang, Xiaocheng Zhu, Fan Zhang

**Affiliations:** 1 Department of Anesthesiology, The Third Xiangya Hospital, Central South University, Changsha, China; 2 Department of Anesthesiology, Xiangya Hospital, Central South University, Changsha, China; University of Hawai'i at Manoa College of Tropical Agriculture and Human Resources, UNITED STATES OF AMERICA

## Abstract

**Background:**

Inflammatory pain poses a significant clinical challenge, with its underlying mechanisms not yet fully elucidated. This study investigated the role of Caveolin-1 (Cav1) in inflammatory pain and elucidated its molecular mechanisms.

**Methods:**

We analyzed public databases and employed a mouse model of inflammatory pain induced by complete Freund’s adjuvant (CFA). *Cav1*-knockout *(Cav1*^*-/-*^) mice were used to evaluate Cav1’s function. The study incorporated behavioral tests, immunohistochemistry and molecular analyses. BV2 microglial cells served as the in vitro model.

**Results:**

Following CFA injection, Cav1 expression was markedly elevated in the dorsal horn of spinal cord, correlating with pain behavior and inflammatory responses. *Cav1*^*-/-*^ mice demonstrated significantly reduced pain behavior and inflammatory responses after CFA induction. Mechanistically, Cav1 enhanced inflammation by activating the cGAS-STING pathway and inhibiting autophagy. In BV2 microglia, Cav1 overexpression increased proinflammatory cytokine expression (TNF-α, IL-1β, IL-6) while inhibiting autophagy, whereas *Cav1* knockdown produced opposing effects.

**Conclusion:**

This study reveals a novel role of Cav1 in inflammatory pain, demonstrating its regulation of inflammation through modulation of the cGAS-STING pathway and autophagy. These findings advance our understanding of the pathogenesis of inflammatory pain and identify Cav1 as a potential therapeutic target.

## Introduction

Inflammation serves as the body’s natural defense against deleterious stimuli, with its primary objective being the facilitation of tissue repair. But persistent inflammatory states are closely associated with chronic pain [1], especially when the glial cells are activated in the central nervous system. This activation leads to neuroinflammation and central sensitization, which are critical mechanisms underlying the onset of chronic pain [2–5]. However, the causes of central inflammation and pain sensitization remain elusive.

Caveolins are a family of transmembrane proteins resident within caveolae, small lipid raft invaginations of the plasma membrane. The functions of these caveolin-rich lipid rafts are multifaceted, encompassing mechano-protection, lipid homeostasis, metabolism, transport, and cell signaling [6]. Caveolin-1 (Cav1) is an isoform of caveolins, and present in different cell types, including endothelial cells, neurons, astrocytes, oligodendrocytes, and microglia [7]. It involved in signal transduction, oxidative stress, autophagy, and apoptosis [8–10]. Recent studies have highlighted its critical role in neuroinflammation [11–16]. In resting microglia, Cav1 expression is relatively low, but significantly increases under inflammatory conditions [17]. Inhibiting Cav1 expression has been observed to alleviate neuroinflammation [18]. It suggests that Cav1 may play a pivotal role in the regulation of neuroinflammation. Additionally, the cGAS-STING signaling pathway, a key component of innate immunity, has gained significant attention for its pivotal role in inflammation [19]. Aberrant activation of this pathway not only exacerbates inflammatory responses but may also modulate the pain processes through its interplay with autophagy. Whether Cav1 regulates inflammatory pain via the cGAS-STING pathway and autophagy remains unexplored. This study utilized public database and a mouse model of inflammatory pain induced by complete Freund’s adjuvant (CFA), combined with *Cav1* gene knockout, behavioral tests, and molecular analyses, to systematically investigate the role of Cav1 in inflammatory pain and its underlying mechanisms.

## Methods

### Experimental design

**Fig 1:**
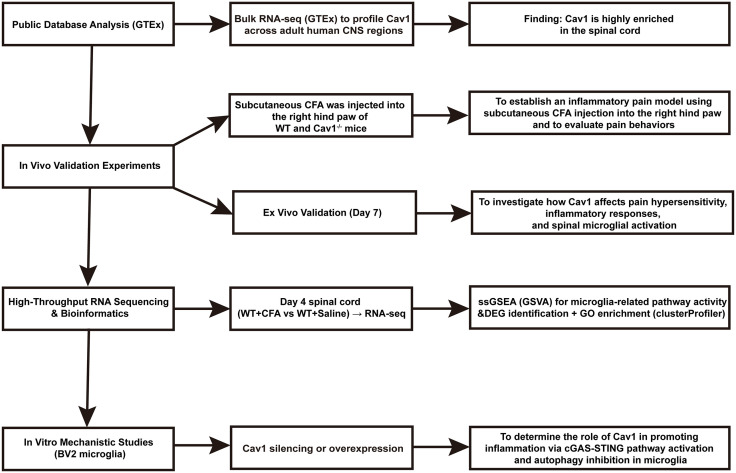
Schematic illustration of the experimental design. This study was composed of four major components. First, Cav1 expression in human central nervous system tissues was analyzed using bulk transcriptomic data from the GTEx database. Second, the inflammatory pain model was established via subcutaneous injection of complete Freund’s adjuvant (CFA) or saline into the right hind paw of adult mice, followed by behavioral testing from Day –3 (baseline) to Day 7. On Day 4, spinal cord tissues were collected for high-throughput RNA sequencing and subsequent differential gene expression (DEG) analysis, Gene Ontology (GO) enrichment, and single-sample gene set enrichment analysis (ssGSEA). On Day 7, molecular validation (qPCR, Western blot, immunofluorescence, HE staining) was performed ex vivo. Finally, in vitro validation was conducted using BV2 microglial cells through Cav1 silencing or overexpression, followed by Western blot and qPCR analysis.

This study aimed to investigate the role of Cav1 in inflammatory pain and its potential mechanisms using public transcriptomic datasets, high-throughput RNA sequencing, in vivo models, and in vitro experiments. The study was composed of four major components:

Public Database Analysis: Bulk RNA-sequencing data from the GTEx database were used to examine the expression profile of *Cav1* across human central nervous system tissues.
In Vivo Validation Experiments: A inflammatory pain model was established by subcutaneous injection of complete Freund’s adjuvant (CFA) into the right hind paw of adult mice. Mechanical and thermal sensitivity were assessed at baseline and on days 1, 3, 5, and 7 post-injection. On day 7, spinal cord samples were collected for qPCR, Western blot, immunofluorescence, and histopathological evaluation.
High-Throughput Sequencing and Bioinformatic Analysis: Spinal cord tissues were collected from CFA and Saline mice on day 4 after modeling for RNA extraction and high-throughput transcriptome sequencing. Differentially expressed genes (DEGs) between groups were identified, followed by Gene Ontology (GO) enrichment analysis using the clusterProfiler R package. Single-sample gene set enrichment analysis (ssGSEA) was performed using the GSVA R package to assess microglia-related pathway activities at the individual sample level.
In Vitro Mechanistic Studies: To further explore the molecular mechanisms underlying Cav1-mediated microglial activation, BV2 murine microglial cells were used for in vitro experiments. Cav1 expression was silenced or overexpressed by transfection, and downstream signaling pathways were analyzed by Western blotting and qPCR under inflammatory stimulation conditions. The workflow of the study is depicted in a schematic diagram (Fig 1).

### Animals

Male C57BL/6J mice (8–10 weeks old, 20–30 g) were obtained from the Experimental Animal Center of Central South University. *Cav1 ⁻ / ⁻ *mice were kindly provided by Dr. You-Yang Zhao (University of Illinois College of Medicine, Chicago, IL, USA). These mice were generated using homologous recombination by electroporating a neomycin resistance gene-containing targeting vector into WW6 embryonic stem (ES) cells (genetic background: 75% 129/Sv, 20% C57BL/6J, 5% SJL), resulting in the deletion of exons 1 and 2 of the Cav1 gene (a total of 2.2 kb). Correctly targeted ES cells were selected and injected into C57BL/6 blastocysts to produce chimeric mice. The resulting line was subsequently backcrossed onto a C57BL/6 genetic background for stable propagation. The homozygous Cav1 ⁻ / ⁻ mice used in this study exhibited normal fertility, with no obvious reproductive abnormalities observed. Genotyping primers and protocols were based on the recommendations of The Jackson Laboratory (https://www.jax.org/strain/007083), and representative genotyping results are provided in S1 Raw Images.

All animals were housed in a specific pathogen-free (SPF) facility with controlled environmental conditions (temperature 22 ± 2 °C, relative humidity 50–60%) under a 12 h light/dark cycle (lights on from 07:00–19:00), with free access to food and water. All experimental procedures were approved by the Animal Ethics Committee of Central South University (Approval No. CSU-2024–0001) and conducted in accordance with institutional guidelines and the ethical standards of the International Association for the Study of Pain (IASP) [20].

### Public database analysis

To explore the expression profile of Cav1 in central nervous system tissues, bulk RNA-seq data were retrieved from the Genotype-Tissue Expression (GTEx) database (https://www.gtexportal.org). Normalized transcript per million (TPM) values for Cav1 across various adult human CNS regions were extracted using the GTEx portal’s built-in data visualization tools. Graphical analyses were performed using both the GTEx interface and GraphPad Prism software (version 7.0). Only samples from adult donors were included in the analysis.

### Induction of inflammatory pain

The inflammatory pain model was established using complete Freund’s adjuvant (CFA) (Sigma-Aldrich, St. Louis, MO, USA). Mice were randomly assigned to experimental group (*n* = 15) and control group (*n* = 15). The experimental group received a subcutaneous injection of CFA (20 μL, 1 mg/mL) into the right hindpaw, while the control group received an equal volume of sterile saline (0.9% NaCl).

For injection, mice were manually restrained to expose the plantar surface of the right foot. Using aseptic technique, a needle was inserted at 15–20° angle beneath the plantar skin, and the CFA solution (20uL) was administered slowly. Post-injection, a gentle pressure was applied to the injection site for 3–5 seconds to prevent solution reflux. Subsequently, mice were housed individually and monitored for behavioral changes and general health status.

### Temporal grouping for *Cav1* expression analysis

To assess the temporal dynamics of *Cav1* expression in the spinal cord dorsal horn during CFA-induced inflammatory hyperalgesia, separate groups of mice were sacrificed at five distinct timepoints: baseline (Day –1), and Days 1, 3, 5, and 7 following CFA or saline injection. At each timepoint, lumbar spinal cord segments (L4–L6) were rapidly dissected for molecular analysis. For qPCR analysis, six mice (*n* = 6) were used per timepoint, while five mice (n = 5) per group were used for Western blotting. Each animal was used for tissue collection at a single timepoint only, and no animals were reused. This design allowed for accurate evaluation of dynamic changes in Cav1 expression throughout the progression of inflammatory hyperalgesia.

### High-throughput sequencing

Four days post-CFA induction, spinal cord tissue was collected from wild-type (WT) and *Cav 1* knockout (Cav1^-/-^) mice (*n* = 3 per group). Total RNA was extracted from the ipsilateral lumbar dorsal horn (L4-L6) using the RNeasy Micro Extraction kit (Qiagen, Hilden, Germany). RNA quality was assessed using a NanoDrop ND-1000 spectrophotometer (Thermo Fisher Scientific, USA; A260/A280 ratio 1.8-2.1) and Agilent 2100 Bioanalyzer (Agilent Technologies, USA; RNA integrity number >8). Samples were further purified using the RNAClean XP kit (Beckman Coulter, USA) and treated with RNase-free DNase (Qiagen) to eliminate contamination. Library construction was performed using the TruSeq® Stranded Total RNA Sample Preparation kit (Illumina, USA). Library quality was verified using a Qubit 4.0 fluorometer (Invitrogen, USA) for concentration and an Agilent 2100 Bioanalyzer for fragment size distribution (200–300 bp insert size). Sequencing was performed on the Illumina NovaSeq 6000 platform using paired-end 150 bp reads, generating minimum 6 Gb of raw data per sample. These data were used for subsequent bioinformatic analysis to investigate the differences in gene expression between the WT-CFA group and the *Cav1*^*-/-*^-CFA group.

### Single-Sample gene set enrichment analysis (ssGSEA)

Single-sample gene set enrichment analysis (ssGSEA), an extension of the GSEA method, was performed using the GSVA R package to evaluate the activity of biological pathways at the individual sample level. Normalized gene expression matrices were used as input, and enrichment scores were calculated for each sample.

Gene sets associated with microglial activation and proliferation were curated from the Gene Ontology (GO) database. The resulting enrichment scores were visualized as a heatmap using the pheatmap R package, with row-wise z-score normalization applied to enable cross-sample comparison. When applicable, differences in ssGSEA scores between groups were assessed using the Wilcoxon rank-sum test.

### GO enrichment analysis

Differentially expressed genes (DEGs) were selected from high-throughput sequencing data and analyzed with the enrichGO function of clusterProfiler package. The enrichment results were corrected for multiple testing using the Benjamini-Hochberg (BH) method. Visualization was done using the GOplot package in the R language.

### Mechanical and thermal hyperalgesia assessment

All mice were acclimated to the testing environment for 30 minutes prior to behavioral assessments. Baseline paw withdrawal threshold (PWT) and thermal withdrawal latency (TWL) were measured on three consecutive days before CFA injection, and the values recorded on Day –1 were used as baseline reference. Behavioral evaluations were performed on Days 1, 3, 5, and 7 after CFA injection to assess the development of hyperalgesia.

Mechanical hyperalgesia was assessed using the von Frey up-and-down method. Following a 30-minute acclimation, the plantar surface of the right hind paw was stimulated perpendicularly with a series of calibrated von Frey filaments (Stoelting, Wood Dale, IL). Each filament was applied until it just bent and held in place for 5 seconds. A brisk paw withdrawal or twitching was recorded as a positive response. Each mouse was tested twice, and the average was taken as the mechanical threshold. To ensure reliability, tests were conducted 10 minutes apart under identical environmental conditions.

Thermal hyperalgesia was evaluated using a hot plate test maintained at 53 °C. Mice were placed on the heated surface, and the latency to hind paw withdrawal or licking was recorded as the response time. A 20-second cutoff was imposed to prevent tissue damage. Each mouse underwent three trials with 10-minute intervals, and the average latency was used for analysis. The hot plate was calibrated before each test session, and mice were continuously observed during testing to ensure safety and data consistency.

### Paw edema measurement and relative paw area calculation

Paw edema was assessed by measuring the thickness of the right hind paw using an electronic digital vernier caliper (SATA, China). The caliper was placed at the center of the paw and gently closed, with measurements read to the nearest 0.1 mm. Each paw was measured three times at 30-second intervals, and the average value was calculated. Calipers were calibrated before and after each measurement to ensure accuracy, and body temperature was maintained to avoid cold-induced limb contraction that might interfere with the measurements.

Paw inflammation was further evaluated by calculating the relative paw area ratio based on standardized digital images taken at a fixed height and angle. The surface areas of the right (injected) and left (contralateral) hind paws were outlined and measured using ImageJ software (National Institutes of Health, USA). The relative area was expressed as the ratio of the right paw area to the left paw area (right/left) for each mouse. This internal normalization minimized inter-individual variability. Each measurement was performed in triplicate, and the average value was used for analysis.

### Histopathological assessment

Prior to hematoxylin-eosin (HE) staining, the mice were first deeply anesthetized with sevoflurane and then subjected to cardiac perfusion. The perfusion process involves removing blood using 0.1M phosphate-buffered saline (PBS, pH 7.4) at 4°C, followed by fixation with 4% paraformaldehyde (PFA). After perfusion, the right hind paw tissue of the mouse was immediately removed and fixed in 4% PFA for 48 hours. The fixed tissue was then processed using standard paraffin embedding histological methods. The paraffin-embedded tissue blocks were sectioned to a thickness of 5 μm. The sections were then sequentially dewaxed and rehydrated: first, they are placed in xylene twice for 5 minutes each, then immersed in ethanol solutions (100%, 90%, 80%, 70%) for 3 minutes each, and finally rinsed with distilled water.

For staining, the sections were immersed in hematoxylin dye solution for 5 minutes, then rinsed with running water for 10 minutes. The sections were briefly dipped in 1% hydrochloric acid (HCl) solution, followed by a 2-minute rinse with running water. The sections were then stained in 0.5% eosin dye solution for 2 minutes, followed by rinsing under running water for 30 seconds to 1 minute, until the sections displayed the desired red coloration. After staining, the sections were sequentially dehydrated in 70%, 80%, 95%, 100%, and 100% ethanol, and then treated twice with xylene for 5 minutes each. The slides were sealed with neutral gum and covered with cover glass, then left to dry at room temperature. After completing the HE staining procedure, the tissue morphology and pathological changes were observed and photographed using a light microscope. Histological images were acquired at 15 × magnification with a 100 μm scale bar shown for reference.

Histopathological assessment was performed based on established criteria. Inflammatory responses were graded on a 0–3 scale: 0 indicated absence of inflammatory cells; 1 represented sparse, scattered inflammatory cells; 2 denoted organized inflammatory cell clusters surrounding blood vessels; and 3 corresponded to marked perivascular cell accumulation extending into adjacent parenchymal regions [21].

### Transmission electron microscopy (TEM)

Spinal cord tissue samples were excised into 1 mm³ blocks within 1–3 minutes and immediately immersed in electron microscopy fixative. After sectioning and transferring to EP tubes with fresh fixative at 4°C, the samples were washed three times in 0.1 M phosphate buffer (pH 7.4) for 10 minutes. Post-fixation was done in 1% osmium tetroxide for 2 hours at room temperature, followed by three 15-minute washes. The samples were dehydrated in graded ethanol (30%, 50%, 70%, 80%, 95%, and two 100% rounds) for 20 minutes each, then washed twice in acetone for 15 min. Infiltration was done in a 1:1 mixture of acetone and 812 resin for 2–4 hours at 37°C, then overnight in a 1:2 mixture at 37°C, and pure 812 resin for 5–8 hours at 37°C. The samples were embedded in resin, polymerized overnight at 37°C and polymerized for 48 hours at 60°C. Ultrathin sections (~80 nm) were cut using an ultramicrotome, placed on 200-mesh copper grids, and stained with 2% uranyl acetate for 8 minutes, washed, counterstained with 2.6% lead citrate for 8 minutes, and then air-dried overnight. Grids were analyzed under a transmission electron microscope.

### Immunofluorescence staining

Prior to immunofluorescence staining, mice were deeply anesthetized with sevoflurane and cardiac perfused. The perfusion procedure involves irrigation with 4°C 0.1M phosphate-buffered saline (PBS, pH 7.4) to remove blood, followed by fixation with 4% paraformaldehyde (PFA). Immediately after perfusion, spinal cord tissue was harvested and fixed in 4% PFA for 24 hours. The fixed tissue was then placed sequentially in 15% and 30% sucrose in 0.01 M PBS for dehydration at 4°C until it was precipitated.

The fixed and dehydrated spinal cord tissue was sectioned at a thickness of 20 μm using a cryostat and mounted directly on polylysine-coated slides, stored at −20°C. For immunofluorescence staining, sections were first washed three times in PBS for 5 minutes each, followed by permeabilization in PBS containing 0.3% Triton X-100 to enhance cell membrane permeability. Next, sections were blocked with 5% normal goat serum (NGS) for 1 hour to minimize non-specific binding.

After blocking, the sections were incubated overnight with primary antibodies (rabbit anti-GFAP, 1:500; mouse anti-Caveolin-1, 1:200; both from Santa Cruz Biotechnology, USA) at 4°C. The following day, the sections were washed three times with PBS for 5 minutes each, then incubated at room temperature with the corresponding fluorescent secondary antibodies (Alexa Fluor 488-labeled goat anti-rabbit IgG, 1:1000; Alexa Fluor 594-labeled goat anti-mouse IgG, 1:1000) for 1 hour After secondary antibody incubation, the sections were washed three times with PBS for 5 minutes each and then stained with DAPI (1:1000) to label the nuclei. Finally, the slides were mounted with an anti-fade mounting medium.

Immunofluorescence images were acquired using an Olympus BX53 fluorescence microscope. Z-stack images were captured with a 20x oil immersion objective (step size of 0.5 μm). Image analysis was performed using ImageJ software to quantify fluorescence intensity and assess the cellular localization of specific proteins.

### Cell culture

In this study, BV-2 microglial cells, from Wuhan Pricella Biotechnology Co., Ltd., were employed. These cells were cultured in high-glucose Dulbecco’s Modified Eagle Medium (DMEM; Invitrogen, Carlsbad, CA, USA), supplemented with 5% fetal bovine serum (FBS; Invitrogen, Carlsbad, CA, USA) to provide essential nutrients. To ensure optimal growth and proliferation, the cells were incubated at 37°C in an environment with 5% CO₂ and saturated humidity.

### Cell transfection

To investigate the function of Cav1 gene in BV2 microglia, we synthesized four specific siRNA sequences (si-Cav1–1, si-Cav1–2, si-Cav1–3, si-Cav1–4) and a negative control (si-NC). BV2 cells in the log phase were seeded at 1 × 10⁵ cells per well in 24-well plates. After 24 hours, siRNA transfection was performed when cell confluence reached 50–60%, The siRNA (final concentration of 50 nM) and Lipofectamine RNAiMAX (1 µL) were diluted in 50 µL of serum-free Opti-MEM and incubated for 5 minutes at room temperature. The complexes were mixed and incubated for 20 minutes to form siRNA-liposome complex. The culture medium was replaced with fresh complete medium, and the complexes were added dropwise. After 48 hours, cells were harvested, and total RNA and protein were extracted for subsequent analysis.

To overexpress Cav1, we constructed a pcDNA3.1-Cav1 plasmid by cloning Cav1 cDNA into the pcDNA3.1(+) vector. The plasmids were verified by restriction enzyme digestion and sequencing. BV2 cells were seeded in 1 × 10⁵ cells at per well in 24-well plates. After 24 hours, when cells confluence reached 60–70%, the plasmid transfection was performed. One µg of plasmid or equal amount of empty vector and 2 µL lipofectamine 2000 were diluted in 50 µL Opti-MEM and incubated for 5 minutes. The complexes were mixed and incubated for 20 minutes. The culture medium was replaced, and the complexes were added dropwise. After 48 hours, cells were harvested, and total RNA and protein were extracted for subsequent analysis.

### Quantitative real-time reverse-transcription PCR (RT-qPCR)

To assess the expression levels of Cav 1 and other target genes, total RNA was extracted from L4-6 spinal cord tissues and BV-2 cells using TransZol Up reagent (Transgen Biotech, China). The RNA was reverse transcribed using an all-in-one™ quantitative PCR mix kit (GeneCopeia, China). The RT-qPCR was conducted using the HIEF® qPCR SYBR Green Master Mix (Low Rox Plus) kit (Yesen, China) according to the manufacturer’s instructions. Each sample was repeated three times, and the results were verified by independent researchers. Relative gene expression levels were calculated using the 2^(-ΔΔCT) method, with GAPDH as the reference gene. Primer sequences are listed in Table 1.

**Table 1:** List of qPCR primer sequences.

Gene	Gene ID	Forward primer 5′‐3′	Reverse primer 5′‐3′
*GAPDH*	14433	AGGTCGGTGTGAACGGATTTG	TGATGGGCTTCCCGTTGATG
*Caveolin-1*	12389	CAACTGAATGAGGCCAGCGT	GGGCTGGCTTAGAGTCAGGA
*TNF-α*	21926	CGCTCTTCTGTCTACTGAACTTCGG	GTGGTTTGTGAGTGTGAGGGTCTG
*IL-1β*	16176	CACTACAGGCTCCGAGATGAACAAC	TGTCGTTGCTTGGTTCTCCTTGTAC
*IL-6*	16193	CTTCTTGGGACTGATGCTGGTGAC	TCTGTTGGGAGTGGTATCCTCTGTG
*Aif1*	11629	ATCAACAAGCAATTCCTCGATGA	CAGCATTCGCTTCAAGGACATA
*Csf1r*	12978	TGTCATCGAGCCTAGTGGC	CGGGAGATTCAGGGTCCAAG

### Western Blot (WB)

Protein was extracted from ipsilateral lumbar (L4-L6) spinal cord tissue and BV2 microglia using RIPA lysis buffer with protease and phosphatase inhibitors. Homogenization was performed with magnetic beads, followed by centrifugation at 12,000 g for 30 minutes at 4°C to collect supernatants. Protein concentration was measured using the BCA Protein Assay kit (Cowin Bio, China). WB experiments were performed with reference to previous studies [22]. Specifically, 30 μg of protein per sample was separated by SDS-PAGE on a 12% gel and transferred to a PVDF membrane. Membranes were blocked with 5% skimmed milk in TBST and incubated overnight at 4°C with primary antibodies against Cav1 (1:3000, rabbit, Novus Biologics, USA), cGAS (1:1000, rabbit, Wuhan Sanying Biotechnology, China), STING (1:1000, rabbit, Wuhan Sanying Biotechnology, China), LC3 (1:1000, rabbit, Beyotime, China), p62 (1:1000, mouse, Beyotime, China), and GAPDH (1:5000, mouse, Beyotime, China). After washing, the membranes were incubated with HRP-conjugated secondary antibodies. Protein bands were visualized using ECL substrate, and the signals were captured using an imaging system. Quantitative analysis was conducted with ImageJ software, normalizing protein expression to GAPDH.

### Statistical analysis

Statistical analysis was conducted using GraphPad Prism Version 7.0. Behavioral data were analyzed by two-way ANOVA with repeated measures, followed by Bonferroni’s post-test to assess interactions and group differences over time. For RT-qPCR, Western blot, and immunofluorescence data, one-way ANOVA followed by Tukey’s post-test was used to compare experimental groups. A *p*-value of less than 0.05 was considered statistically significant. Data are presented as mean ± standard error of the mean (SEM).

## Results

### Cav1 expression in the dorsal horn of spinal cord was significantly upregulated during CFA-induced inflammatory pain

**Fig 2:**
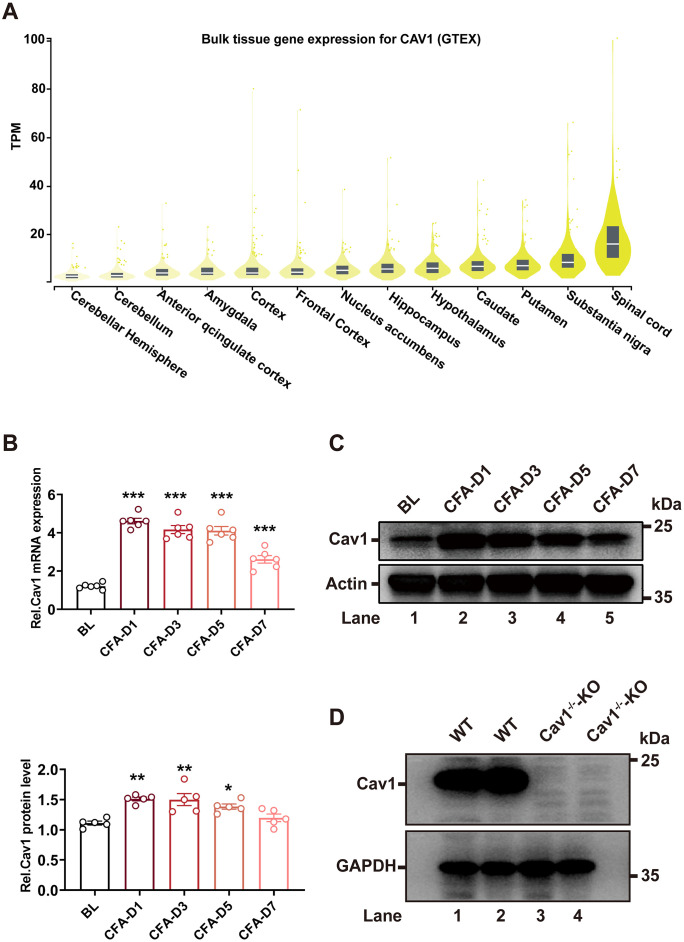
Upregulation of Cav1 in the spinal cord dorsal horn during CFA-induced inflammatory pain. **(A)** Analysis of public databases (GTEx) showing high expression of Cav1 in spinal cord tissue. **(B)** RT-qPCR analysis of *Cav1* mRNA expression in the spinal cord dorsal horn at different time points after CFA injection. Data are presented as mean ± SEM. n = 6 mice per group. One-way ANOVA followed by Tukey’s post hoc test was performed. **(C)** Western blot analysis of Cav1 protein levels in the spinal cord dorsal horn at different time points after CFA injection. Relative protein levels were quantified. Data are presented as mean ± SEM. n = 5 mice per group. One-way ANOVA followed by Tukey’s post hoc test was performed. **(D)** Western blot analysis of Cav1 protein expression in wild-type (WT) and *Cav1*^⁻*/*⁻^ mice. *P < 0.05; **P < 0.01; ***P < 0.001.

First, we explored the expression profile of Cav1 across adult human CNS tissues using public databases. This analysis showed that Cav1 expression is highly enriched in the spinal cord (Fig 2A). Next, to examine changes associated with inflammatory pain, we assessed Cav1 expression in the dorsal horn of the spinal cord using a CFA mouse model. The RT-qPCR analysis showed a significant increase in *Cav1* mRNA levels in the dorsal horn on day 1 after CFA injection, which persisted through day 7 (Fig 2B, S2 File). Western blot analysis confirmed a corresponding increase in Cav1 protein levels during the same period (Fig 2C, S1 Raw Images and S2 File). To further validate its role, we used Cav1 knockout (Cav1 ⁻ /⁻) mice, in which no Cav1 protein expression was detected, while the wild-type (WT) group showed obvious expression (Fig 2D, S1 Raw Images).

### Cav1 knockout alleviated pain behaviors and inflammatory responses in CFA-induced mice

**Fig 3:**
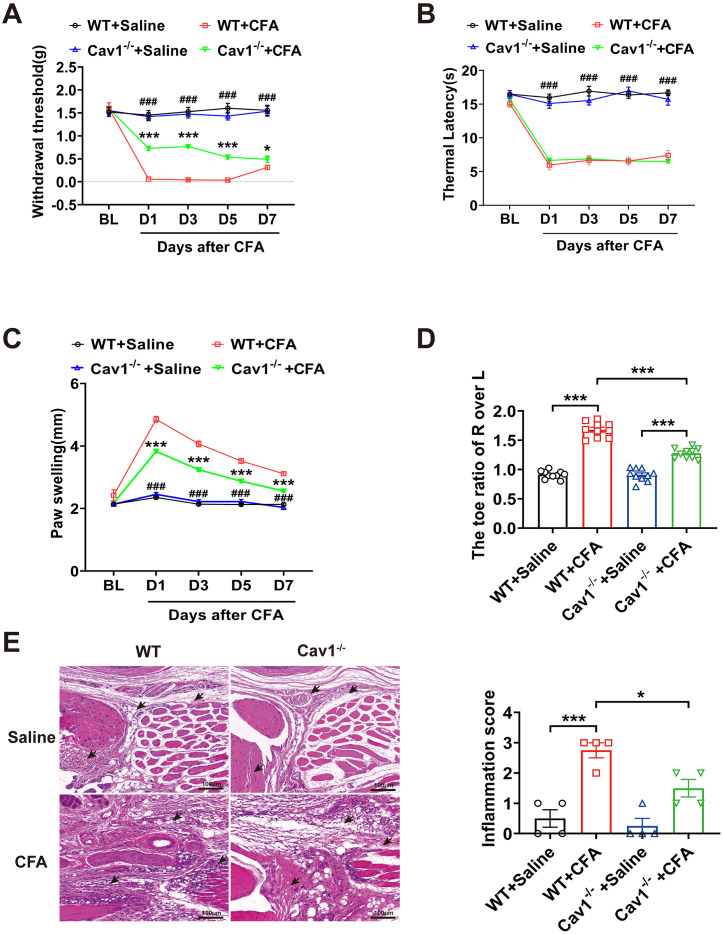
*Cav1* knockout attenuated CFA-induced pain behaviors and inflammatory responses. **(A)** Paw withdrawal threshold (PWT) in wild-type (WT) and *Cav1*^*-/-*^ mice injected with CFA or saline. **(B)** Thermal withdrawal latency in WT and *Cav1*^*-/-*^ mice injected with CFA or saline. **(C)** Right hind paw edema measured by caliper in WT and *Cav1*^*-/-*^ mice following CFA or saline injection. Data are presented as mean ± SEM. *n* = 15 mice per group. Statistical analysis for (A–C) was performed using two-way repeated measures ANOVA followed by Bonferroni post hoc test. “#” denotes comparisons between WT+Saline and WT + CFA. ^#^*P* < 0.05; ^##^*P* < 0.01; ^###^*P* < 0.001. “*” denotes comparisons between *Cav1*^*-/-*^ + CFA and WT + CFA. *P < 0.05; **P < 0.01; ***P < 0.001. **(D)** Relative right/left paw area in wild-type (WT) and *Cav1*^*-/-*^ mice after CFA or saline injection. Data are presented as mean ± SEM. *n* = 10 mice per group. **(E)** Representative H&E staining of right foot tissue from WT and *Cav1*^*-/-*^ mice after CFA or saline injection (15 × magnification, scale bar = 100 μm). Bar graph shows inflammation scores for each group. Data are presented as mean ± SEM. *n* = 4 mice per group. One-way ANOVA followed by Tukey’s post hoc test was performed for(D-E). *P < 0.05; ***P < 0.001.

To explore the role of Cav1 in inflammatory pain, we assessed mechanical and thermal sensitivity, as well as inflammatory parameters in WT and *Cav1*^*-/-*^ mice after CFA injection. Compared with WT+Saline mice, CFA injection significantly reduced paw withdrawal threshold (PWT) and thermal withdrawal latency (TWL) in WT mice from day 1 to day 7 (Fig 3A and 3B, S2 File). *Cav1*^*-/-*^ mice showed significantly higher PWT values compared to WT mice across the same time points (Fig 3A). No significant difference in TWL was observed between *Cav1*^*-/-*^ and WT mice at any time point following CFA injection (Fig 3B). Caliper measurements revealed significant swelling in the right hind paw of WT + CFA mice compared to WT+Saline mice, whereas paw edema was markedly reduced in Cav1^-/-^ + CFA mice compared to WT + CFA mice (Fig 3C, S2 File). Consistent with these findings, relative paw area analysis also showed significantly less edema in Cav1^-/-^ + CFA mice compared to WT + CFA mice (Fig 3D, S2 File).

Histological analysis further corroborated these findings. HE staining of paw tissue from WT + CFA mice revealed extensive infiltration of mononuclear inflammatory cells (mainly lymphocytes and monocytes), accompanied by tissue swelling and structural disruption. In contrast, *Cav1*^*-/-*^ mice exhibited relatively preserved tissue architecture and reduced infiltration of mononuclear inflammatory cells (Fig 3E, S2 File). The histological changes were consistent with the behavioral and edema findings observed in CFA-induced inflammatory pain.

### *Cav1* knockout inhibited microglial activation in CFA-induced inflammatory pain

**Fig 4:**
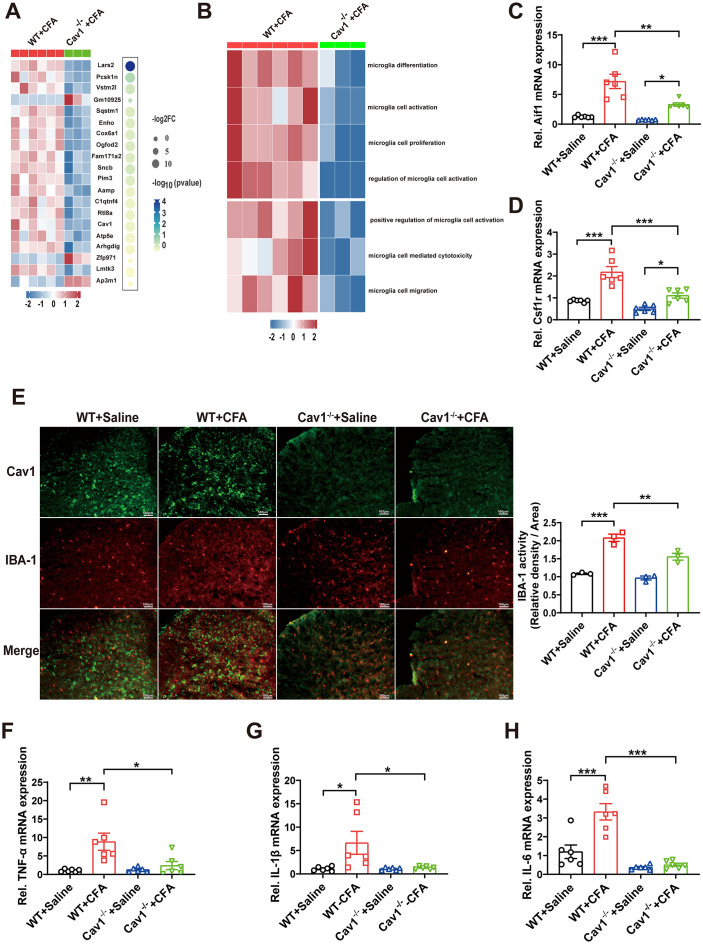
Cav1 deficiency inhibited microglial activation in CFA-induced inflammatory pain. **(A)** Heatmap analysis of differentially expressed genes associated with microglia signaling in spinal cord tissues of *Cav1*^*-/-*^ and WT mice 4 days after CFA injection. Key genes included *Sqstm1, Cox6a1, Sncb, Cav1, Enho,* and *Ap3m1.* **(B)** Single-sample genomic enrichment analysis (ssGSEA) of differentially expressed genes, showing enrichment in pathways related to microglial differentiation, activation, and proliferation. **(C-D)** RT-qPCR analysis of microglial activation markers *Aif1* and *Csf1r* in spinal cord tissues of WT and *Cav1*^*-/-*^ mice. Data are presented as mean ± SEM. *n* = 6 mice per group. **(E)** Representative immunofluorescence staining of IBA-1 in spinal cord tissues from WT and *Cav1 ⁻ / ⁻ *mice injected with CFA or saline. Scale bar = 100 μm. IBA-1 activity is shown as relative density/area. Data are presented as mean ± SEM; *n* = 3 mice per group. **(F-H)** RT-qPCR analysis of inflammatory cytokines *TNF-α, IL-1β,* and *IL-6* mRNA levels in the spinal cord tissues of WT and *Cav1*^*-/-*^ mice following CFA or saline injection. Data are presented as mean ± SEM. *n* = 6 mice per group. One-way ANOVA followed by Tukey’s post hoc test was performed for(C-E). *P < 0.05; **P < 0.01; ***P < 0.001.

To investigate the role of Cav1 in the dorsal horn of spinal cord after CFA injection, RNA sequencing (RNA-seq) was performed on spinal cord tissues from *Cav1*^*-/-*^ and WT mice 4 days post-CFA injection. Heatmap analysis revealed differentially expressed genes associated with microglial signaling, including *Sqstm1, Cox6a1,* and *Ap3m1* (Fig 4A). Single-sample genomic enrichment analysis (ssGSEA) further showed these genes were enriched in pathways related to microglial activation and proliferation (Fig 4B). RT-qPCR validation confirmed significant upregulation of microglial activation markers *Aif1* and *Csf1r* in WT + CFA mice, which was markedly attenuated in *Cav1*^*-/-*^ mice (Fig 4C and 4D, S2 File). Immunofluorescence staining showed increased IBA-1-positive microglia in WT + CFA mice, while this increase was reduced in *Cav1*^*-/-*^ mice (Fig 4E, S2 File). Additionally, pro-inflammatory cytokines *TNF-α, IL-1β,* and *IL-6* were significantly elevated in WT + CFA mice but showed reduced expression in *Cav1*^*-/-*^ mice (Fig 4F-4H, S2 File). RT-qPCR and immunofluorescence results collectively highlighted the role of Cav1 in modulating microglial activation and inflammatory cytokine production during CFA-induced pain.

### Cav1 modulated the cGAS-STING pathway and autophagy in inflammatory pain

**Fig 5:**
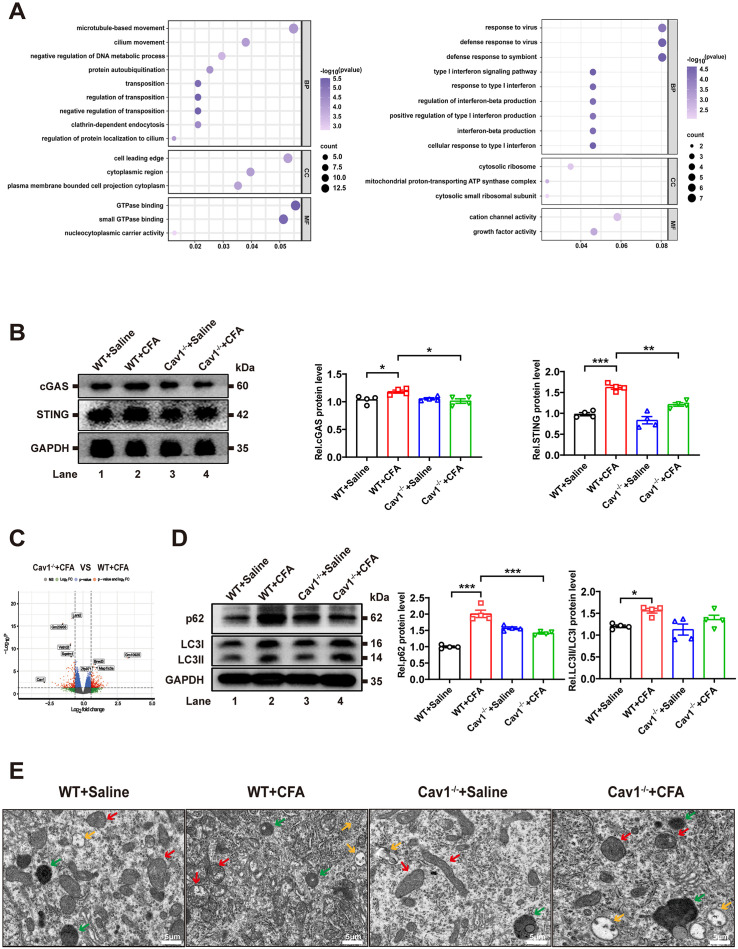
Cav1 modulated the cGAS-STING pathway and autophagy in inflammatory pain. **(A)** GO enrichment analysis of upregulated genes in *Cav1*^*-/-*^ mice after CFA treatment, showing enrichment in ciliary protein localization and protein self-ubiquitination (BP), cytoplasmic components of cell leading edge and membrane bundle (CC), and GTPase binding and nuclear transport carrier activity (MF) and GO enrichment analysis of downregulated genes, showing enrichment in type I interferon signaling and response (BP), cell membrane ribosome and mitochondrial H + -ATP synthase complex (CC), and cationic channel and growth factor activity (MF). **(B)** Western blot images of cGAS and STING protein expression in the spinal cord tissues of wild-type (WT) and *Cav1*^*-/-*^ mice following CFA or saline injection. Data are presented as mean ± SEM. *n* = 4 mice per group. One-way ANOVA followed by Tukey’s post hoc test was performed. **(C)** Volcano plot analysis of differentially expressed genes between *Cav1*^*-/-*^ + CFA and WT + CFA groups, highlighting *Sqstm1* and *Map1lc3a* as closely associated with autophagy. **(D)** Western blot images of autophagy markers LC3 and p62 in the spinal cord tissues of wild-type (WT) and *Cav1*^*-/-*^ mice following CFA or saline injection. Data are presented as mean ± SEM. *n* = 4 mice per group. One-way ANOVA followed by Tukey’s post hoc test was performed. **(E)** TEM of mitochondria (red arrowheads), lysosomes (green arrowheads), and autophagosomes (yellow arrowheads). scale bar = 5 μm. *P < 0.05; **P < 0.01; ***P < 0.001.

To further investigate the role of Cav1 in the dorsal horn of spinal cord after CFA injection, we performed GO enrichment analysis on differentially expressed genes (DEGs) from *Cav1*^*-/-*^ and WT mice after CFA treatment. Upregulated DEGs were associated with protein ubiquitination and GTPase binding, while downregulated DEGs were linked to type I interferon signaling (Fig 5A). These findings led us to focus on the cGAS-STING pathway, which was crucial in inflammation and closely associated with protein ubiquitination.

To test this, we analyzed cGAS and STING expression in spinal cord tissues. Compared with WT+Saline mice, WT + CFA mice showed significantly increased cGAS and STING expression, which was markedly reduced in *Cav1*^*-/-*^ -CFA mice (Fig 5B, S1 Raw Images, and S2 File). This supported the hypothesis that Cav1 modulated inflammatory pain through the cGAS-STING pathway.

Further analysis revealed significant alterations in autophagy-related genes *Sqstm1* and *Map1lc3a* in WT + CFA mice (Fig 5C). Consistent with these findings, protein analysis showed increased p62 expression and LC3-II/LC3-I ratio in WT + CFA mice, indicating impaired autophagic flux. In contrast, *Cav1*^*-/-*^ + CFA mice exhibited reduced p62 levels and LC3-II/LC3-I ratio, suggesting enhanced autophagy activity (Fig 5D, S1 Raw Images, S2 File).

Transmission electron microscopy (TEM) provided ultrastructural evidence supporting these molecular findings. WT + CFA mice displayed a massive accumulation of autophagosomes with impaired fusion to lysosomes and increased abnormal mitochondria. In contrast, *Cav1*^*-/-*^ -CFA mice exhibited enhanced autophagosome-lysosome fusion and fewer abnormal mitochondria, indicating restored autophagic flux (Fig 5E). These ultrastructural changes corroborated the molecular data and highlighted autophagy restoration as a key mechanism underlying Cav1 deficiency’s anti-inflammatory effects.

### Overexpression of Cav1 enhanced inflammation through cGAS-STING activation and autophagy inhibition

**Fig 6:**
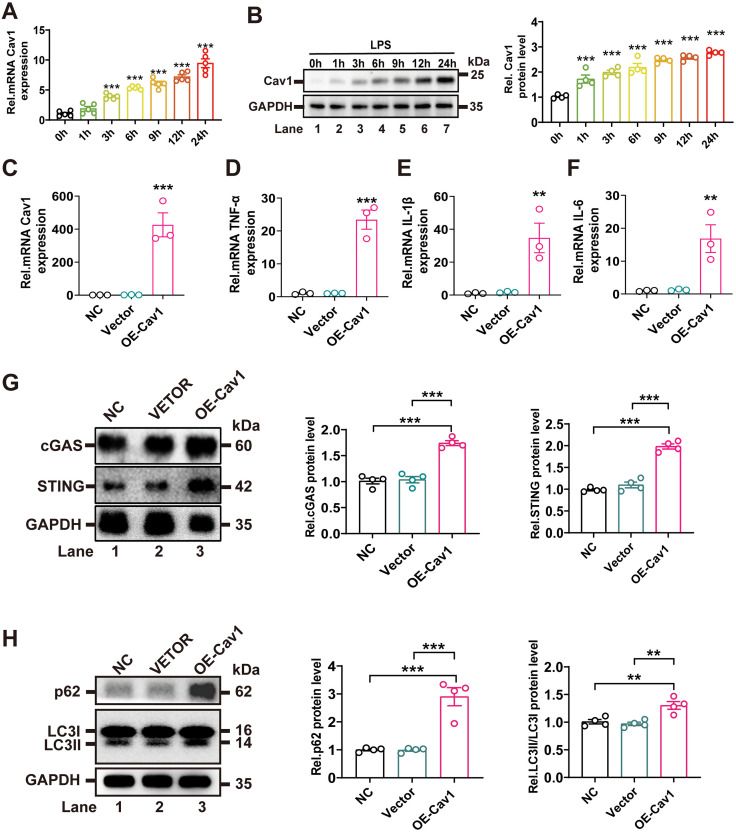
Overexpression of Cav1 enhanced inflammation through cGAS-STING activation and autophagy inhibition. **(A)** RT-qPCR analysis of *Cav1* mRNA levels in BV2 microglial cells stimulated with LPS for 0, 1, 3, 6, 9, 12, and 24 hours. Data are presented as mean ± SEM. *n* = 5 independent samples per time point. **(B)** Western blot analysis of Cav1 protein levels in BV2 microglial cells stimulated with LPS for 0, 1, 3, 6, 9, 12, and 24 hours. Data are presented as mean ± SEM. *n* = 4 independent samples per time point. **(C)** RT-qPCR confirmation of Cav1 overexpression in Cav1-overexpressing (OE-Cav1) BV2 microglial cells. Data are presented as mean ± SEM. *n* = 3 independent samples per group. **(D-F)** RT-qPCR analysis of proinflammatory cytokine mRNA levels (*TNF-α, IL-1β, and IL-6*) in *OE-Cav1* and control BV2 microglial cells. Data are presented as mean ± SEM. *n* = 3 independent samples per group. **(G)** Western blot analysis of cGAS and STING protein expression in *OE-Cav1* and control BV2 microglial cells. Quantification of protein levels is shown. Data are presented as mean ± SEM. *n* = 4 independent samples per group. **(H)** Western blot analysis of LC3 and p62 protein expression in *OE-Cav1* and control BV2 microglial cells. Quantification of the LC3-II/LC3-I ratio and p62 protein levels is shown. Data are presented as mean ± SEM. *n* = 4 independent samples per group. One-way ANOVA followed by Tukey’s post hoc test was performed. **P < 0.01; ***P < 0.001.

To explore the molecular mechanisms underlying Cav1-mediated microglial activation, BV2 murine microglial cells were used for in vitro experiments. In the LPS-induced BV2 microglial inflammation model, Cav1 mRNA and protein expression levels significantly increased over time (Fig 6A and 6B, S1 Raw Images, and S2 File). To investigate Cav1’s role, BV2 cells were transiently transfected with a *pcDNA3.1-Cav1* plasmid (OE-Cav1). RT-qPCR confirmed successful *Cav1* mRNA overexpression (Fig 6C, S2 File). Overexpression of *Cav1* significantly increased the mRNA levels of pro-inflammatory cytokines *TNF-α, IL-1β, and IL-6* (Fig 6D–6F, S2 File), indicating amplified pro-inflammatory signaling.

Western blot analysis revealed that OE-Cav1 cells exhibited significantly upregulated cGAS and STING protein levels (Fig 6G, S1 Raw Images, and S2 File), suggesting that Cav1 enhanced inflammation through the cGAS-STING pathway. Additionally, autophagy marker analysis showed a decreased LC3-II/LC3-I ratio and increased p62 protein levels (Fig 6H, S1 Raw Images, and S2 File), indicating suppressed autophagic flux. These findings demonstrated that Cav1 promoted inflammation by activating the cGAS-STING pathway and inhibiting autophagy.

### si-Cav1 alleviated inflammation by inhibiting the cGAS-STING pathway and activating autophagy

**Fig 7:**
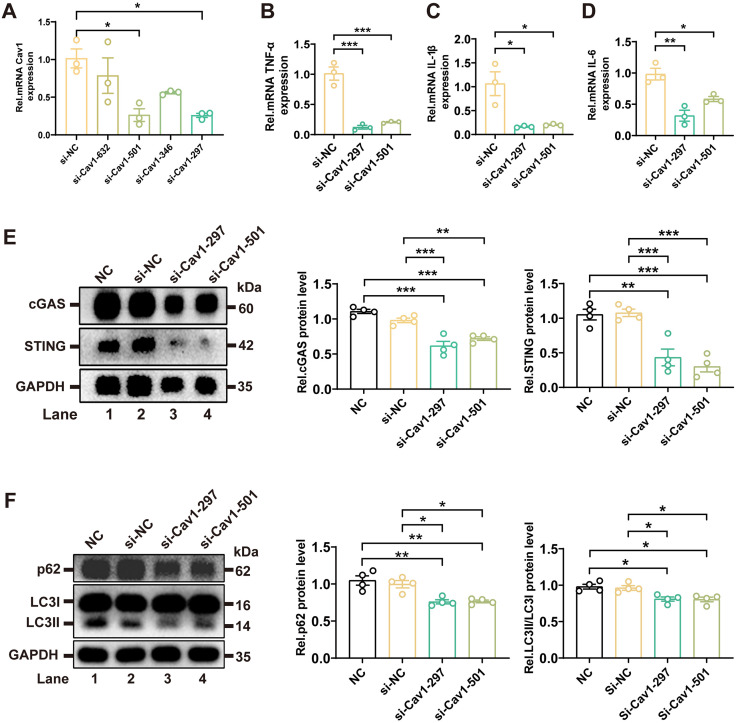
si-Cav1 alleviated inflammation by inhibiting the cGAS-STING pathway and activating autophagy. **(A)** RT-qPCR analysis of *Cav1* mRNA levels in BV2 microglial cells transfected with different Cav1-targeting siRNAs. Data are presented as mean ± SEM. *n* = 3 independent samples per group. **(B-D)** RT-qPCR analysis of pro-inflammatory cytokine mRNA levels (*TNF-α, IL-1β, and IL-6)* in BV2 microglial cells transfected with *si-501-Cav1 or si-297-Cav1.* Data are presented as mean ± SEM. *n* = 3 independent samples per group. **(E)** Western blot analysis of cGAS and STING protein expression in BV2 microglial cells transfected with *si-501-Cav1 or si-297-Cav1.* Quantification of protein levels is shown. Data are presented as mean ± SEM. *n* = 4 independent samples per group. **(F)** Western blot analysis of autophagy markers LC3 and p62 in BV2 microglial cells transfected with *si-501-Cav1 or si-297-Cav1*. Quantification of the LC3-II/LC3-I ratio and p62 protein levels is shown. Data are presented as mean ± SEM. *n* = 4 independent samples per group. One-way ANOVA followed by Tukey’s post hoc test was performed. *P < 0.05; **P < 0.01; ***P < 0.001.

To validate the role of Cav1, we designed four siRNAs targeting Cav1 in BV2 cells and identified two effective siRNAs (*si-501-Cav1 and si-297-Cav1)* that significantly reduced Cav1 mRNA levels (Fig 7A, S2 File). Knockdown of Cav1 significantly decreased the mRNA levels of *TNF-α, IL-1β, and IL-6* (Fig 7B–7D, S2 File), indicating suppression of pro-inflammatory signaling.

Western blot analysis showed that Cav1 knockdown markedly reduced cGAS and STING protein levels (Fig 7E, S1 Raw Images, and S2 File), supporting the hypothesis that Cav1 regulated inflammation via the cGAS-STING pathway. Furthermore, autophagy marker analysis revealed a significant increase in the LC3-II/LC3-I ratio and a decrease in p62 protein levels in si-Cav1-transfected cells (Fig 7F, S1 Raw Images, and S2 File), suggesting enhanced autophagic activity.

Collectively, these results demonstrated that Cav1 knockdown alleviated inflammation by inhibiting the cGAS-STING pathway and promoting autophagic flux, providing new insights into Cav1’s role in inflammatory pain.

## Discussion

Our study revealed a novel regulatory role of Cav1 in inflammatory pain, providing critical insights into the molecular mechanisms underlying this pathological condition. In a CFA-induced inflammatory pain model, we observed a significant upregulation of Cav1 expression in the spinal cord dorsal horn, which correlated with pronounced pain behaviors. Meanwhile, over-expressed Cav1 activated the cGAS-STING signaling pathway, inhibited autophagy, amplified microglial pro-inflammatory responses. In contrast, Cav1^-/-^ mice exhibited attenuated pain behaviors, inactivated the cGAS-STING signaling pathway, augmented autophagy and reduced inflammatory responses. The above researches supported the pivotal role of Cav1 in inflammatory pain modulation.

Interestingly, we found that Cav1 deficiency significantly alleviated CFA-induced mechanical allodynia but did not affect thermal hyperalgesia. This modality-specific effect suggests that Cav1 may selectively modulate mechanical pain pathways. Mechanical and thermal nociception are mediated by distinct types of primary afferent fibers—Aβ- and Aδ-fibers for mechanical stimuli, and C-fibers for thermal stimuli [22,23]. It is plausible that Cav1 preferentially influences mechanosensitive pathways, possibly through differential regulation of spinal microglial activation. Further investigations are warranted to elucidate the precise mechanisms underlying this selective effect.

The pathogenesis of inflammatory pain is closely related to microglial activation and the release of pro-inflammatory mediators [24–26]. In our study, we observed significant activation of microglia and upregulation of Cav1 expression in the spinal cord dorsal horn following CFA-induced inflammatory pain. Cav1 deficiency inhibited microglial activation, attenuated the expression of inflammatory cytokines **(TNF-*α, *IL-1*β, *IL-6)** in the spinal cord and in vitro, reduced inflammatory pain behavior. Consistent with our results, Cav1 has been reported to exhibit pro-inflammatory effects in various central nervous system pathologies [27,28]. It was a key component of microglial activation, which may be related to increased vascular permeability, Th1 cell infiltration [29] and so on.

Additionally, we discovered an association between Cav1 and the cGAS-STING pathway in inflammatory pain, representing a novel mechanism in pain regulation. This finding was supported by GO analysis, which highlighted the involvement of type I interferon signaling and protein ubiquitination processes. Further investigations demonstrated that Cav1 upregulation activates the cGAS-STING pathway, resulting in enhanced production of pro-inflammatory cytokines. This observation aligns with findings in a multiple sclerosis model by Zhang et al [30–32]. suggesting an important role of this pathway in neuroinflammation. It provides novel insights into how innate immune responses contribute to inflammatory pain sensitization.

In exploring the mechanistic role of Cav1, we uncovered an inverse relationship between Cav1 expression and autophagy. Cav1 deficiency enhanced autophagy, contributing to reduced inflammatory responses, consistent with the results of Zhang et al [12]. Conversely, Cav1 overexpression inhibited autophagic flux, consistent with Xu Q et al.’s observations in hepatocellular carcinoma [33]. Notably, recent studies have shown that inducing autophagy has analgesic effects in various pain models, including neuropathic pain [34–36]. Our research opens new avenues for developing pain treatment strategies targeting Cav1 and autophagy.

However, in our CFA-induced inflammatory pain model, we observed that Cav1 overexpression was associated with both the activation of the cGAS-STING pathway and the suppression of autophagic flux in spinal microglia, which is contrary to previous reports [37–40]. They discovered that cGAS-STING activation can promote autophagy. This discrepancy may be attributed to context-specific regulatory mechanisms. For instance, Cav1 may inhibit autophagy through alternative pathways, such as enhanced autophagy in aortic endothelial cells or impairment of autophagosome-lysosome fusion [12,41], thereby overriding STING-mediated autophagy induction. Additionally, prolonged or excessive STING activation under chronic neuroinflammatory conditions may result in ER stress and autophagy dysfunction [41,42]. It is also possible that the effects of cGAS-STING signaling on autophagy differ depending on cell type, stimulus strength, and duration [43–45]. Therefore, we propose that the autophagy inhibition observed in our model reflects a pathological, context-dependent response specific to microglia in the setting of inflammatory pain.

Our study has elucidated the role of Cav1 in inflammatory pain for the first time, yet several crucial questions remain to be addressed in future research. Primarily, the cellular subtypes of Cav1 that affect inflammatory pain need to be identified. Furthermore, translating our findings from BV2 cells to in vivo environments necessitates further validation, including functional evaluations across diverse pain models. Lastly, developing small molecule inhibitors or gene therapy strategies targeting Cav1, and evaluating their efficacy across various administration routes and pain models. These research efforts will not only deepen our understanding of Cav1’s role in pain modulation but may also provide crucial insights for developing novel Cav1-targeted pain management therapies.

## Conclusions

Cav1 effectively alleviated inflammatory pain through modulation of the cGAS-STING pathway, autophagy and neuroinflammation in the spinal cord dorsal horn. Cav1 was a potential therapeutic target for inflammatory pain.

## Supporting information

S1 Raw Images Raw images underlying all gel and Western blot data presented in the manuscript. (PDF)

S2 File Raw data underlying the quantitative results reported in the manuscript. (XLSX)
