# Supplementary material for: Caveolin-1 knockout improves CFA-induced inflammatory pain in adult mice through modulating the cGAS STING pathway and autophagy
Source: PLoS One. 2025 Oct 9;20(10):e0333646. doi: 10.1371/journal.pone.0333646 (PMC12510588; doi:10.1371/journal.pone.0333646)
Supplement: S1 Raw Images — (PDF) [file pone.0333646.s001.pdf]

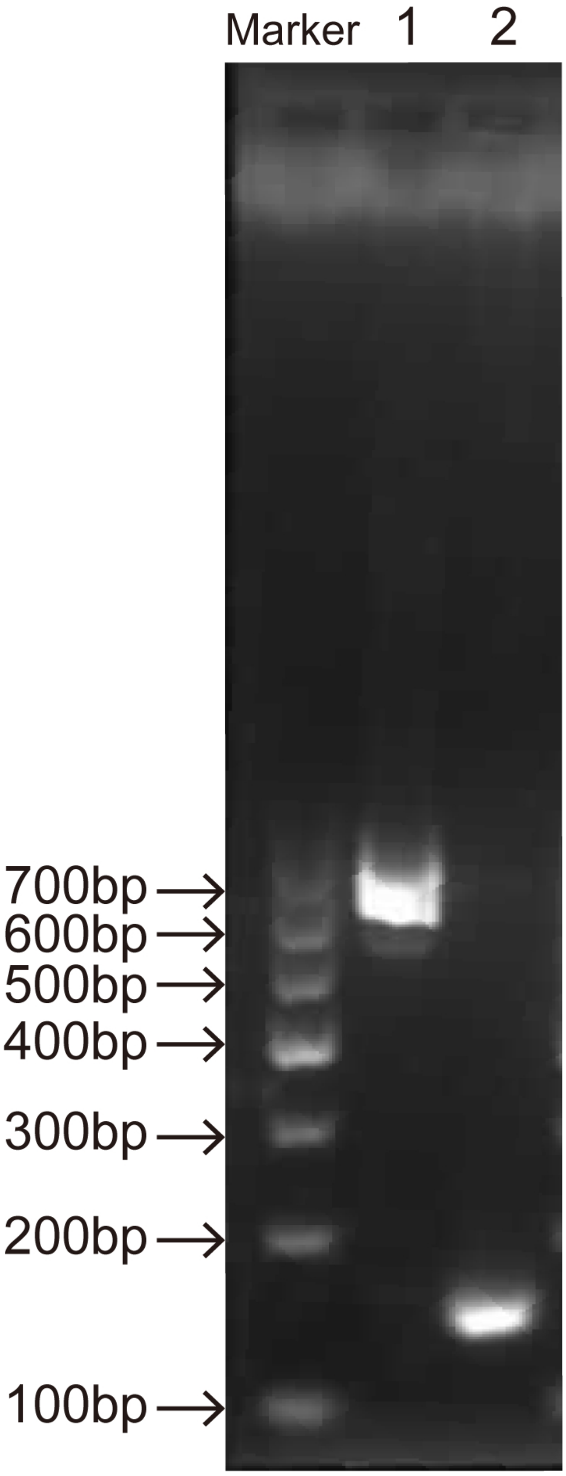

Agarose gel electrophoresis for identification of Cav1 knockout genotypes.

Representative agarose gel electrophoresis images of PCR products used to genotype Cav1 knockout mice. Lane 1 shows a 661 bp DNA fragment corresponding to the wild-type genotype; lanes 2 shows a 200 bp DNA fragment corresponding to the homozygous knockout genotype.
.

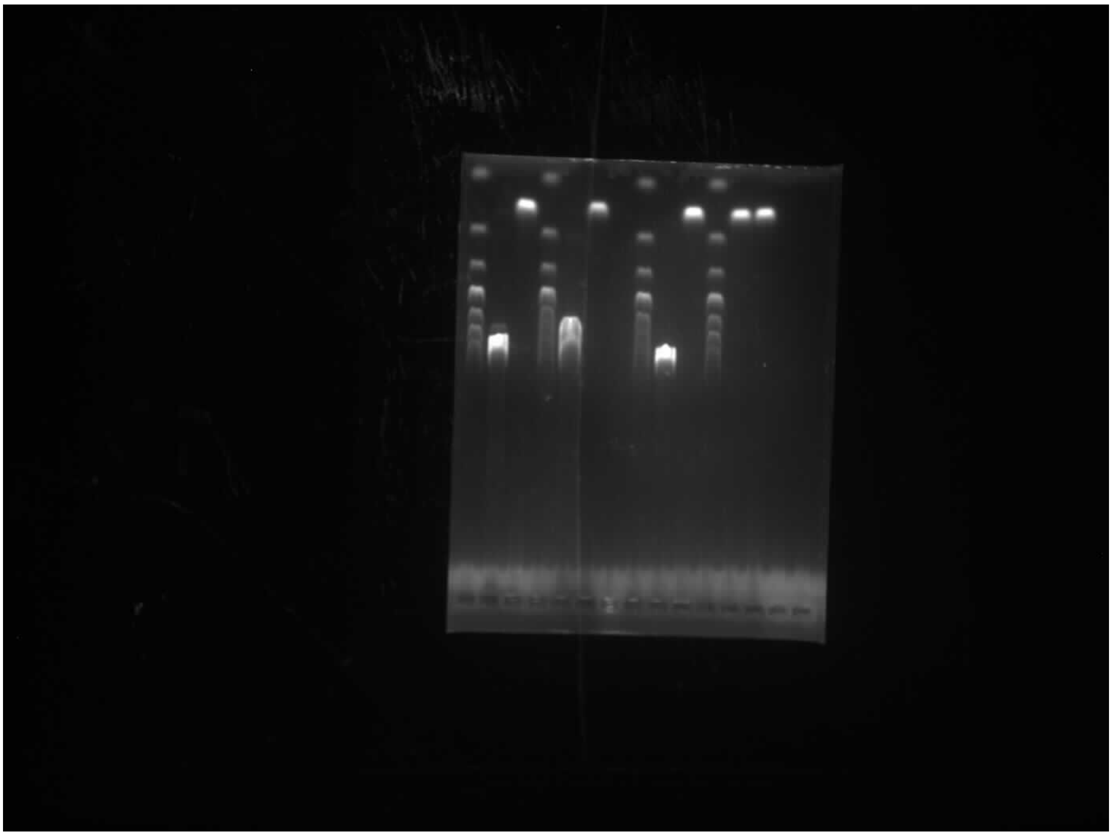

Original image of agarose gel

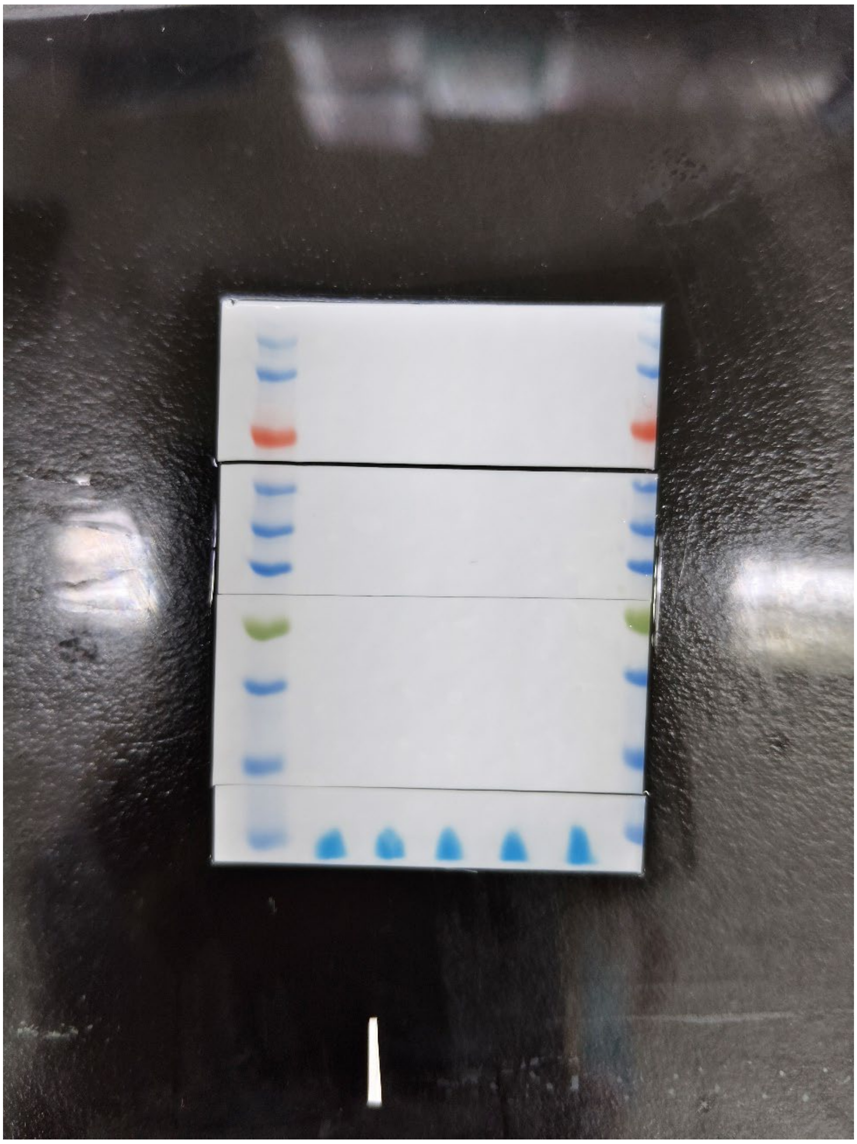

Fig2C-actin&Cav1-1-全膜

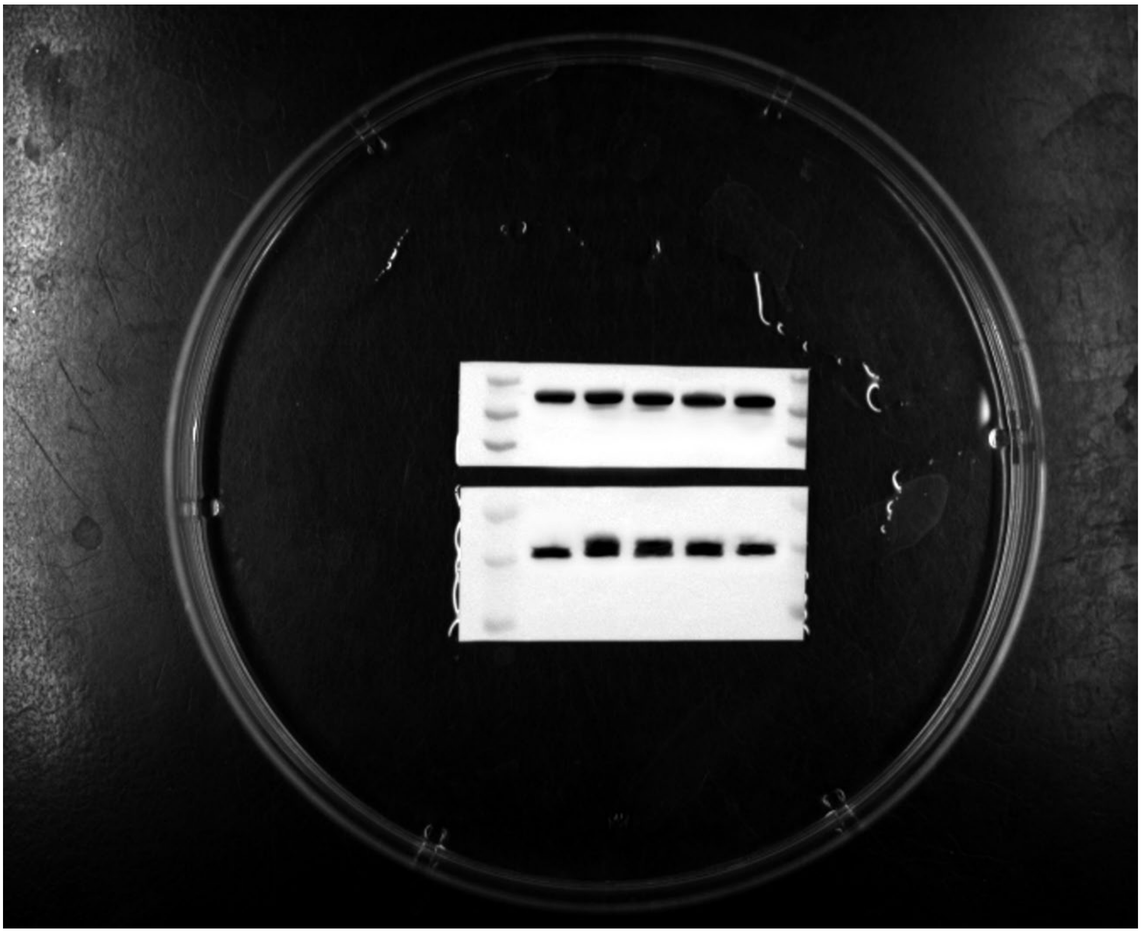

Fig2C-actin&Cav1-1

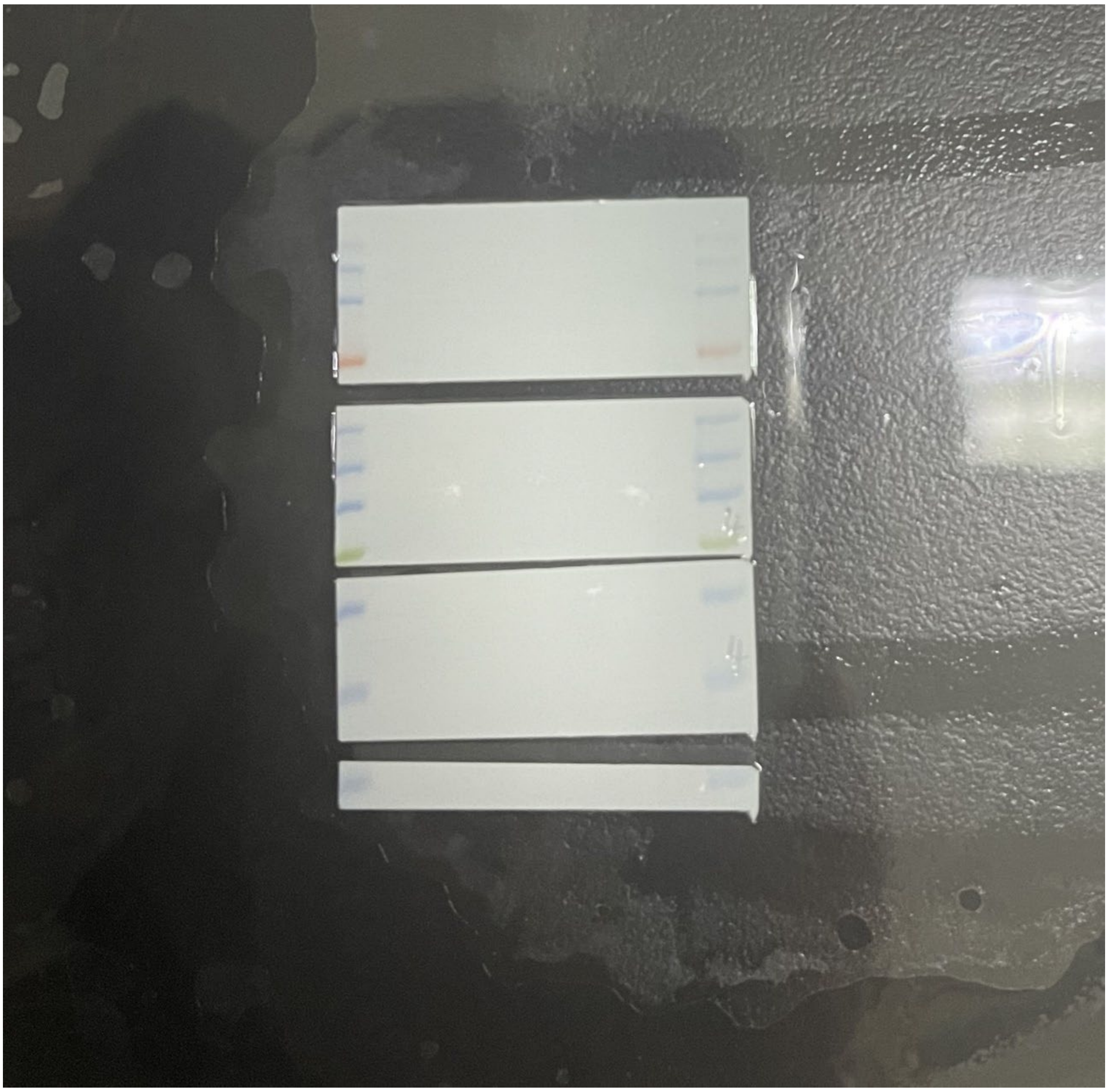

Fig2C-actin&Cav1-2-全膜

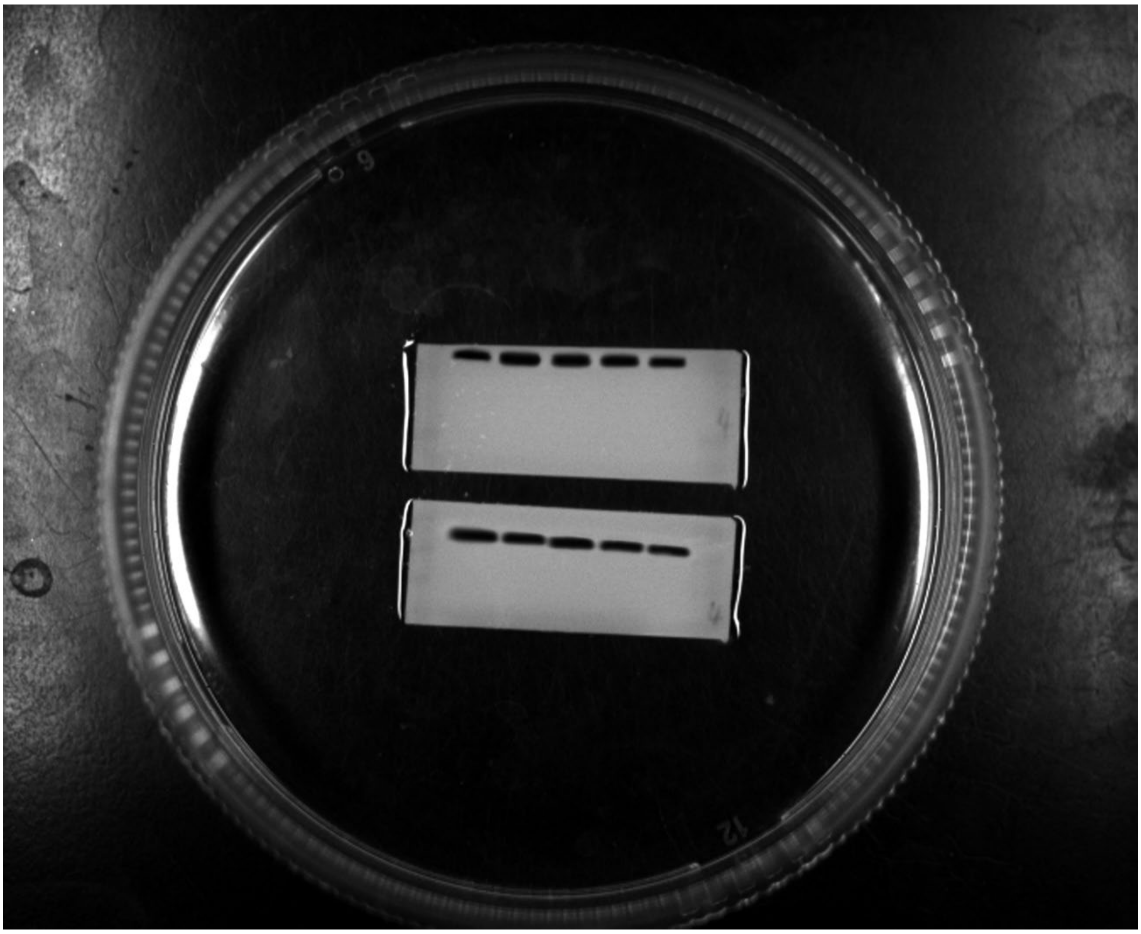

Fig2C-actin&Cav1-2

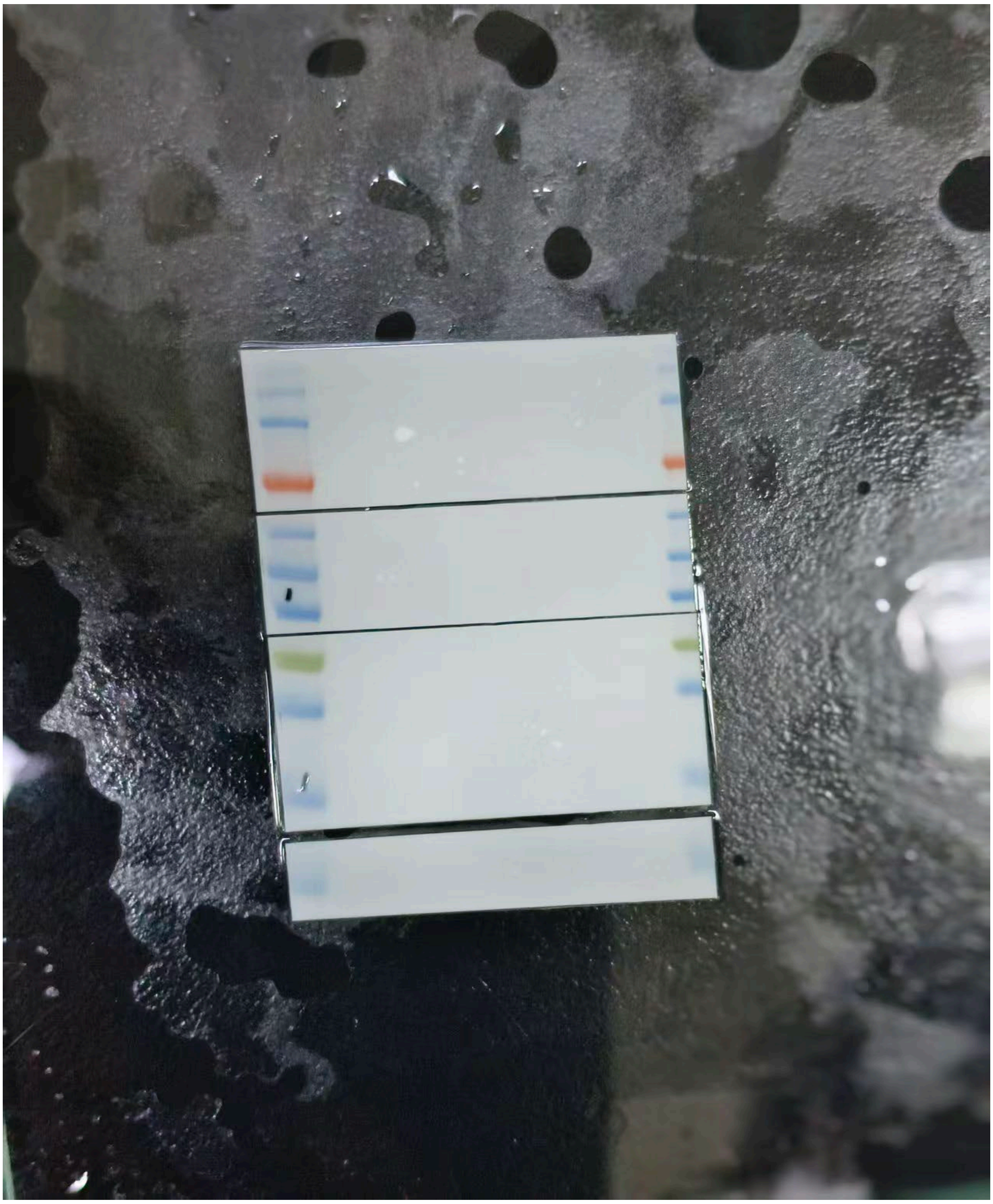

Fig2C-actin&Cav1-3-全膜

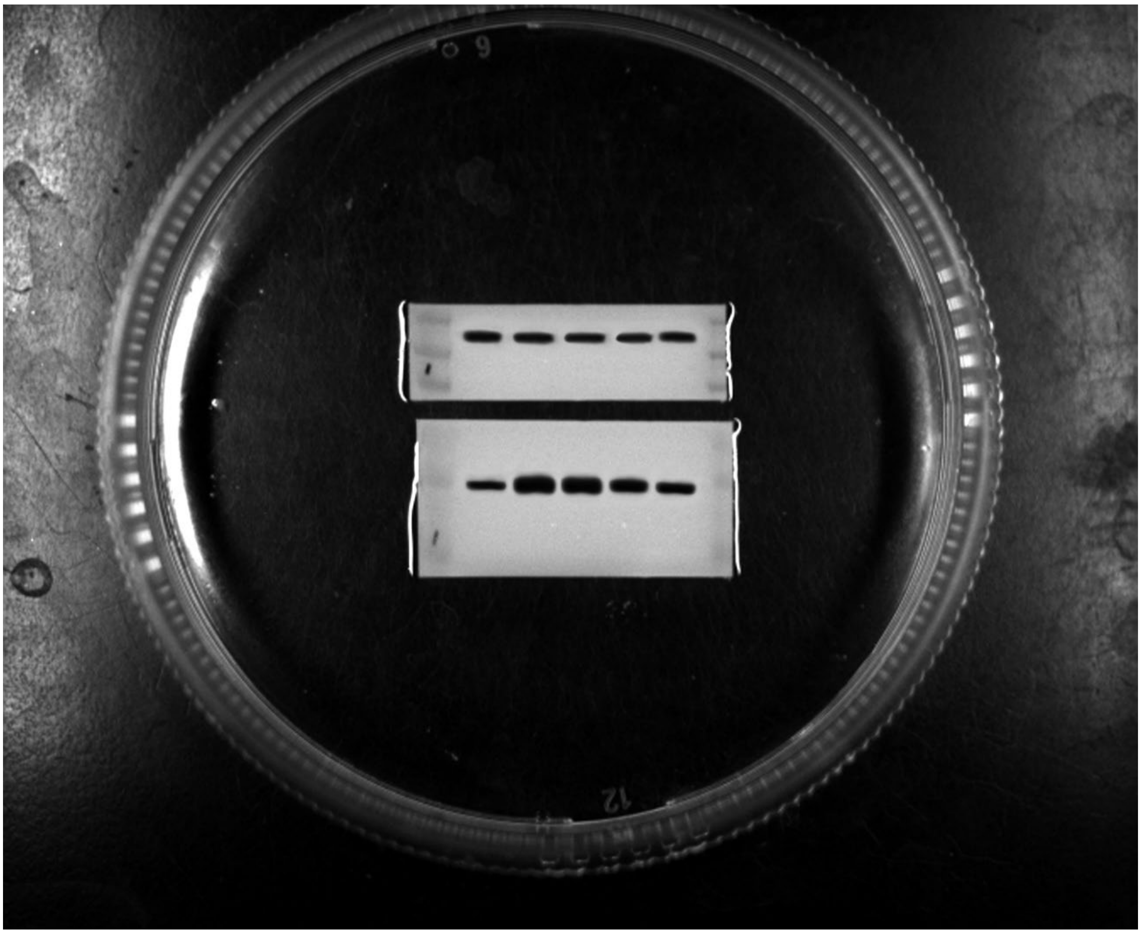

Fig2C-actin&Cav1-3

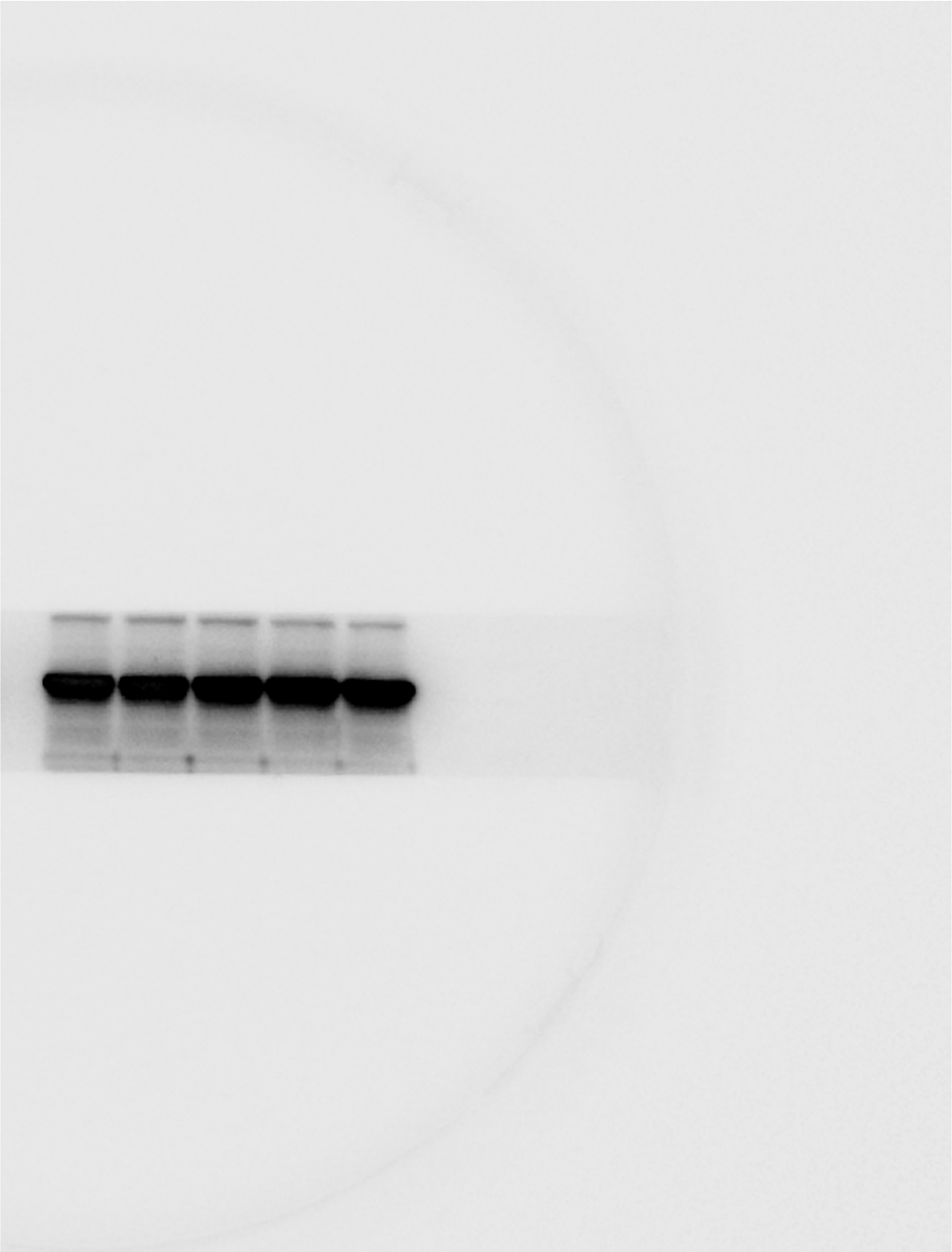

Fig2C-actin-4

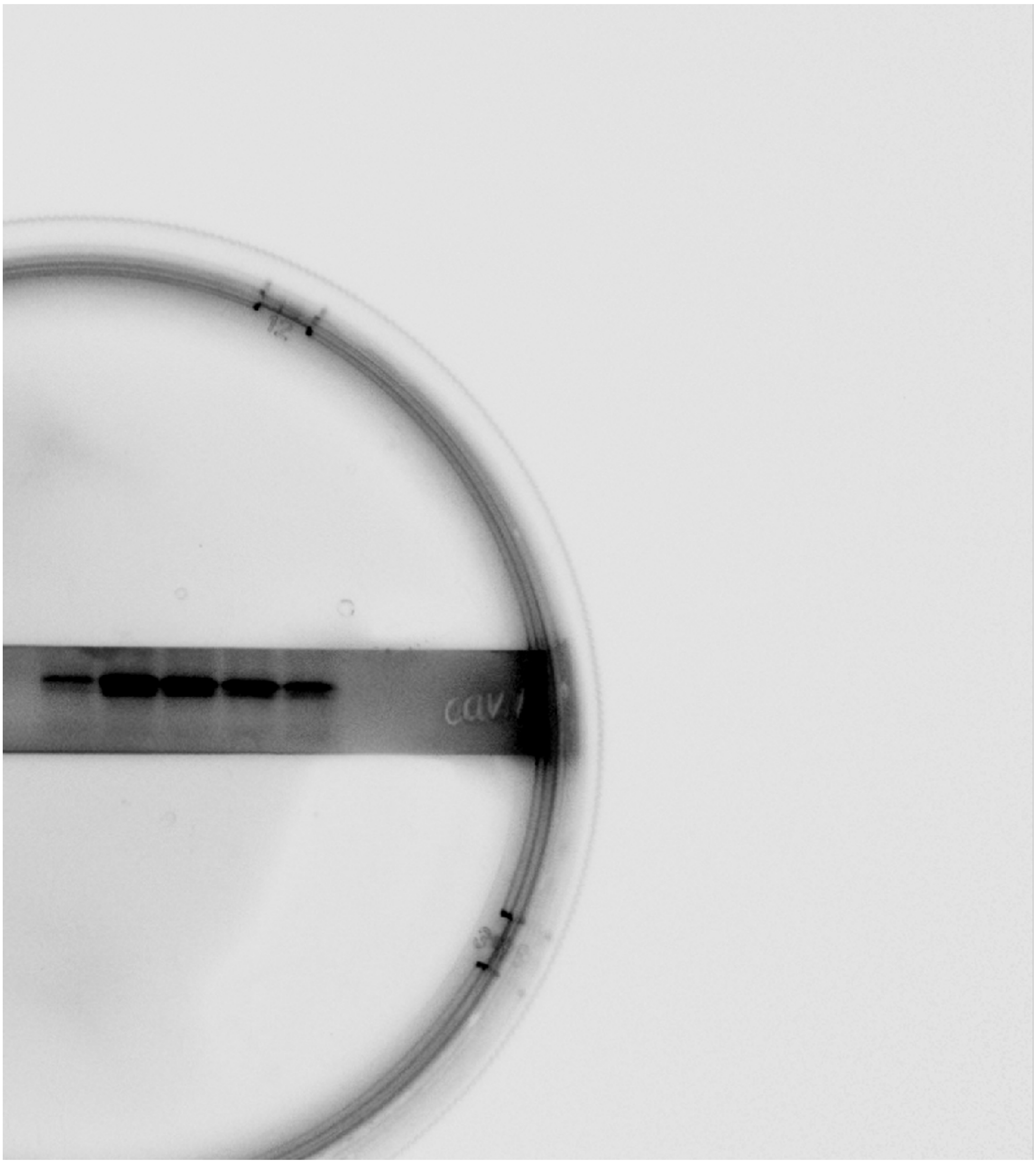

Fig2C-Cav1-4

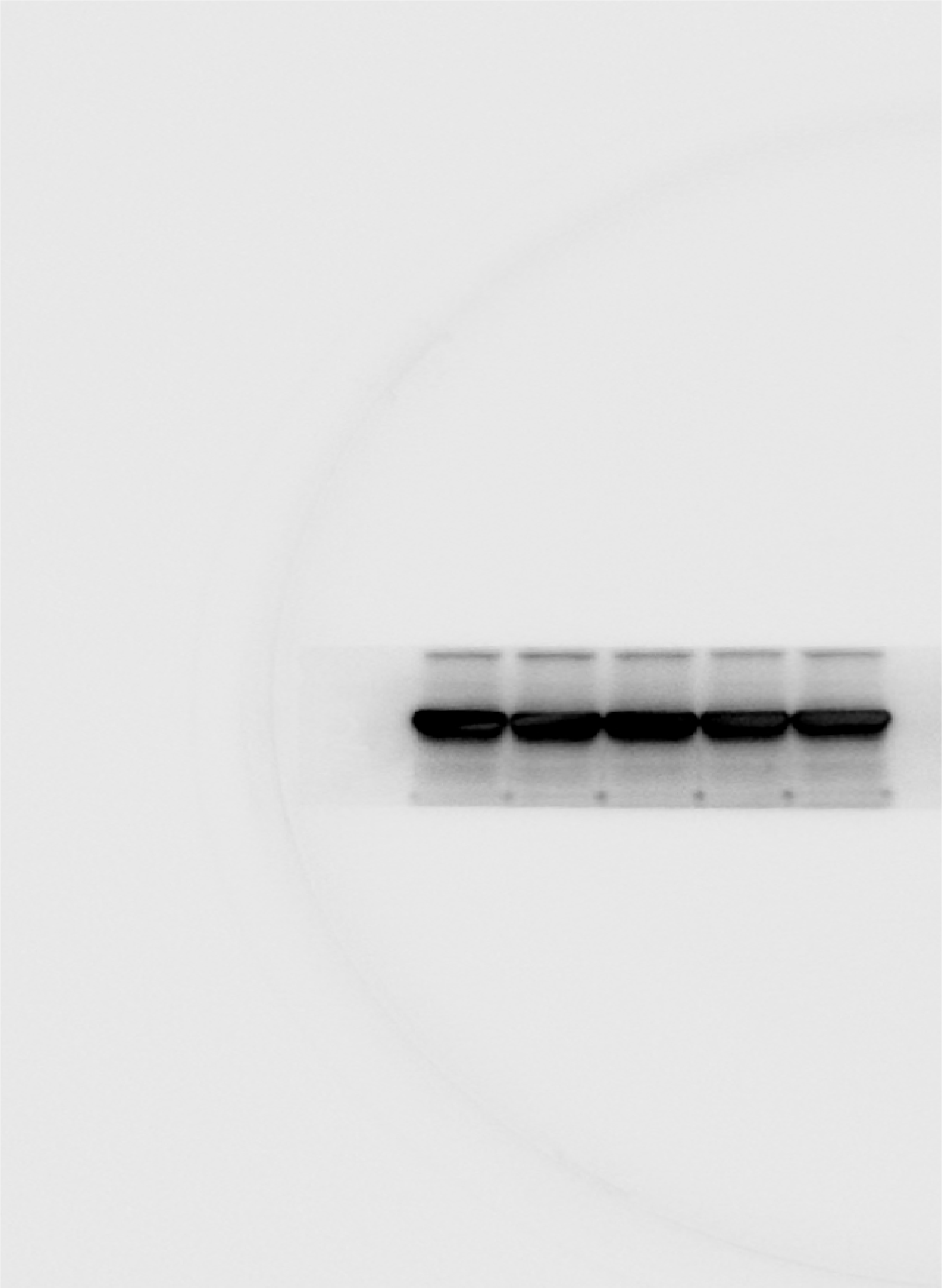

Fig2C-actin-5

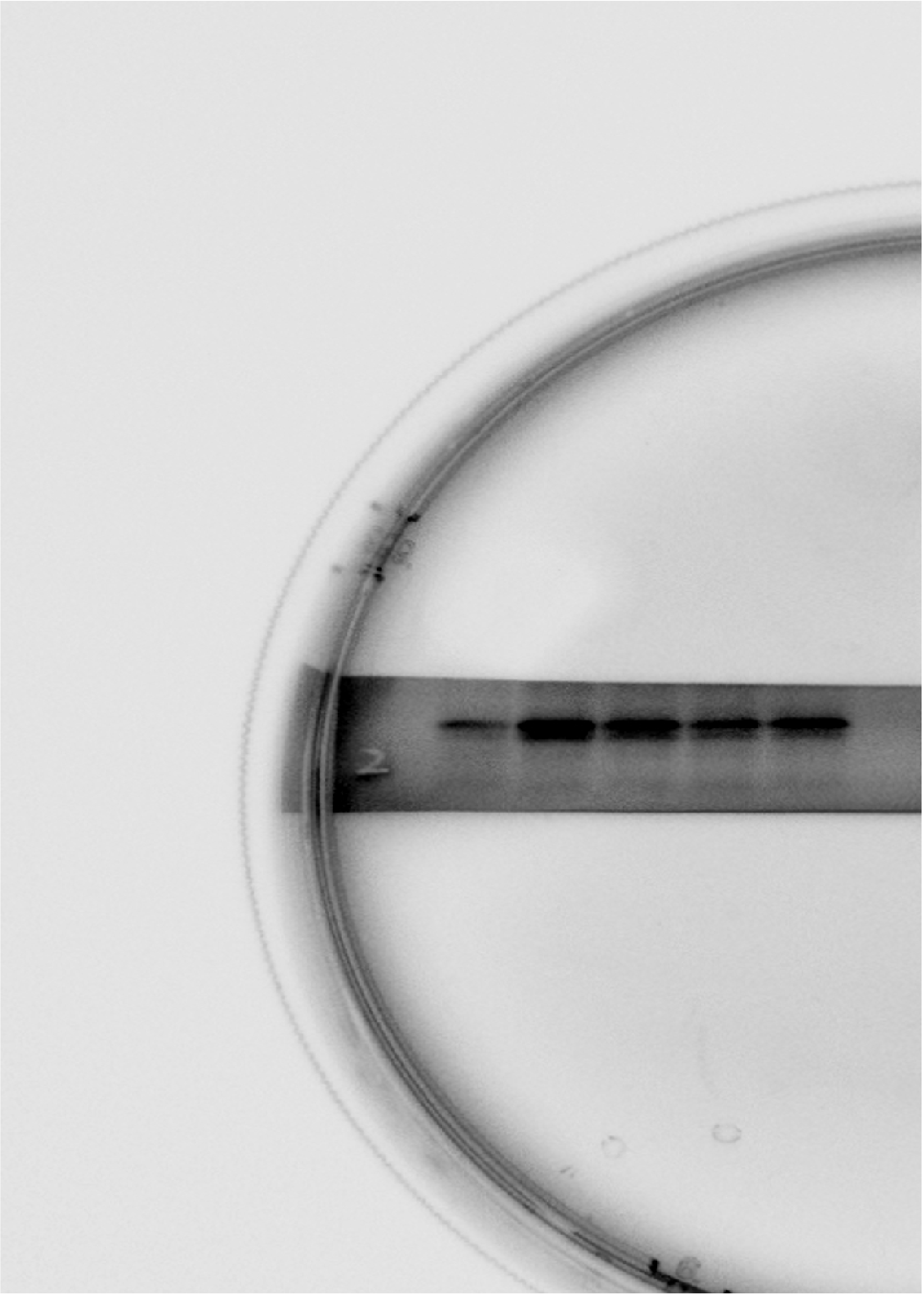

Fig2C-Cav1-5

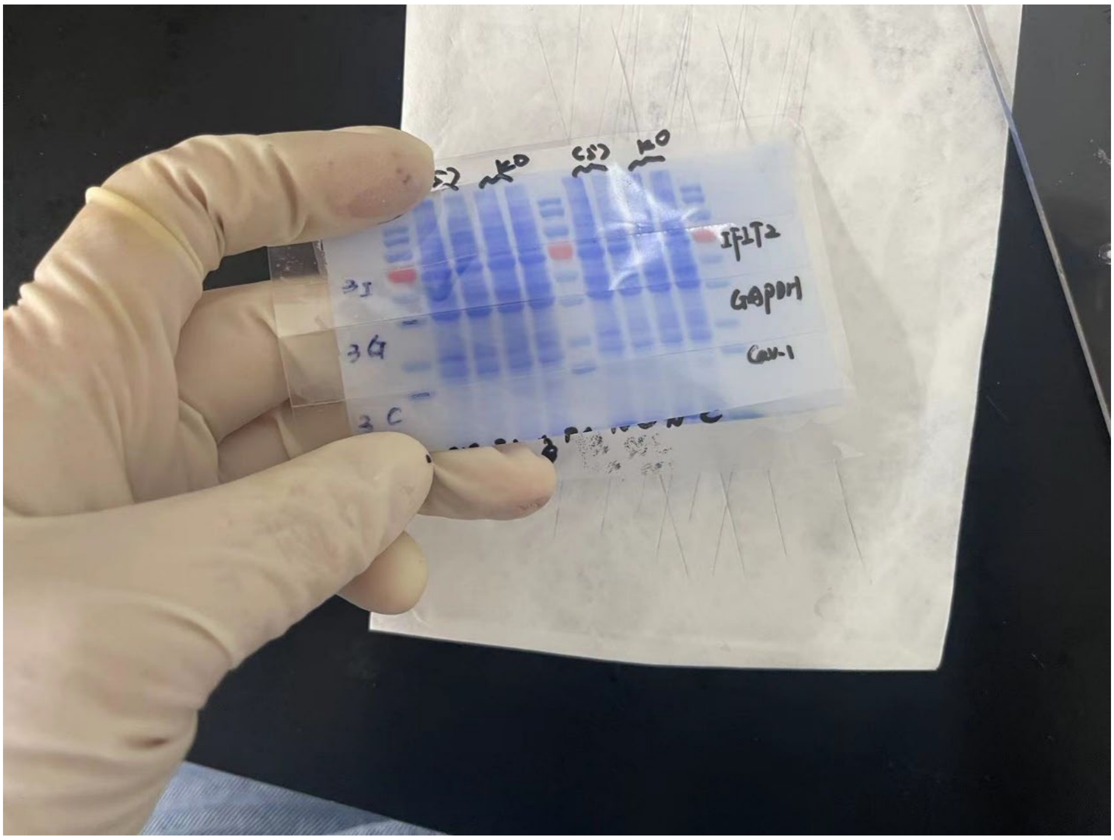

Fig2D-GAPDH&Cav1-全膜

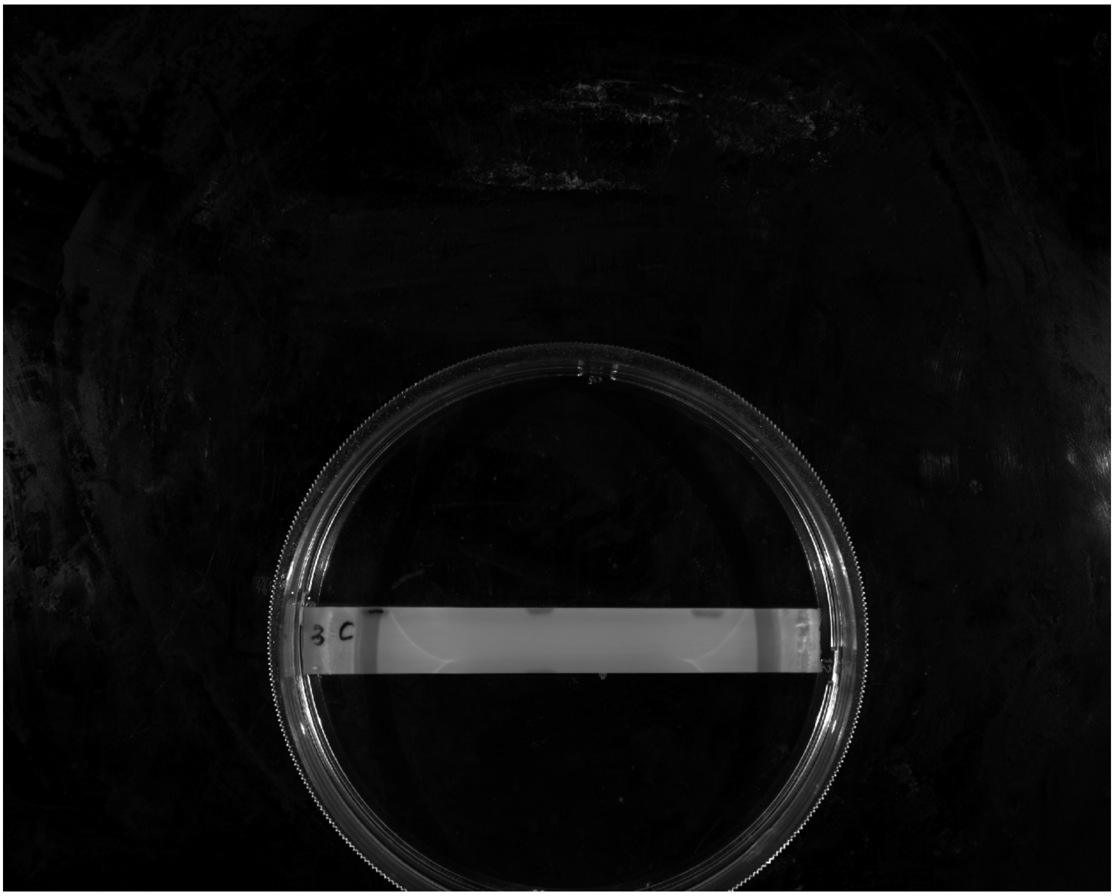

Fig2D-Cav1-white

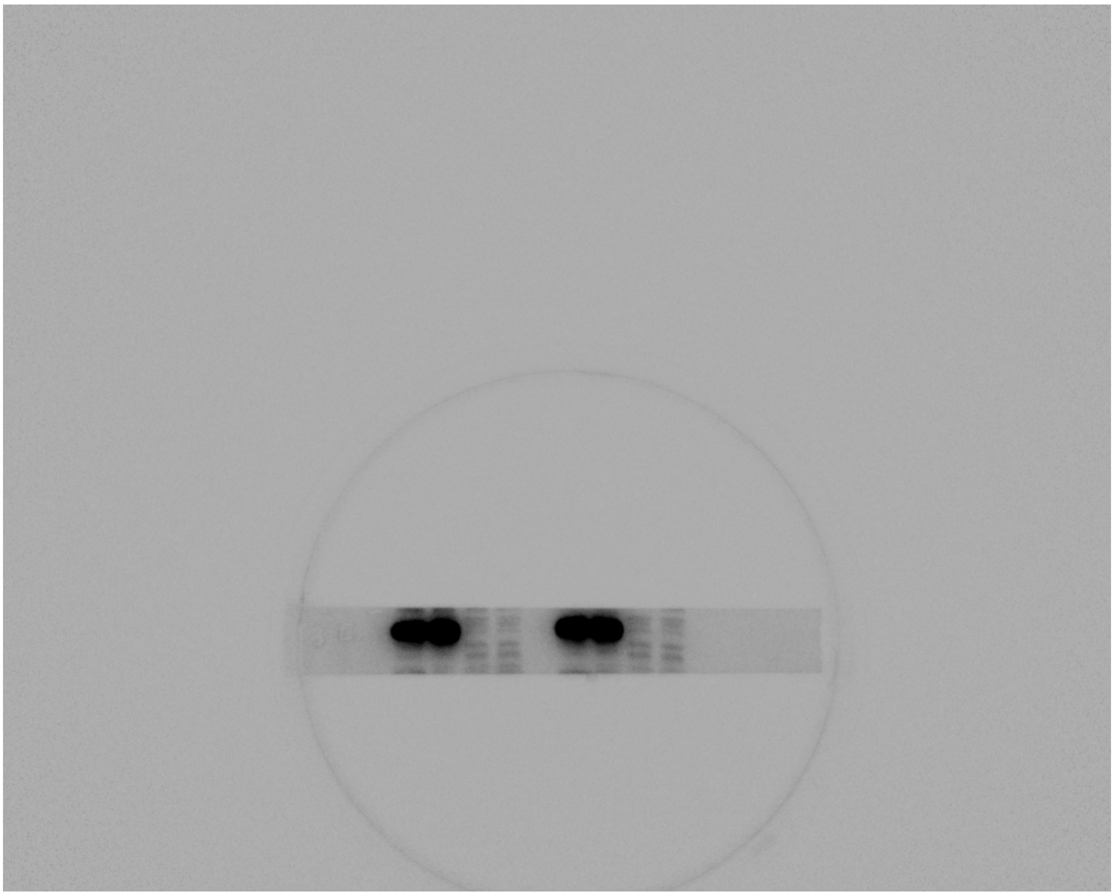

Fig2D-Cav1

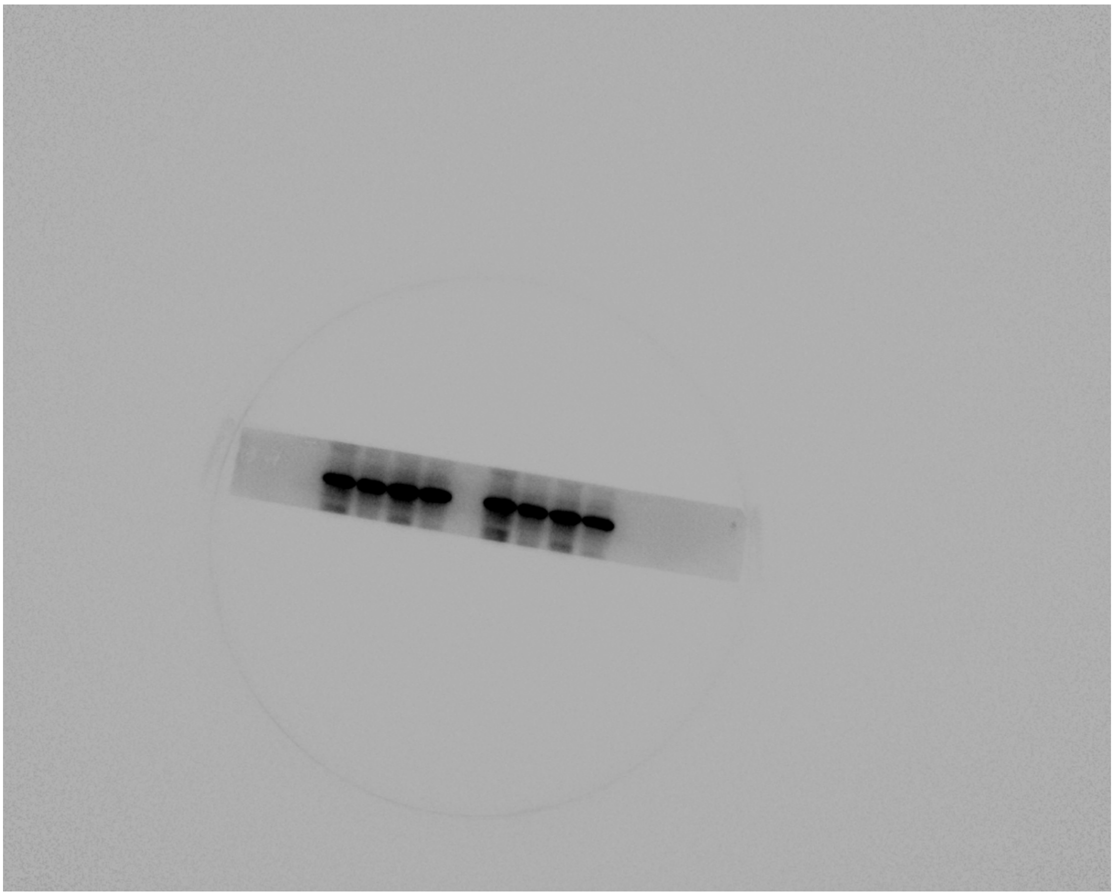

Fig2D-GAPDH

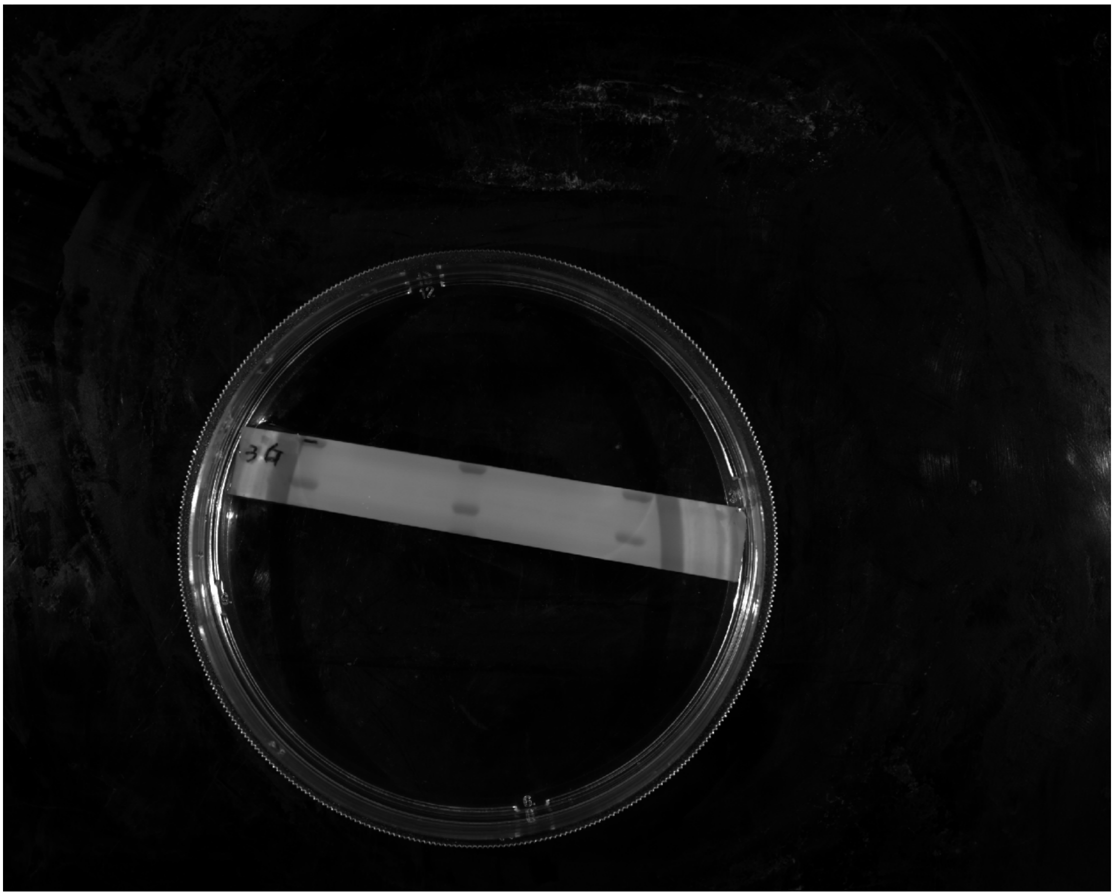

Fig2D-GAPDH-white

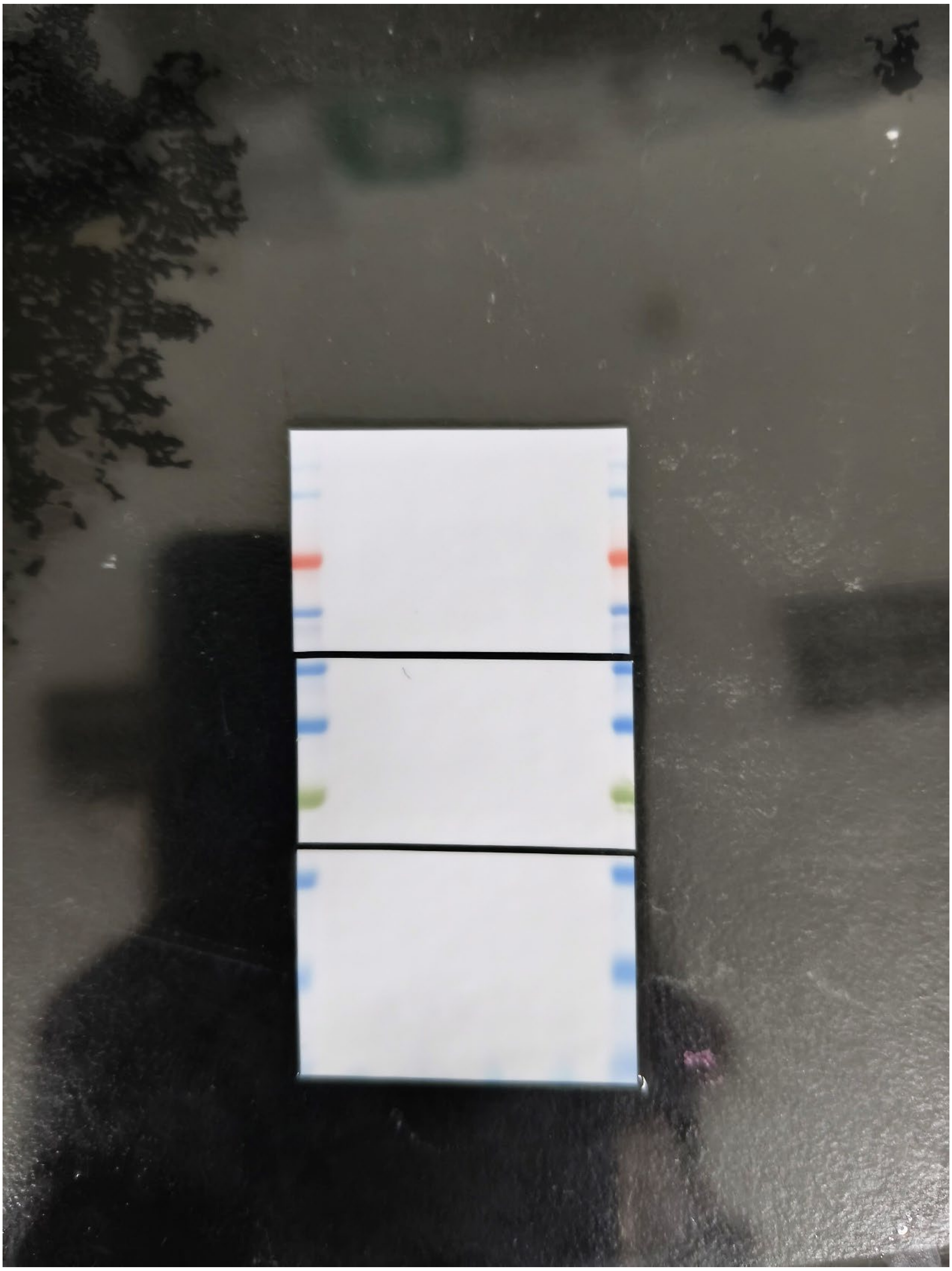

Fig5B-GAPDH&cGAS-1-全膜

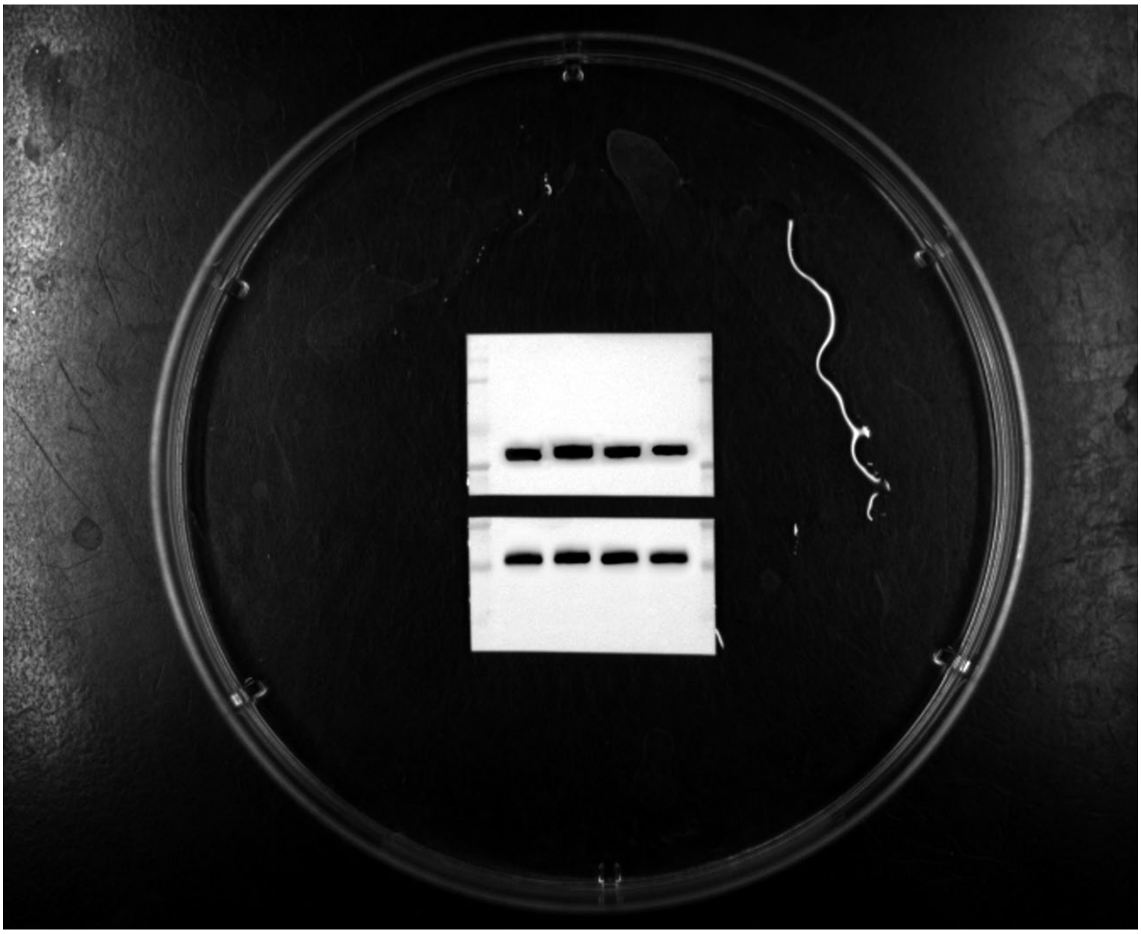

Fig5B-GAPDH&cGAS-1

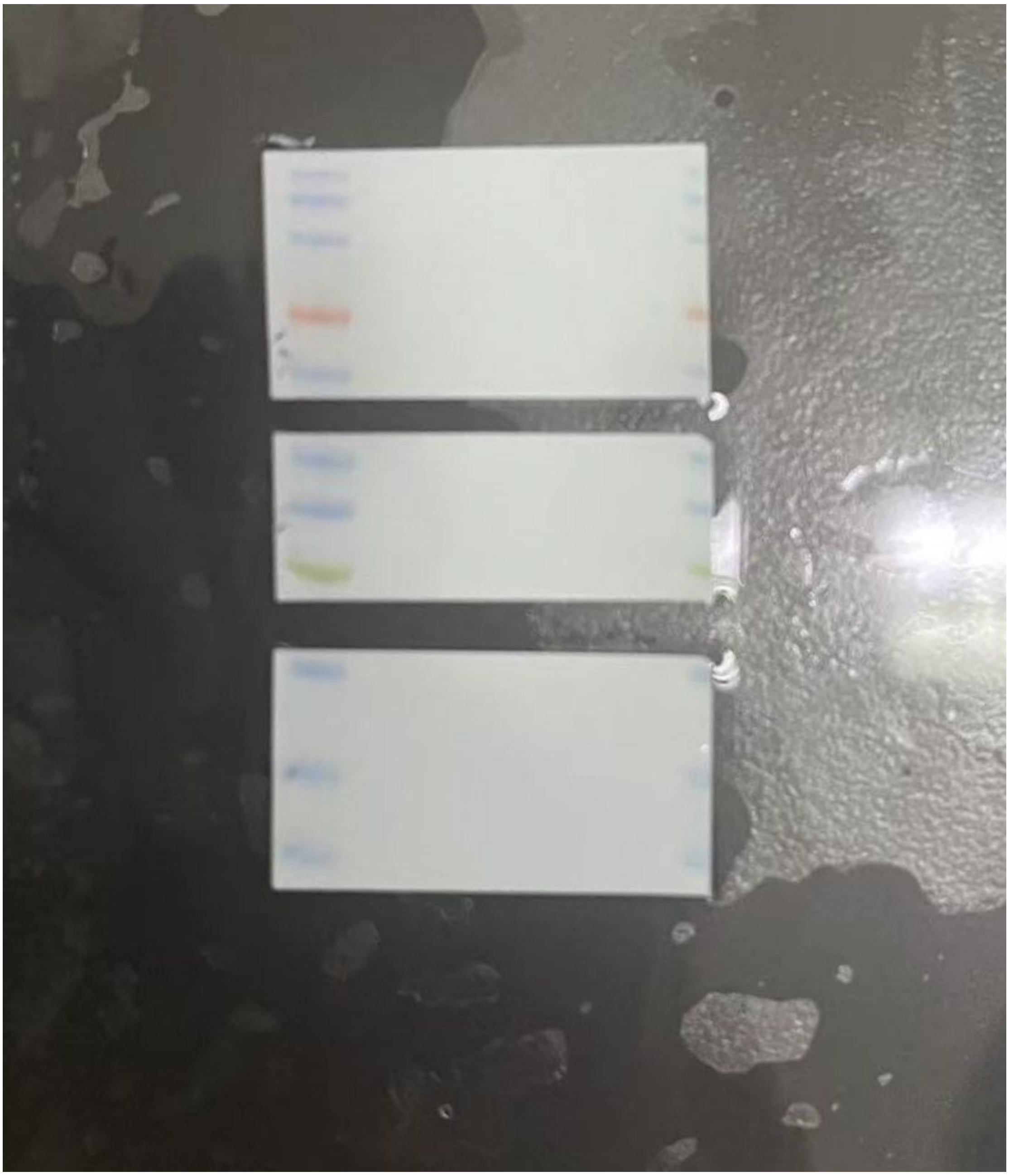

Fig5B-GAPDH&cGAS-2-全膜

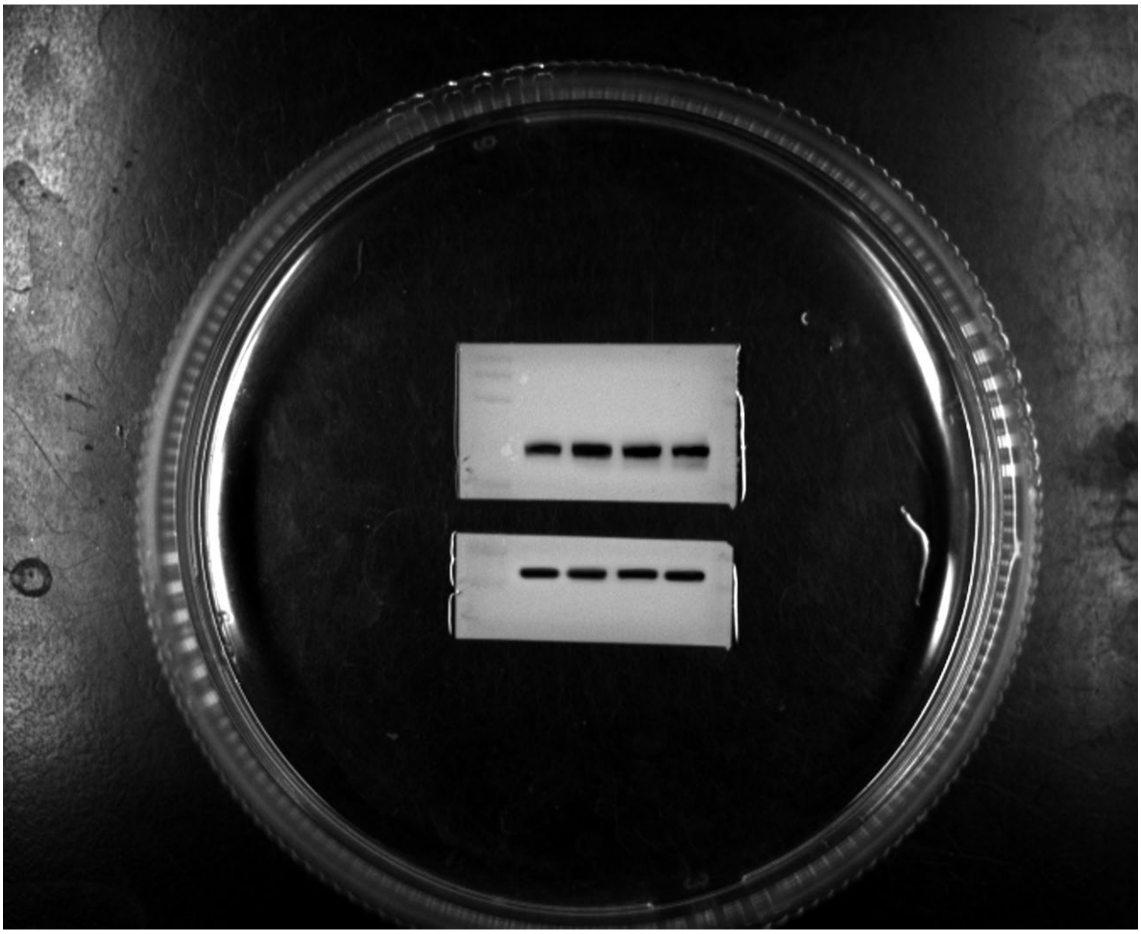

Fig5B-GAPDH&cGAS-2

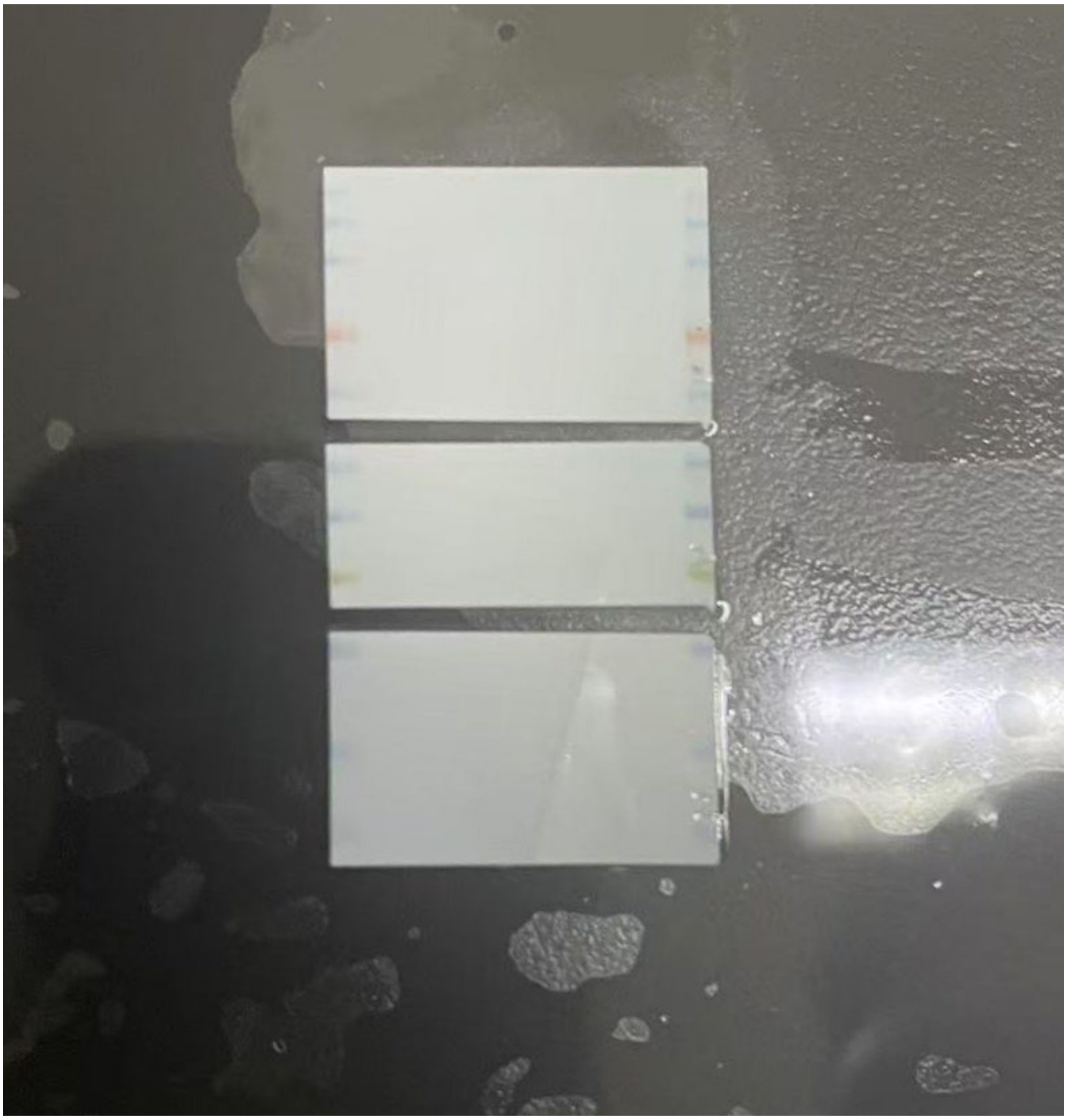

Fig5B-GAPDH&cGAS-3-全膜

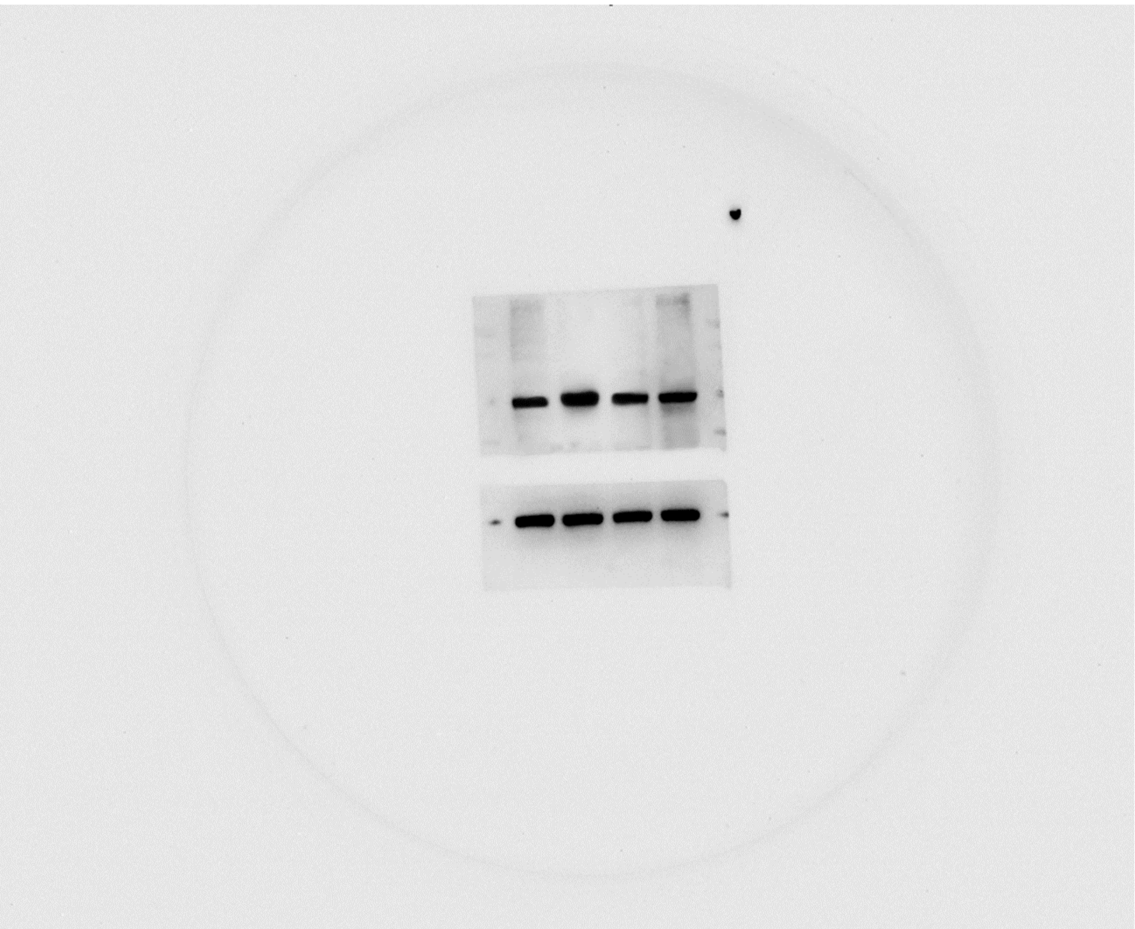

Fig5B-GAPDH&cGAS-3

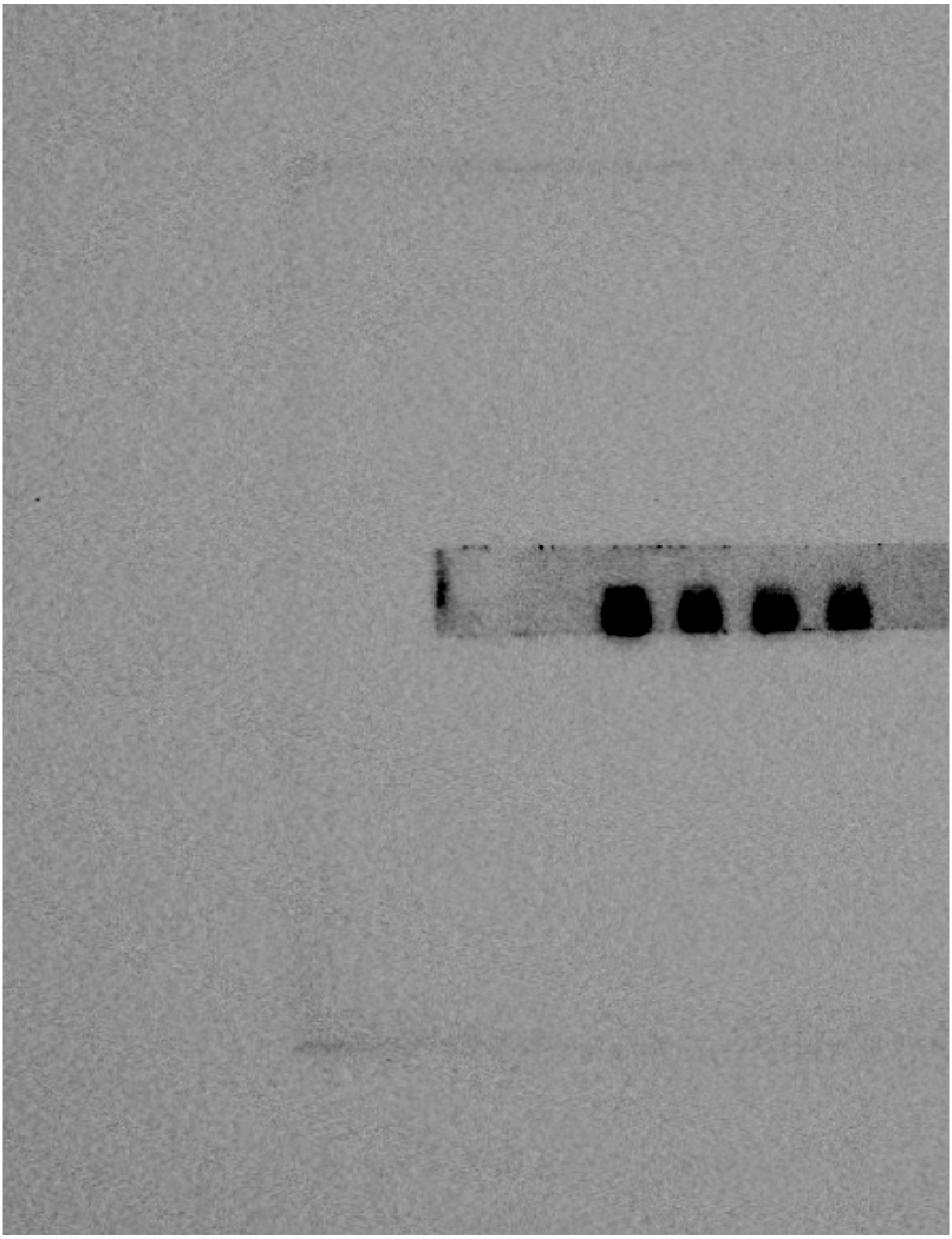

Fig5B-GAPDH-4

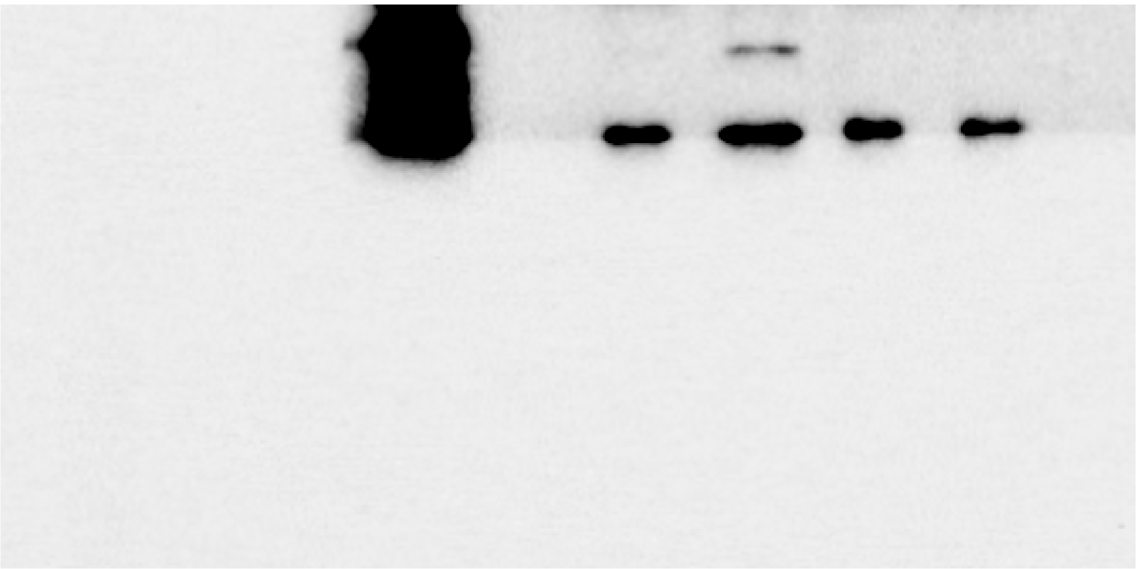

Fig5B-cGAS-4

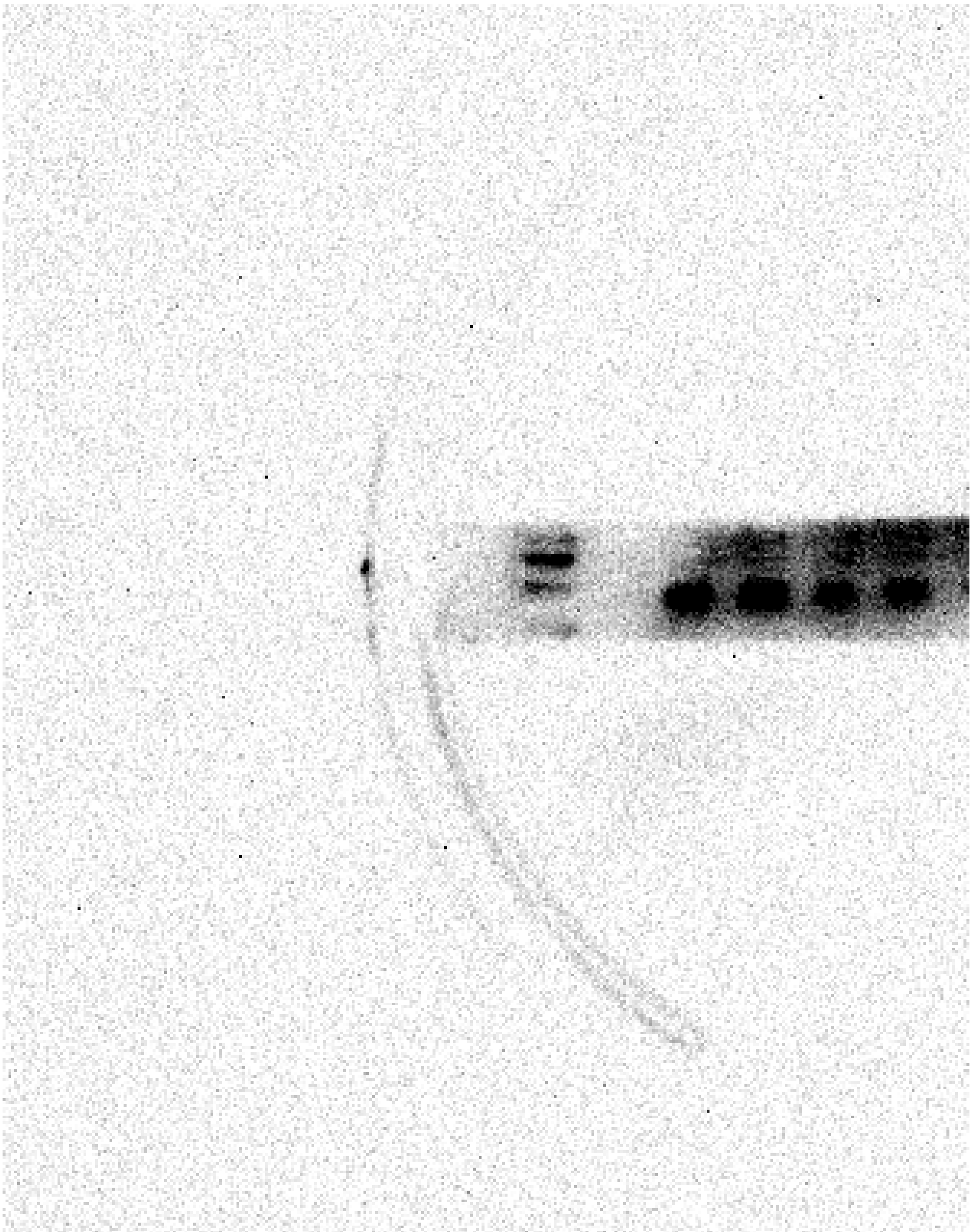

Fig5B-STING-4

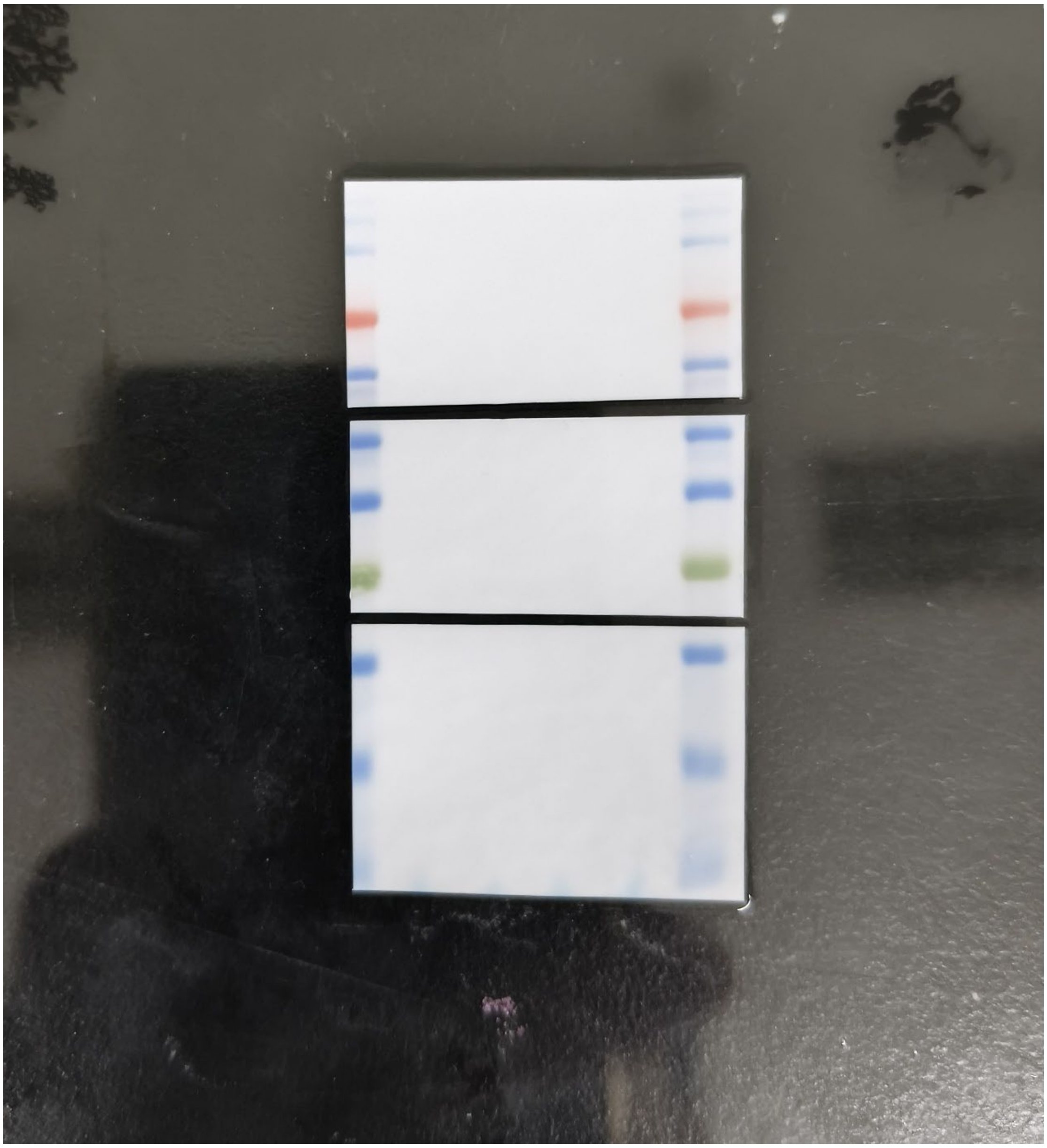

Fig5B-Tubin&STING-1-全膜

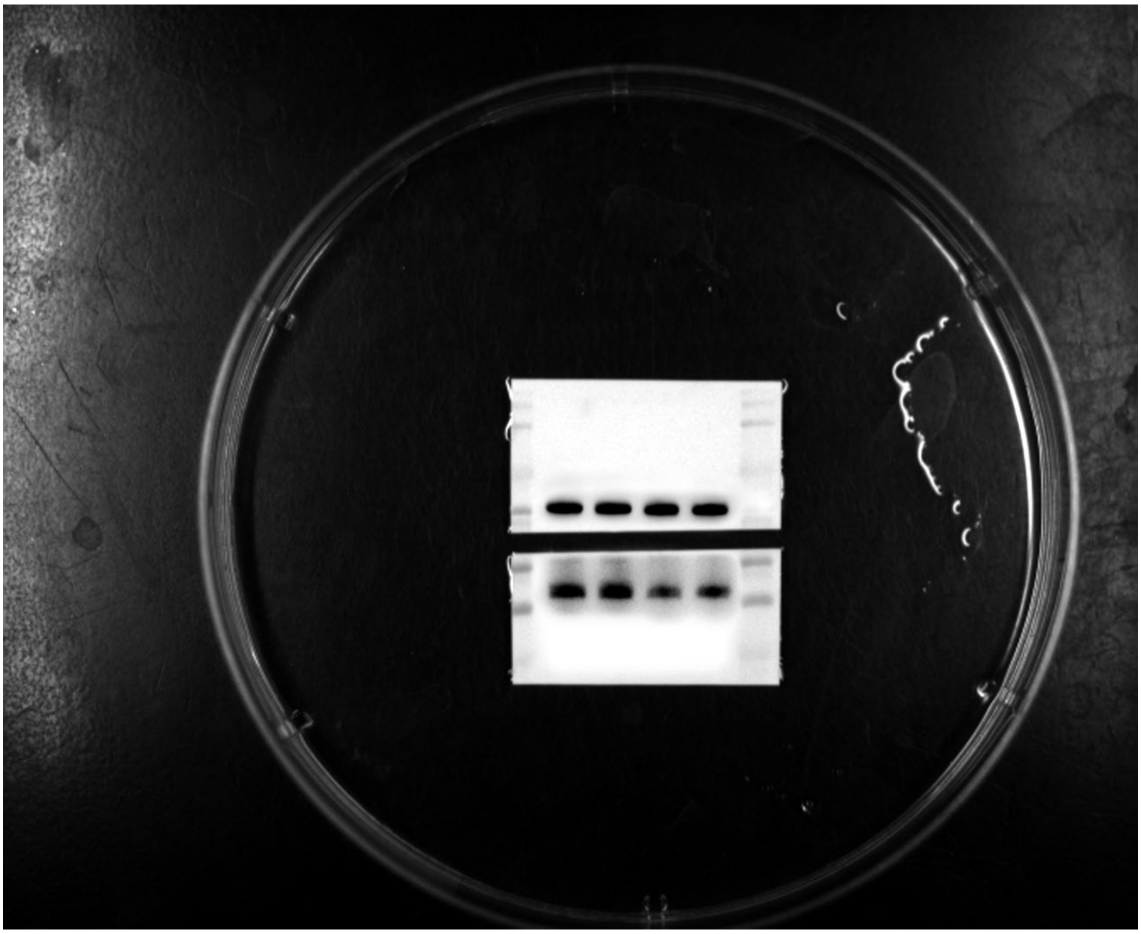

Fig5B-Tubin&STING-1

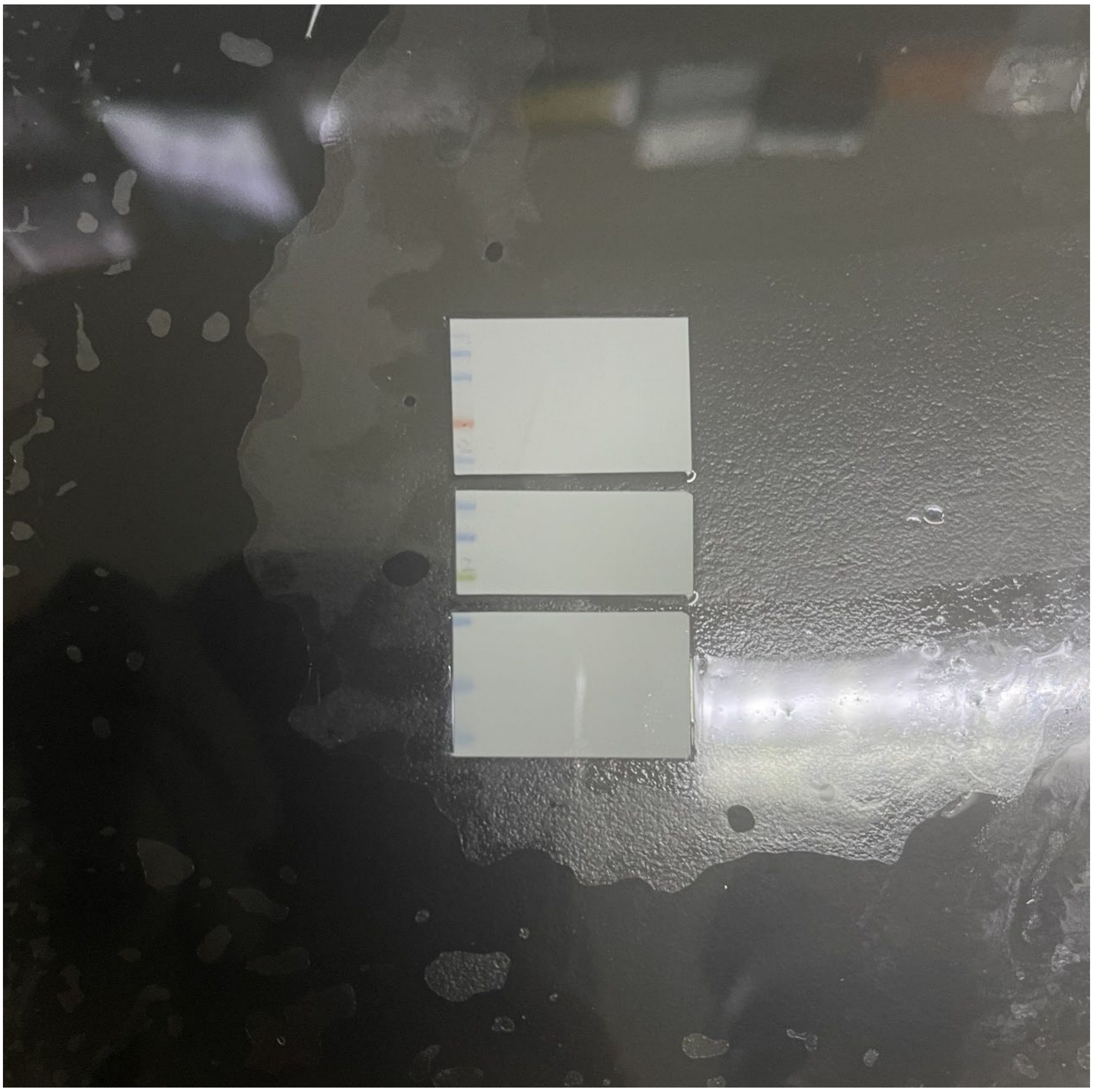

Fig5B-Tubin&STING-2-全膜

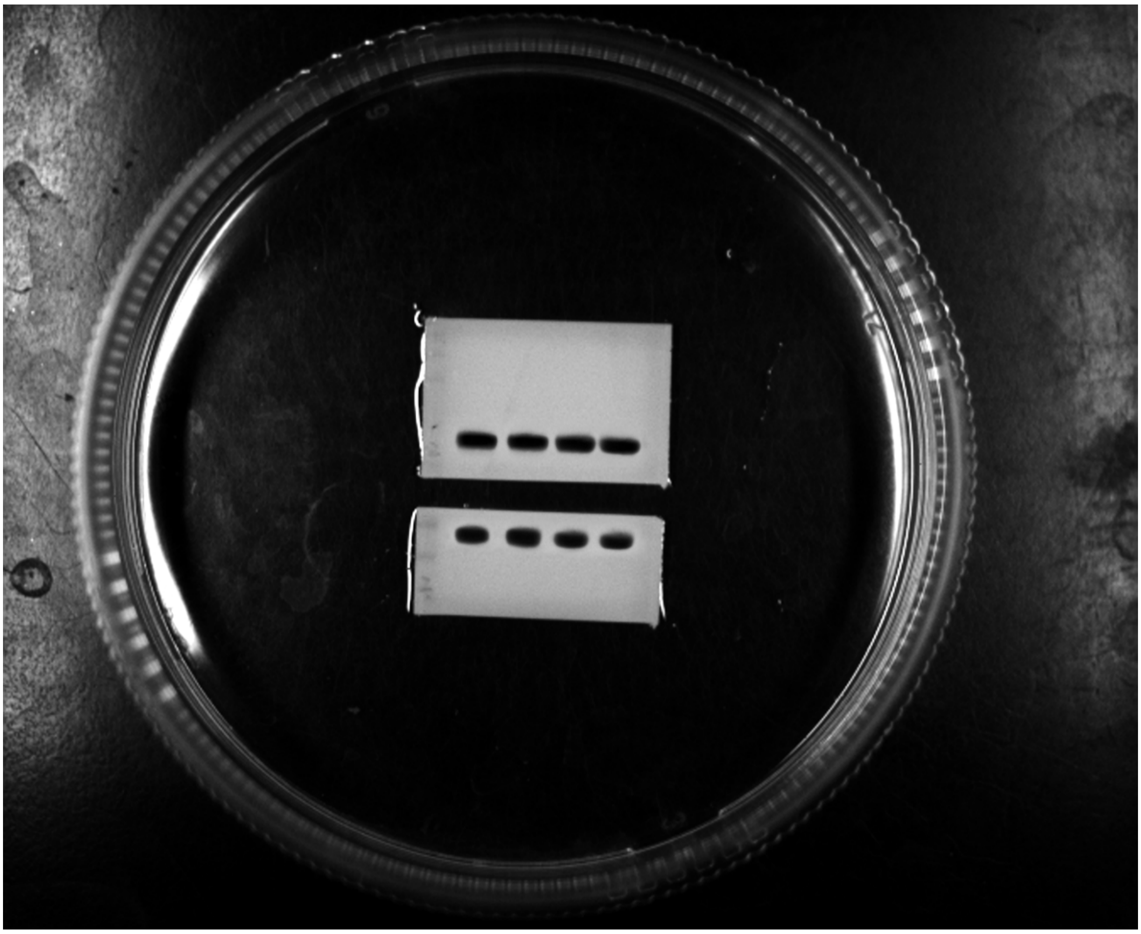

Fig5B-Tubin&STING-2

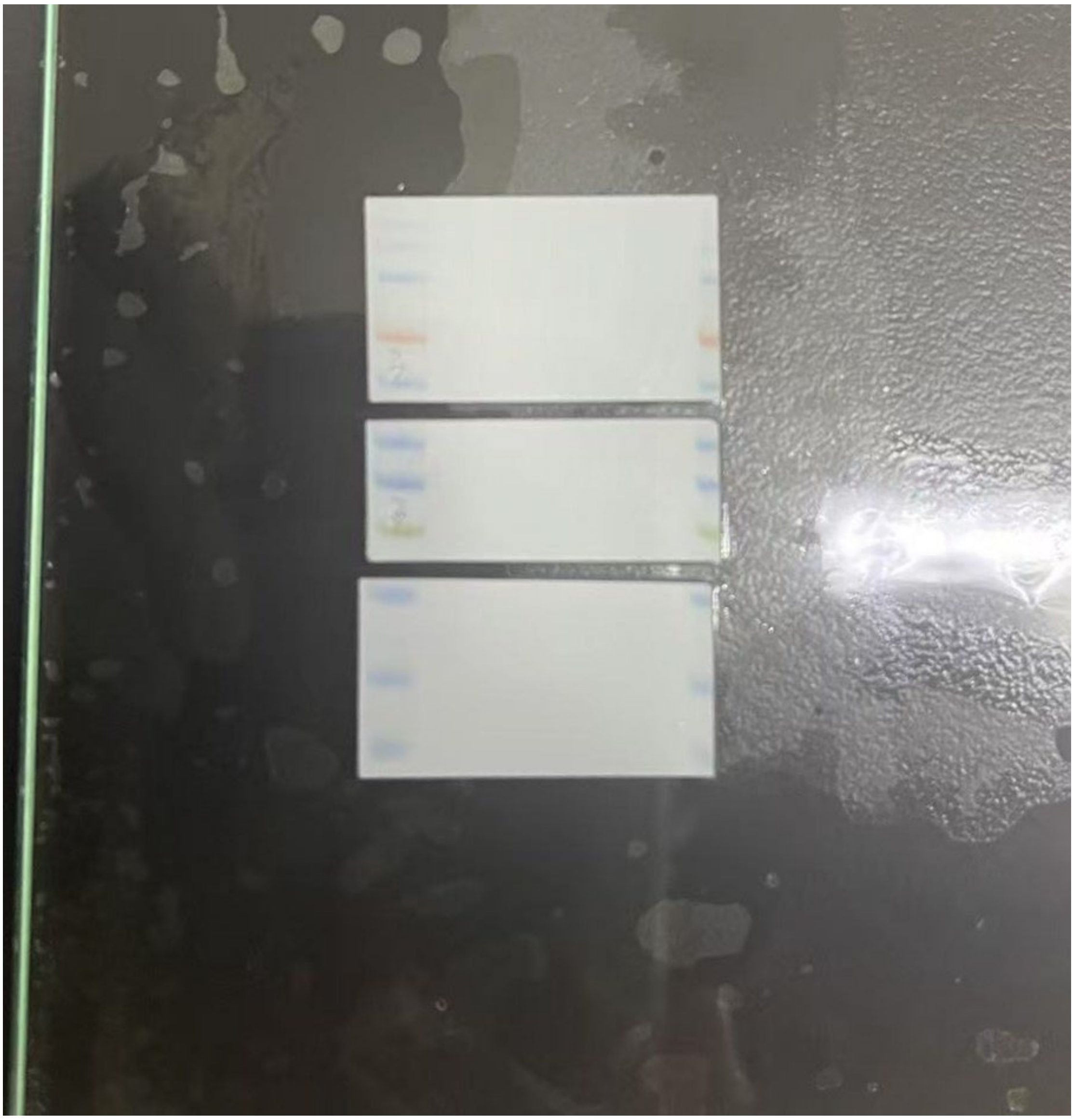

Fig5B-Tubin&STING-3-全膜

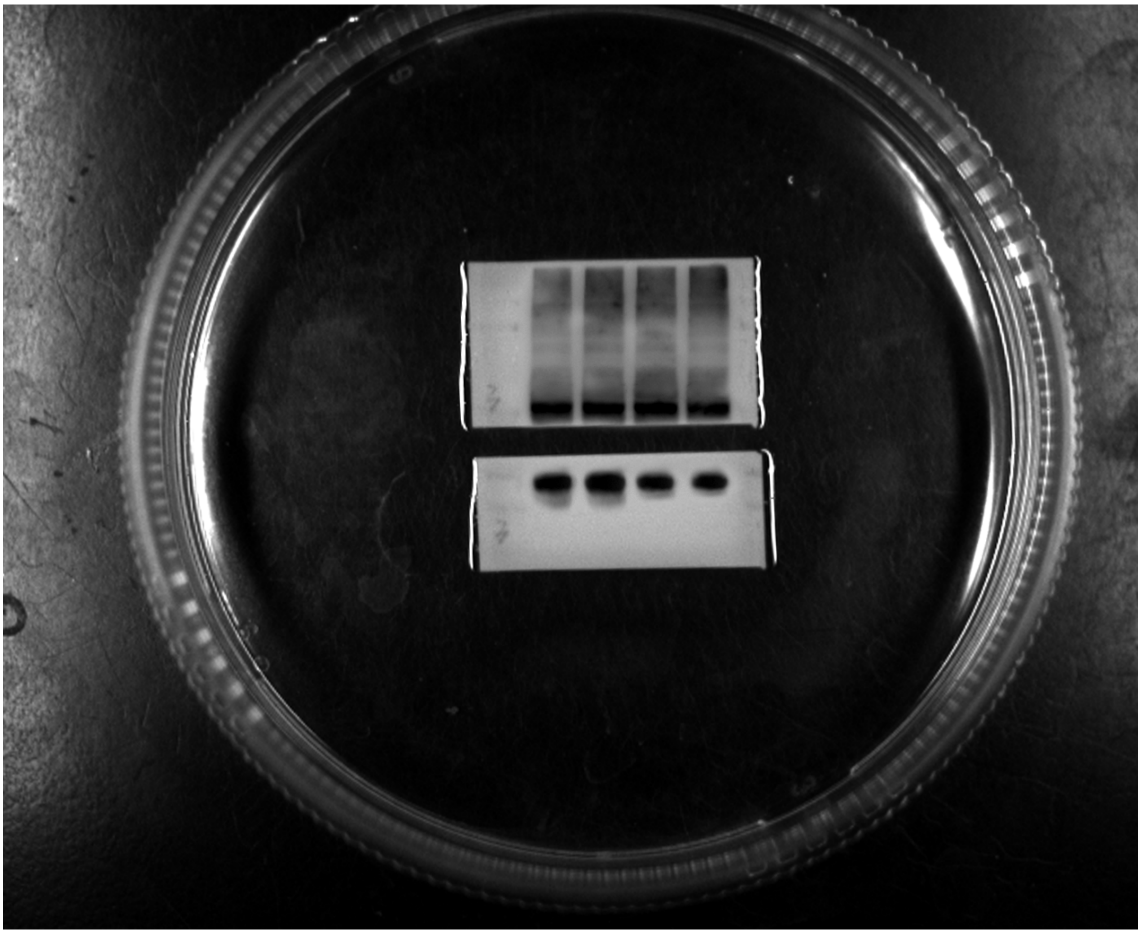

Fig5B-Tubin&STING-3

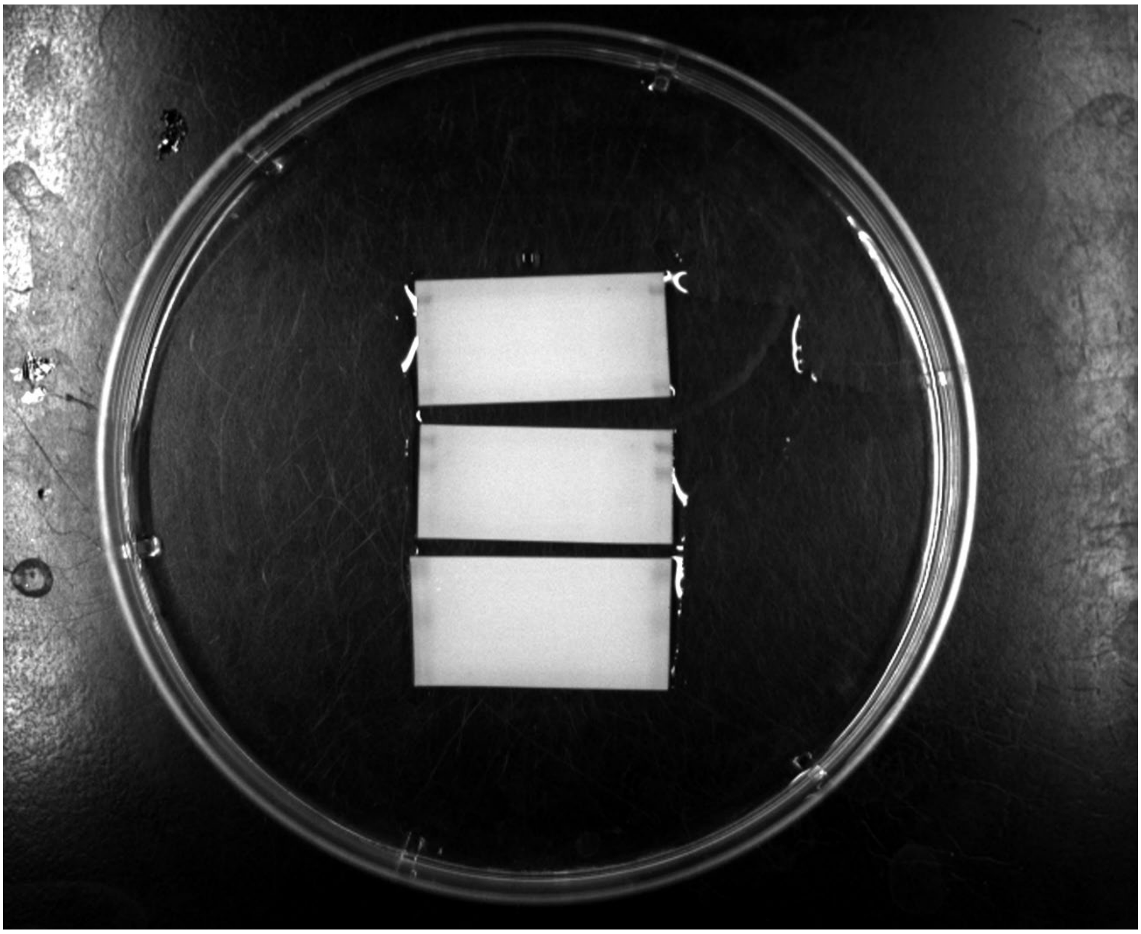

Fig5D-GAPDH&p62&LC3-1-全膜

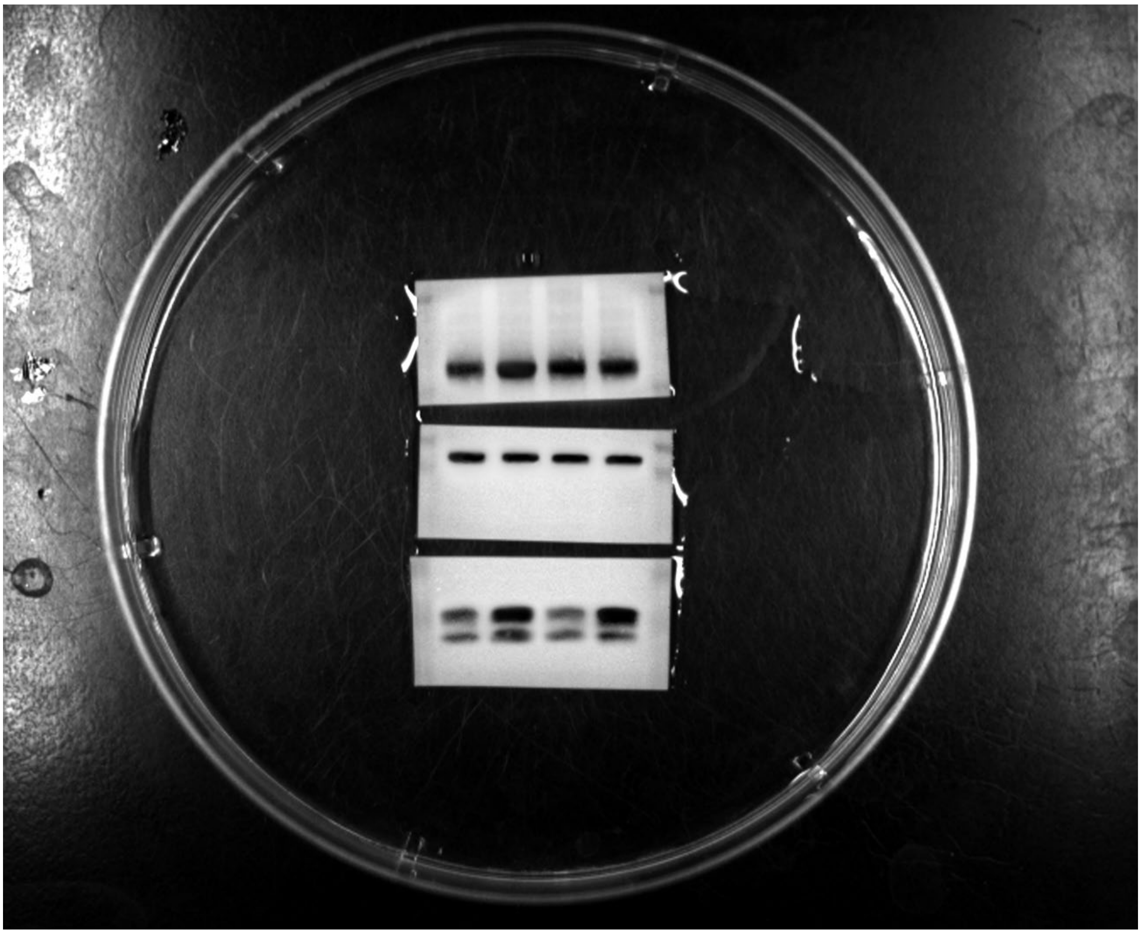

Fig5D-GAPDH&p62&LC3-1

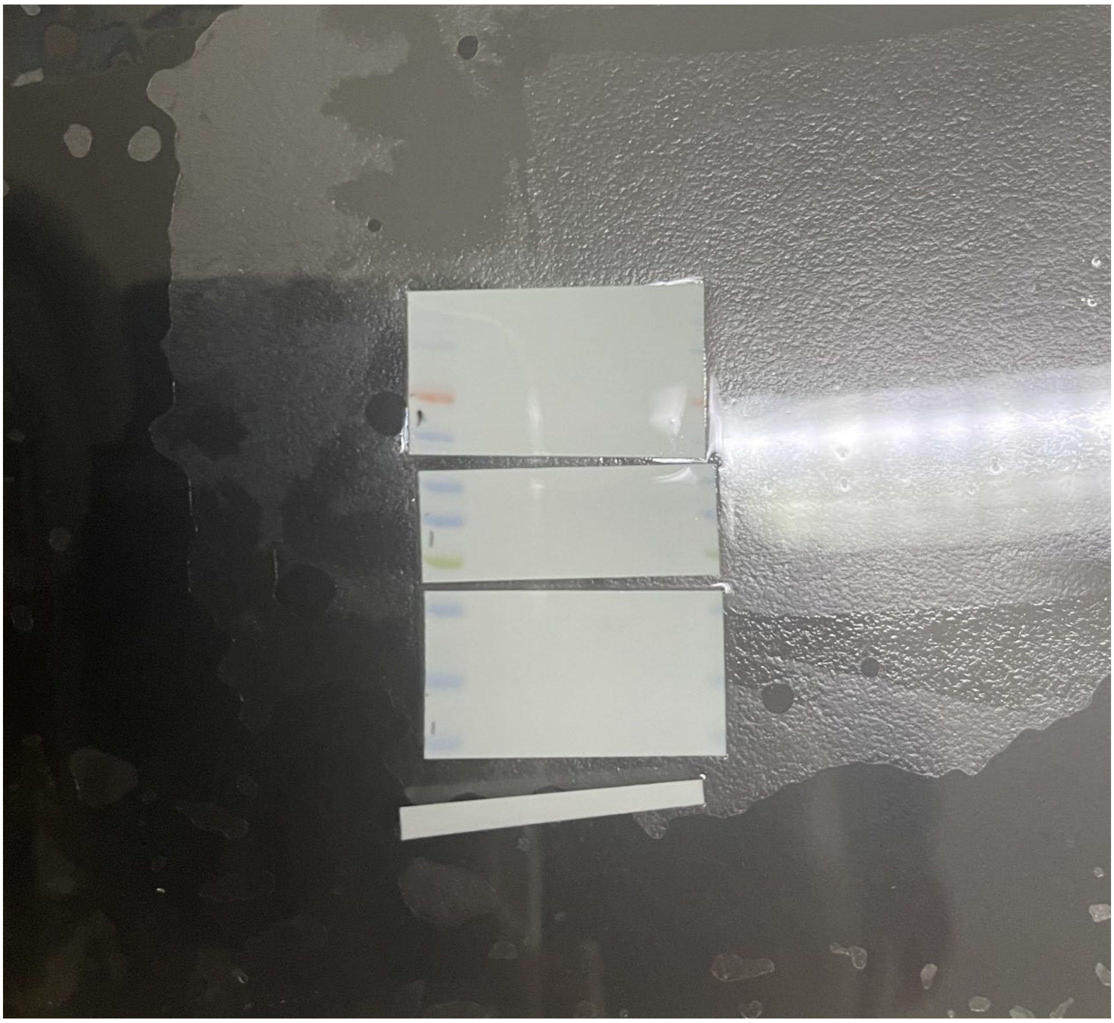

Fig5D-GAPDH&p62&LC3-2-全膜

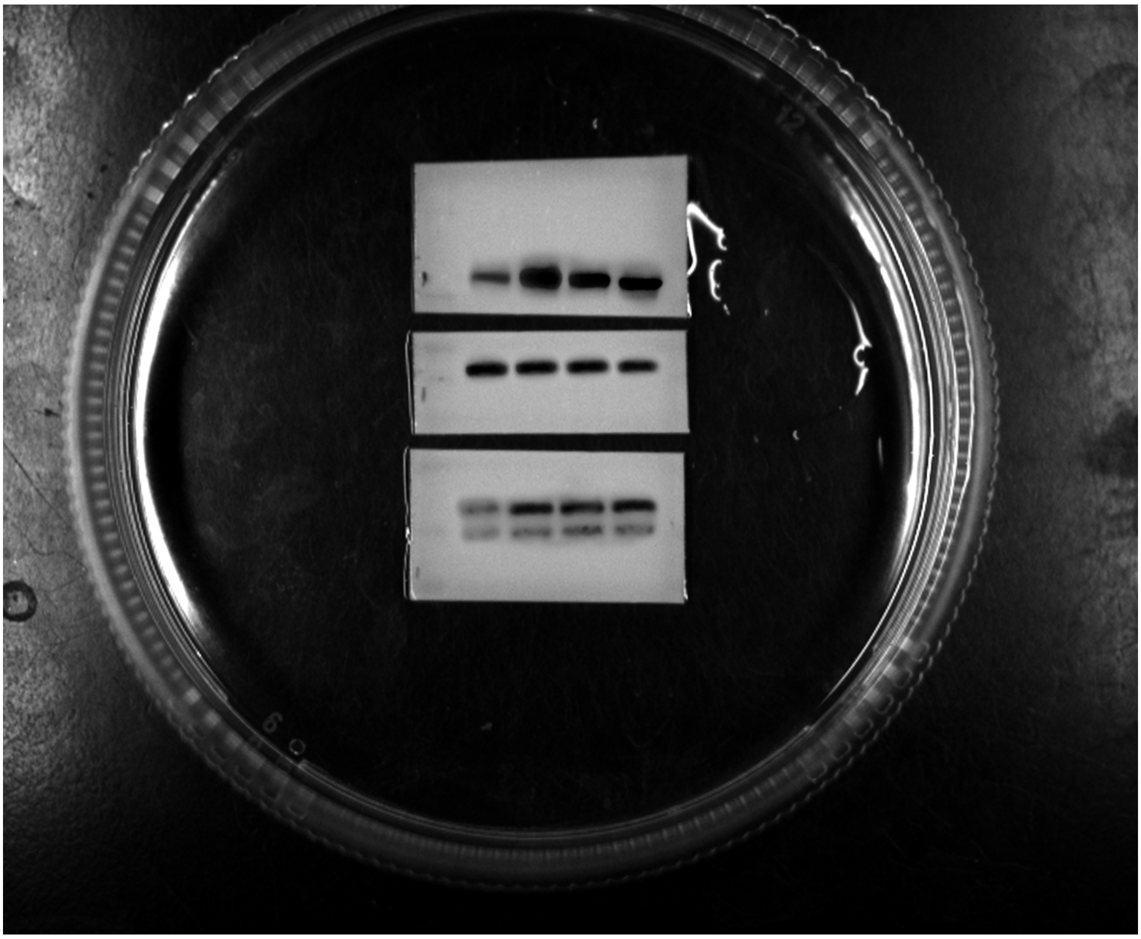

Fig5D-GAPDH&p62&LC3-2

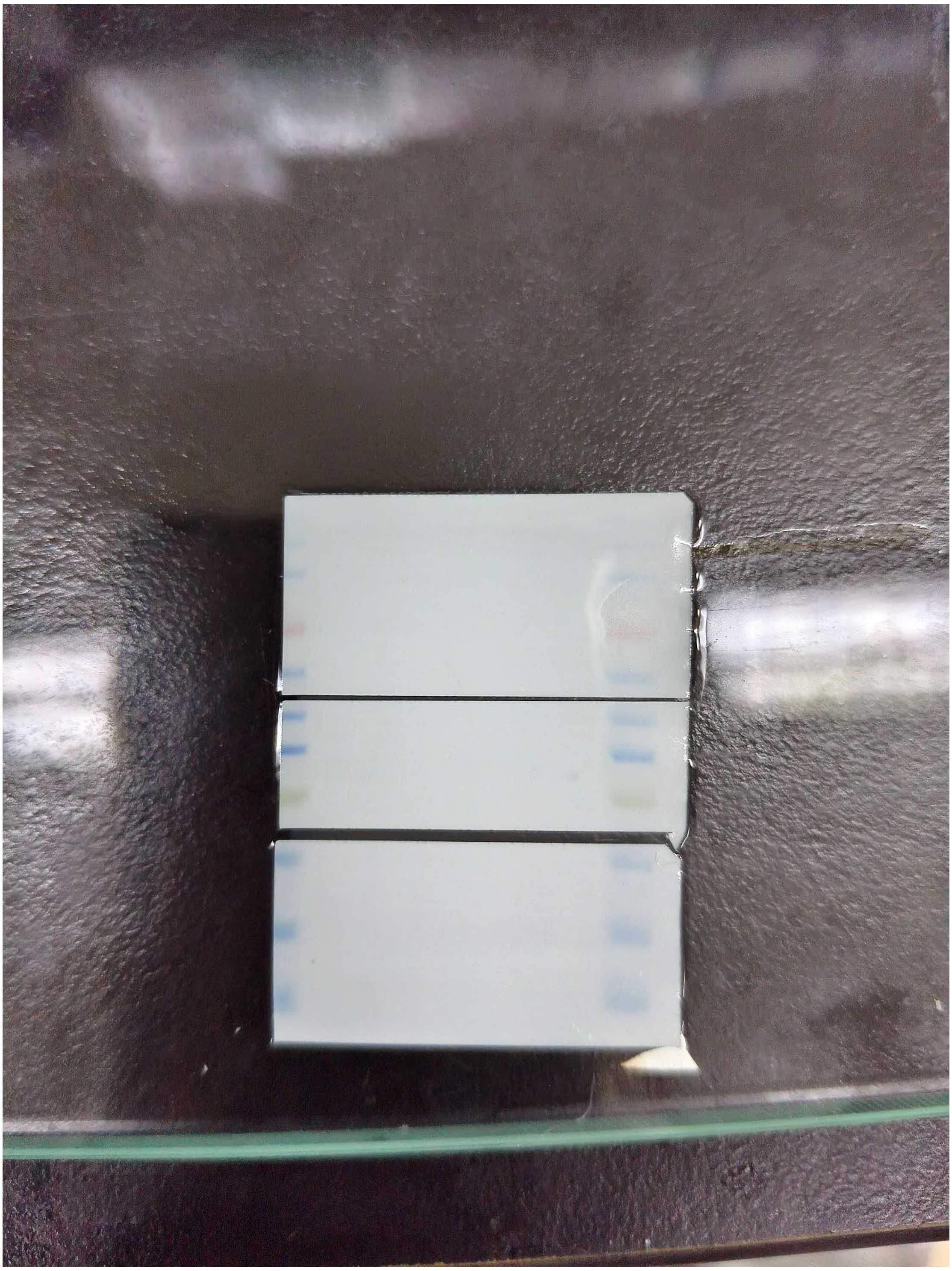

Fig5D-GAPDH&p62&LC3-3-全膜

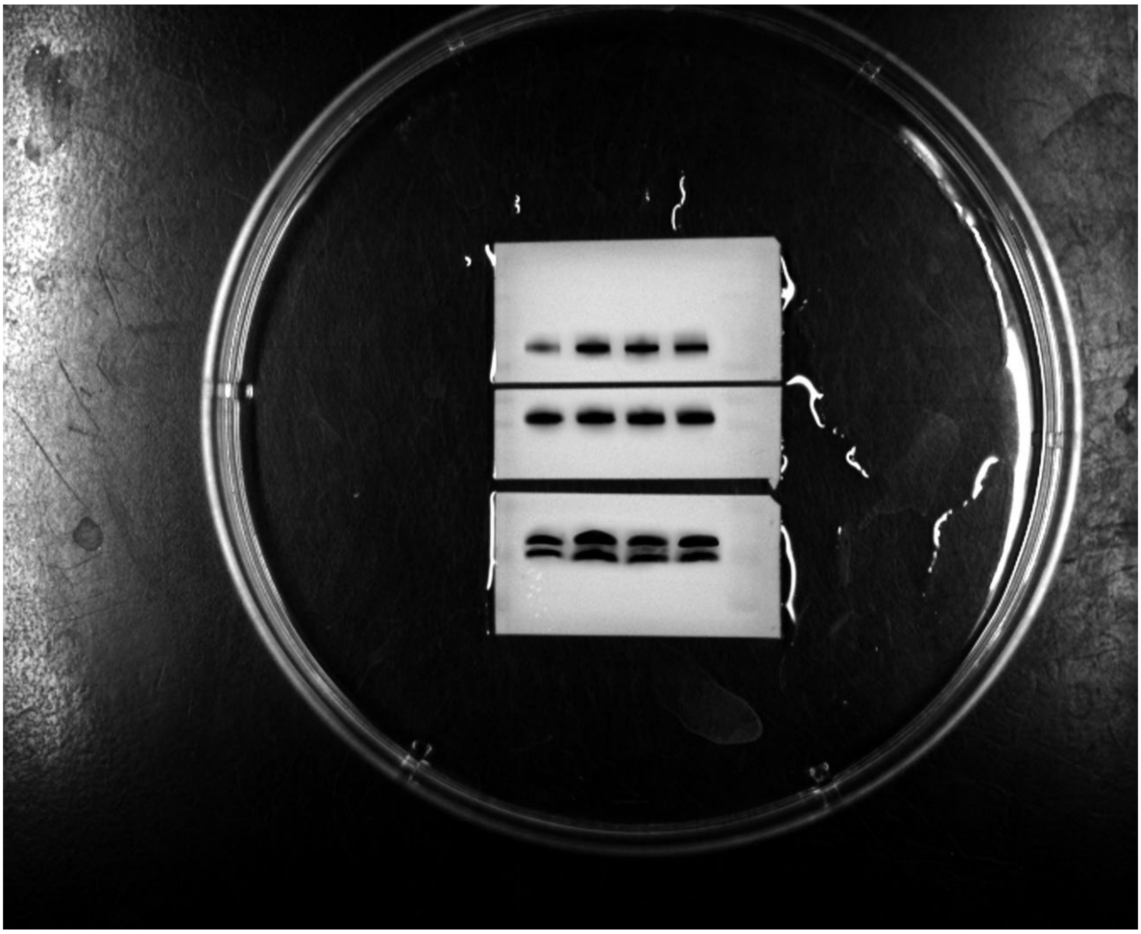

Fig5D-GAPDH&p62&LC3-3

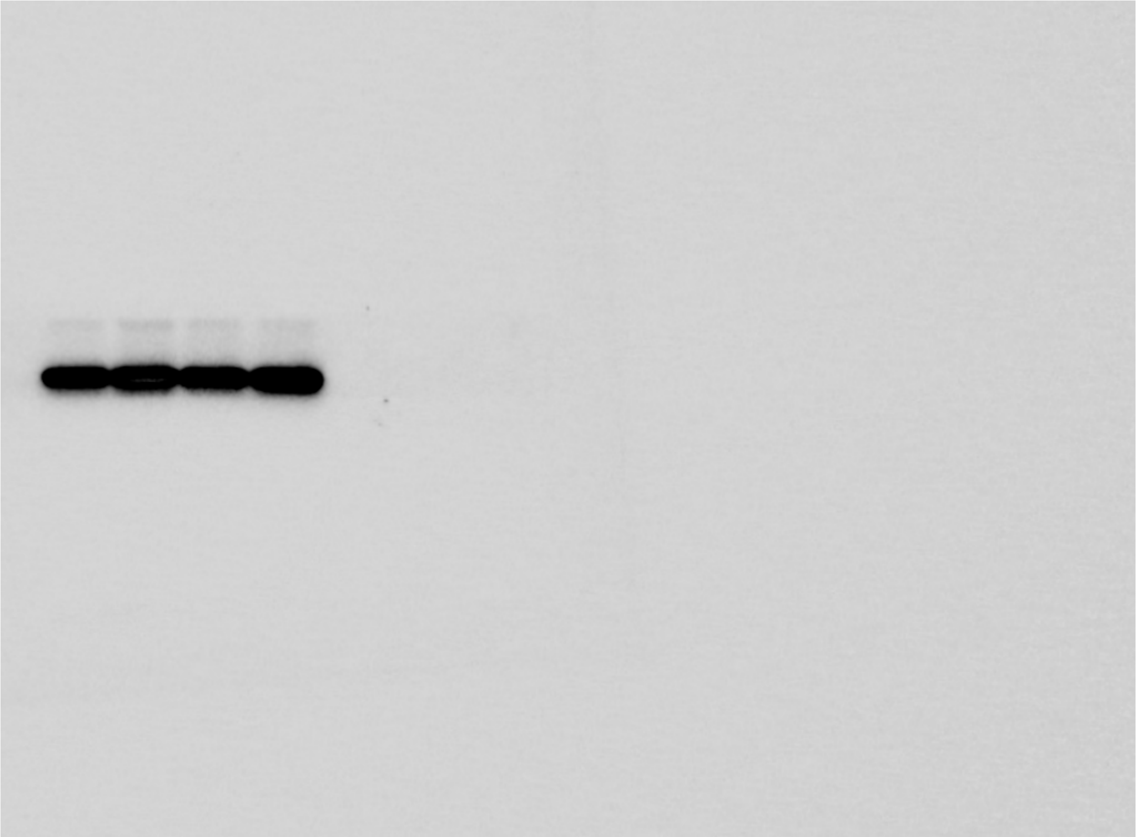

Fig5D-GAPDH-4

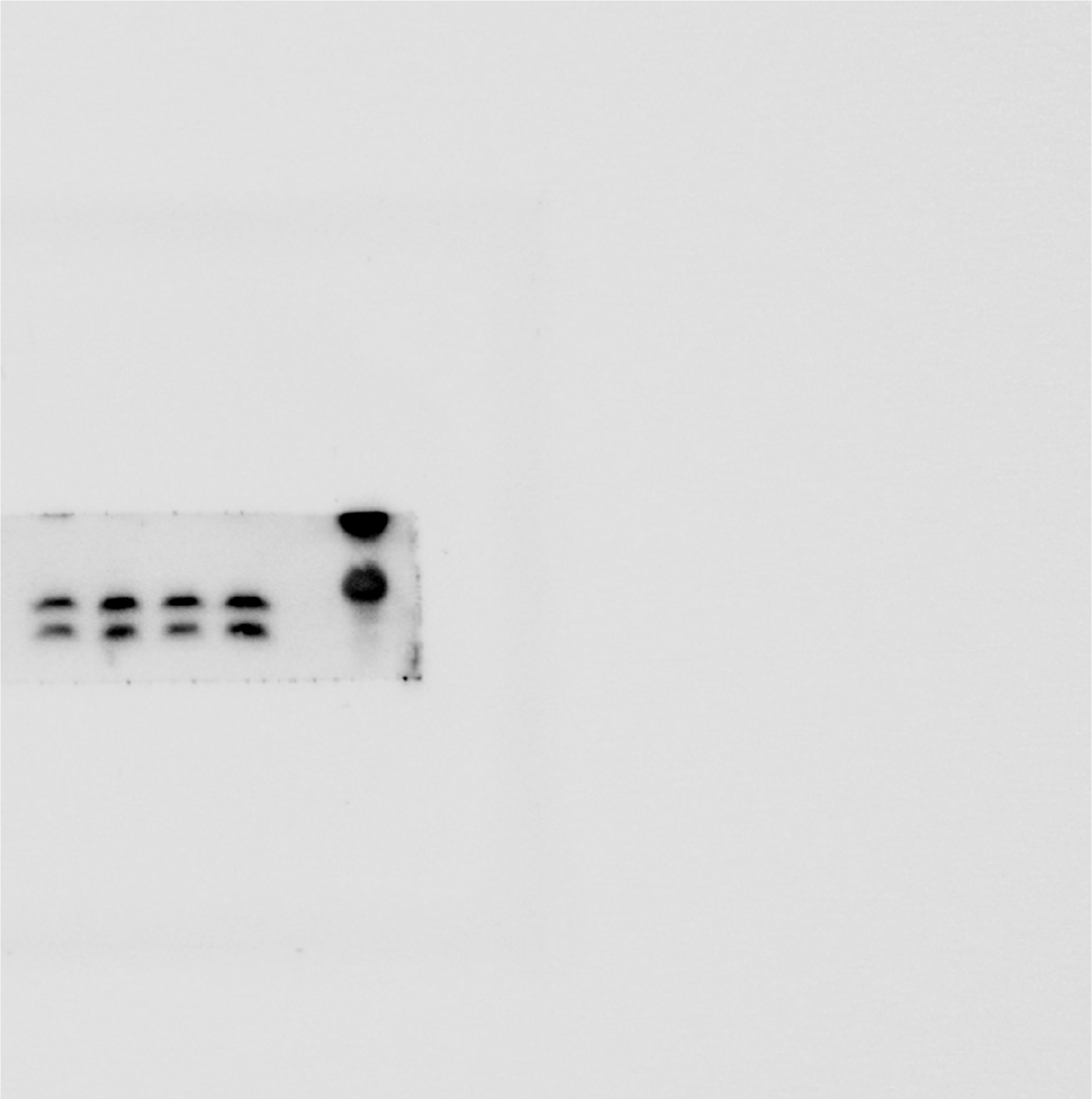

Fig5D-LC3-4

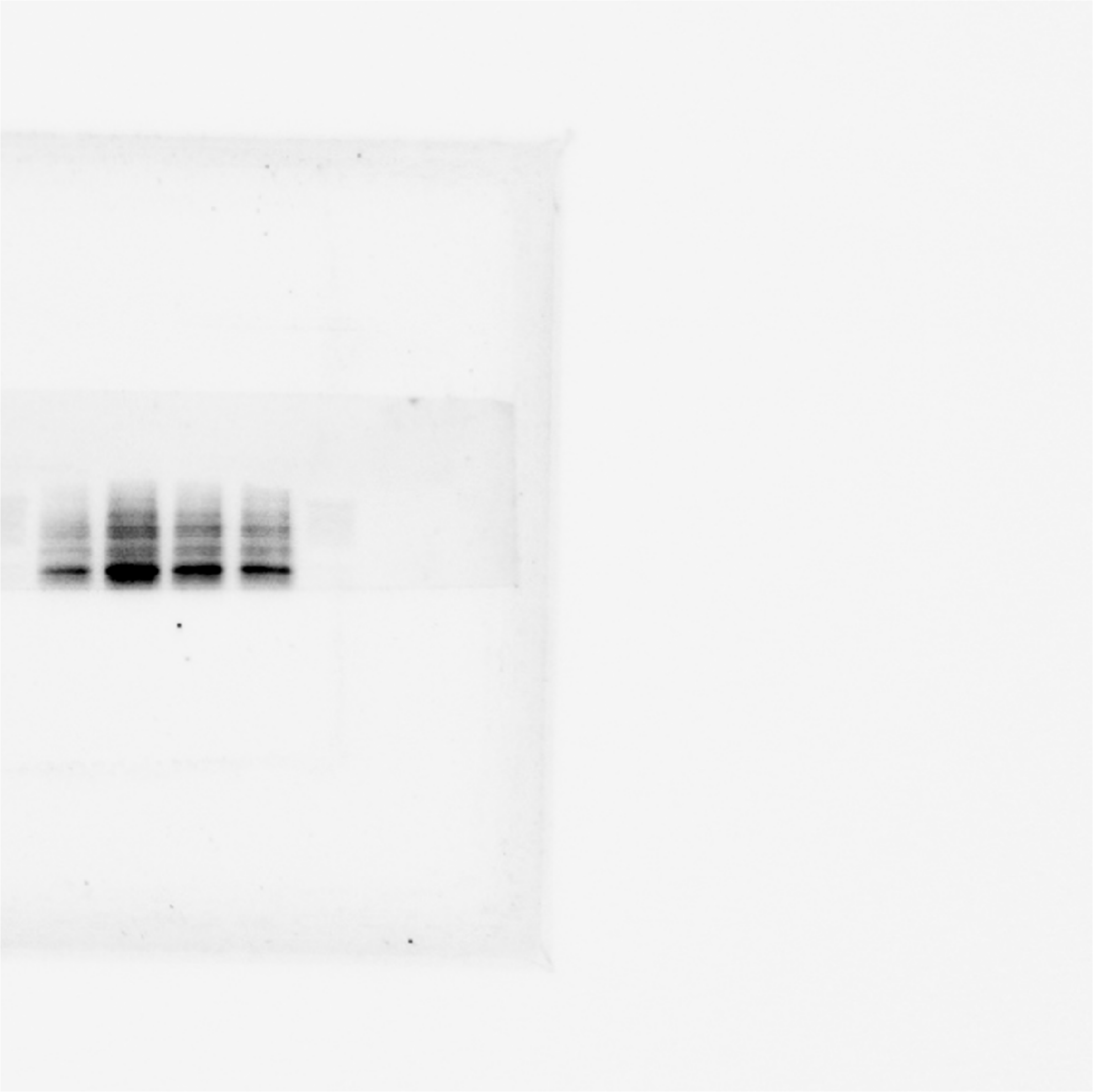

Fig5D-p62-4

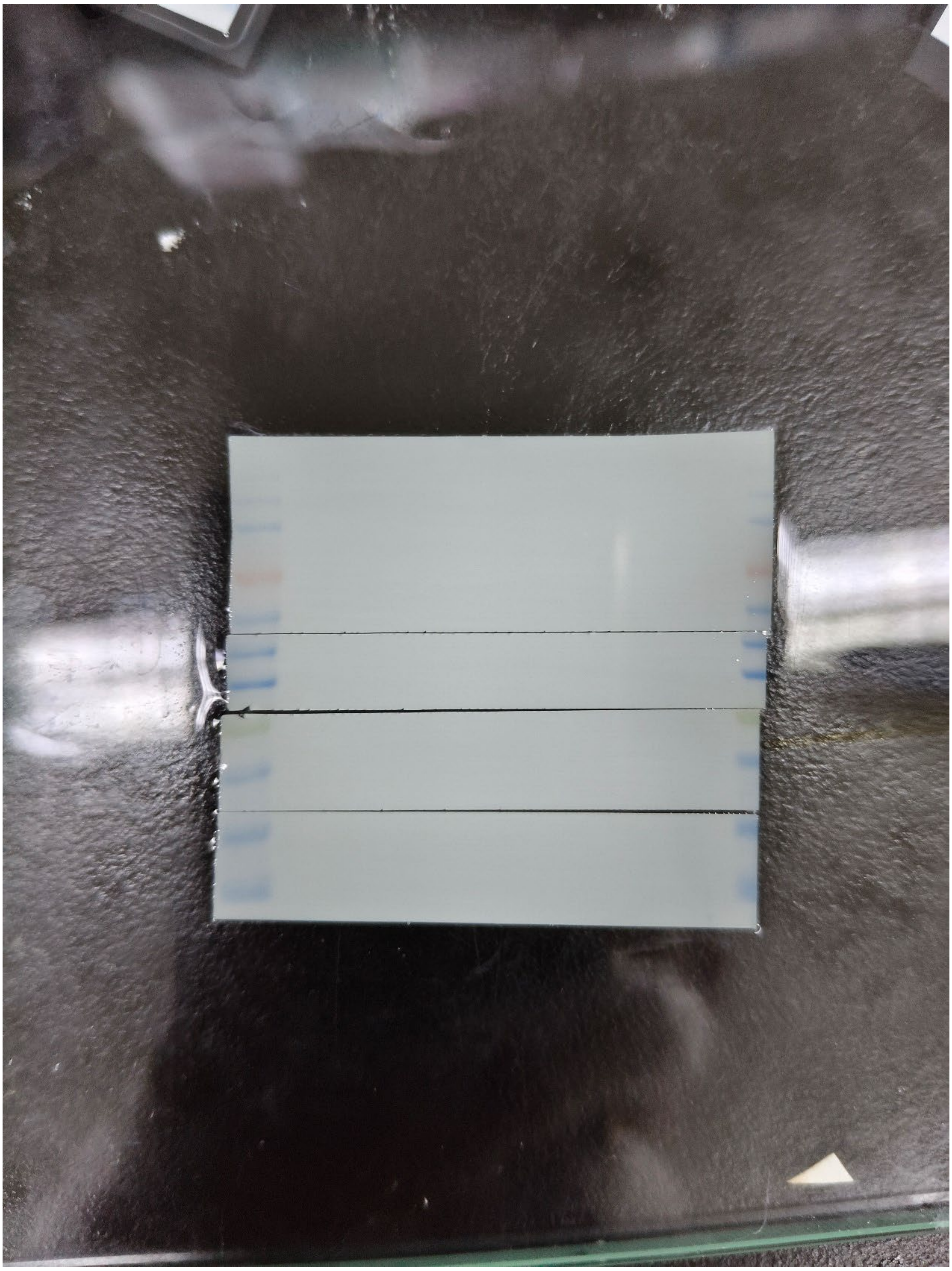

Fig6B-GAPDH&Cav1-1-全膜

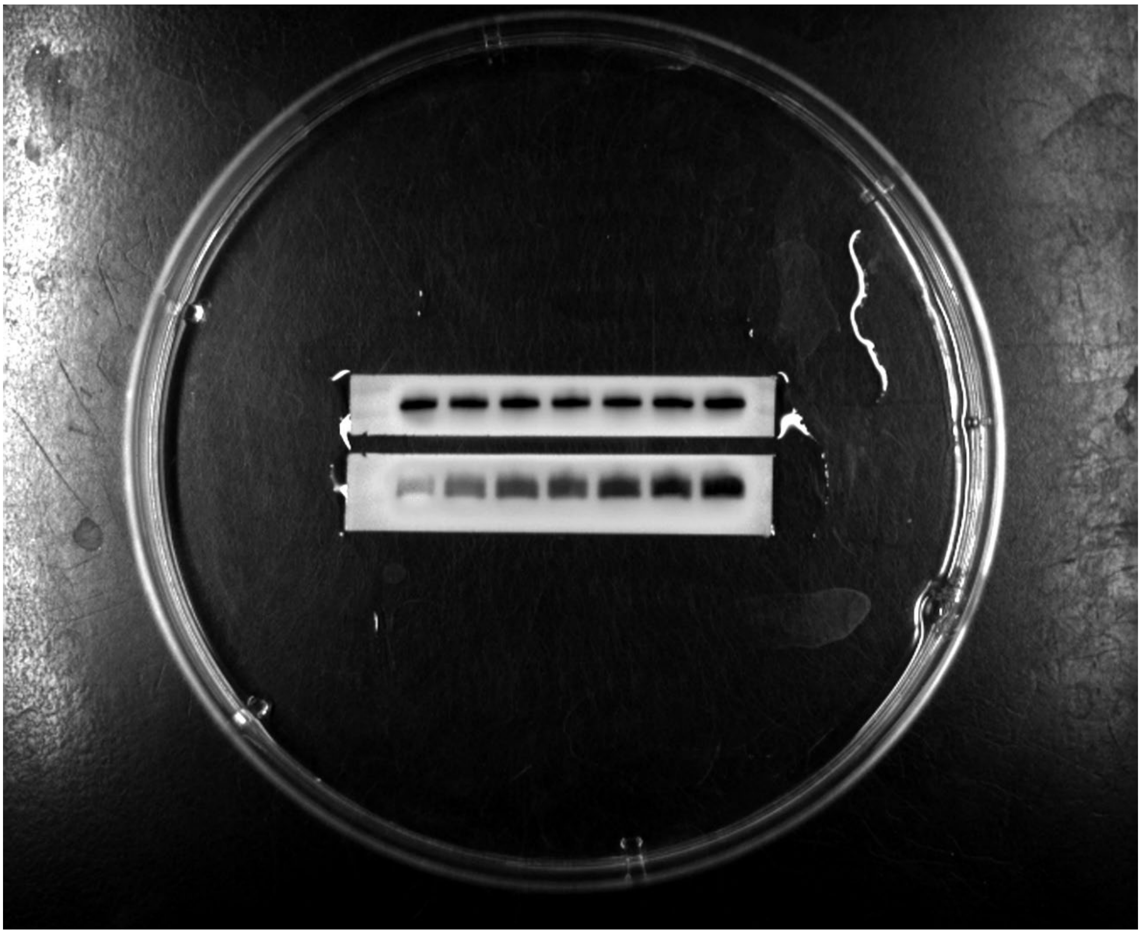

Fig6B-GAPDH&Cav1-1

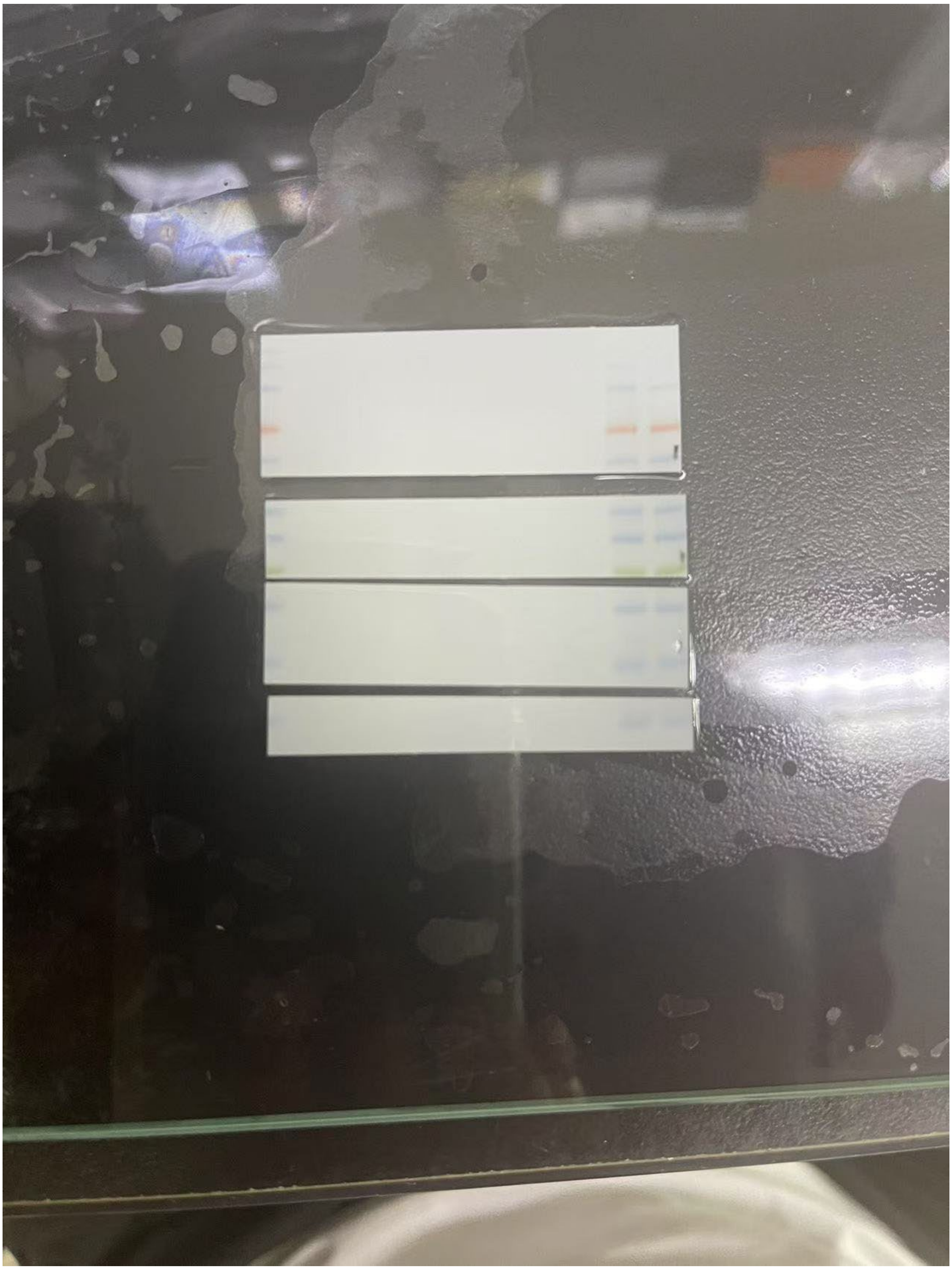

Fig6B-GAPDH&Cav1-2-全膜

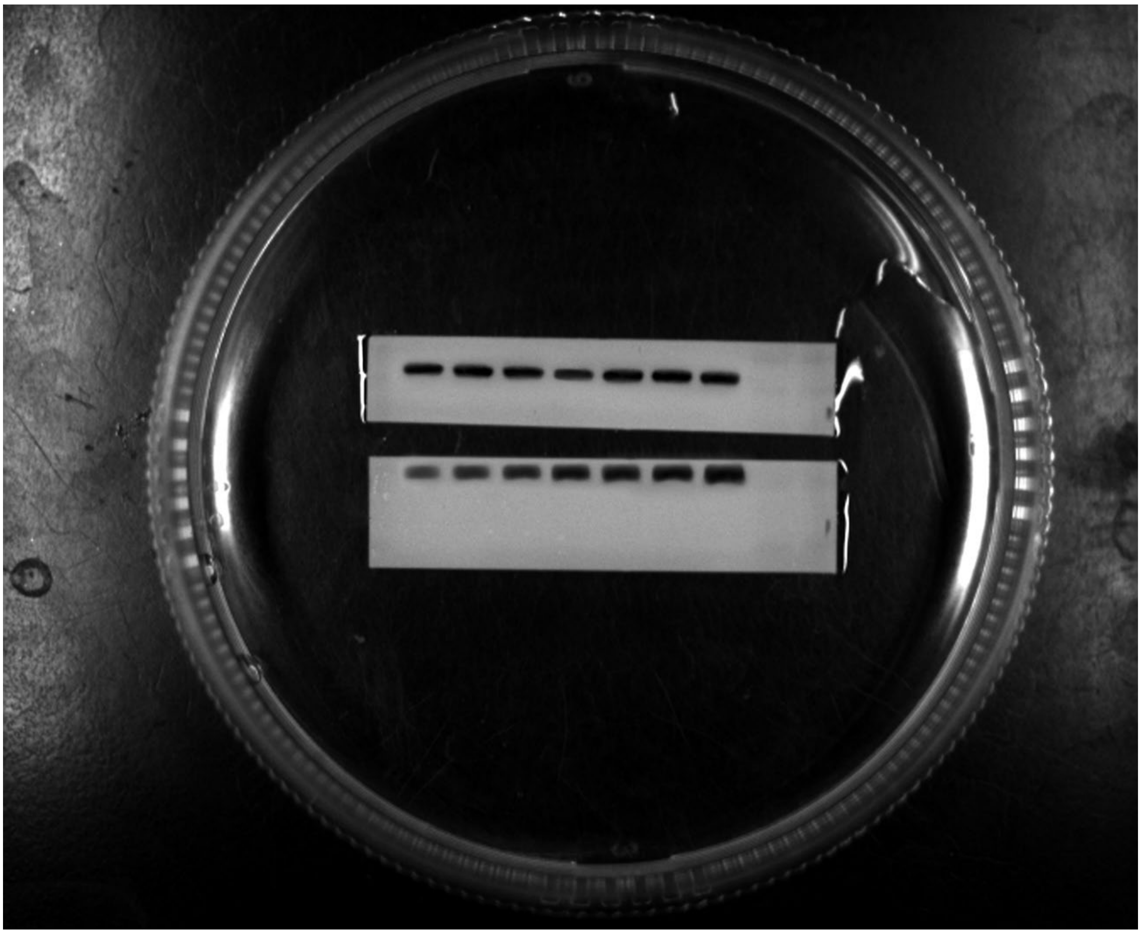

Fig6B-GAPDH&Cav1-2

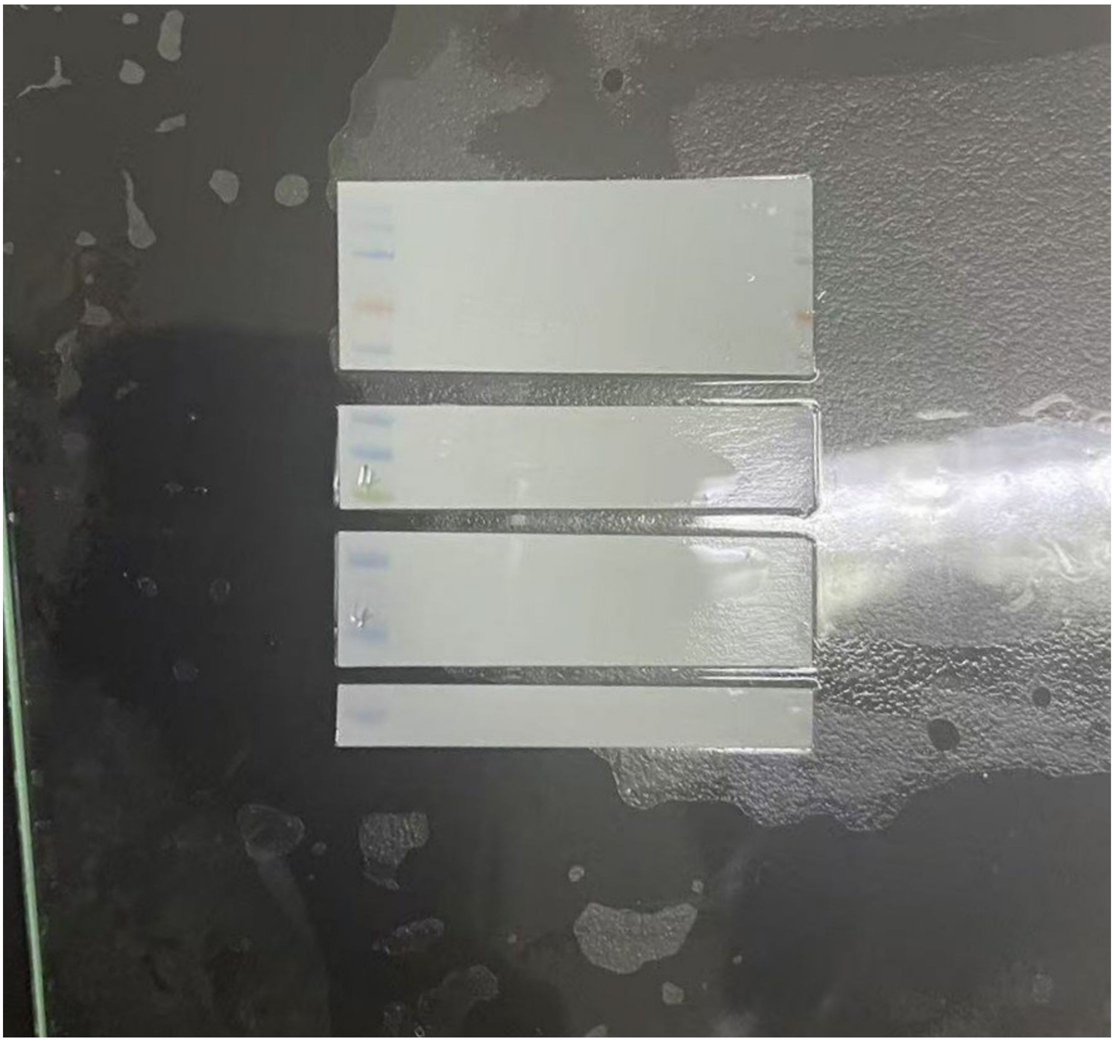

Fig6B-GAPDH&Cav1-3-全膜

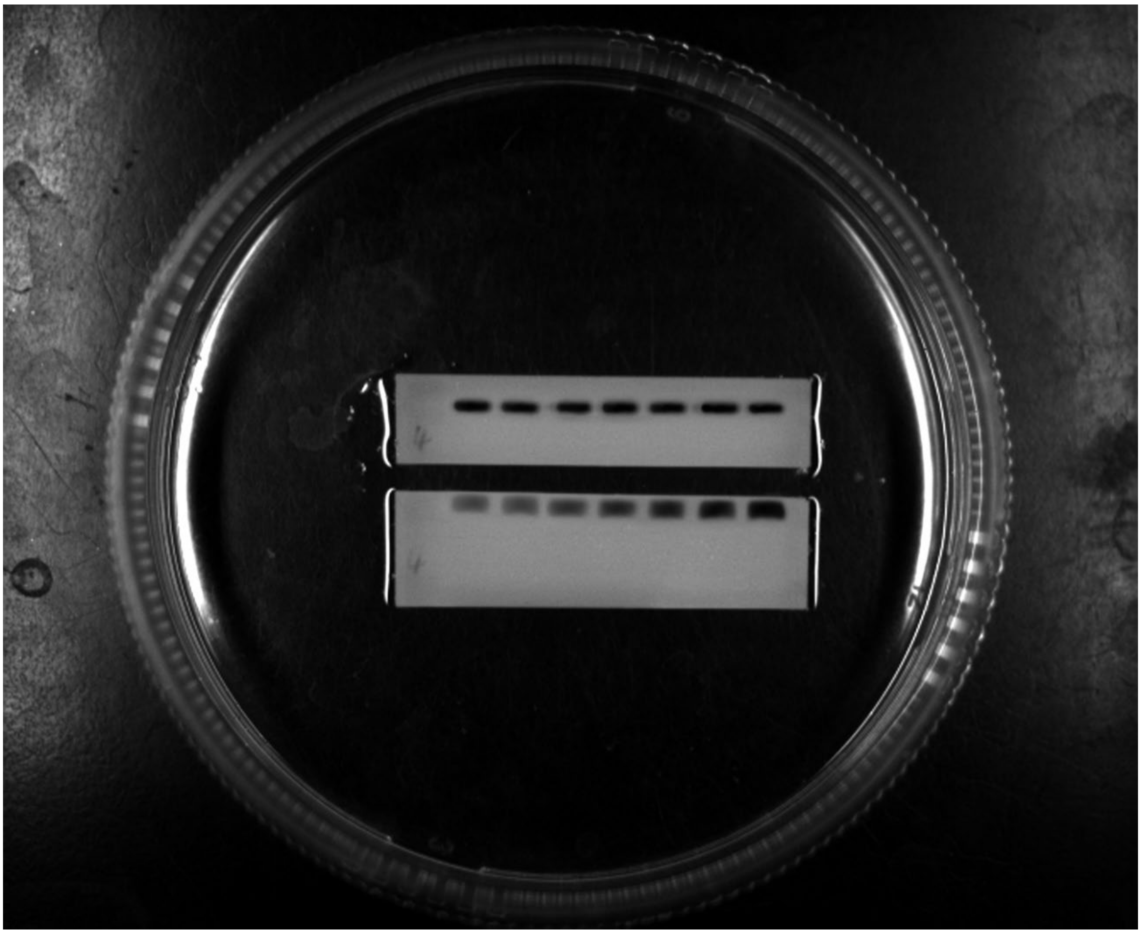

Fig6B-GAPDH&Cav1-3

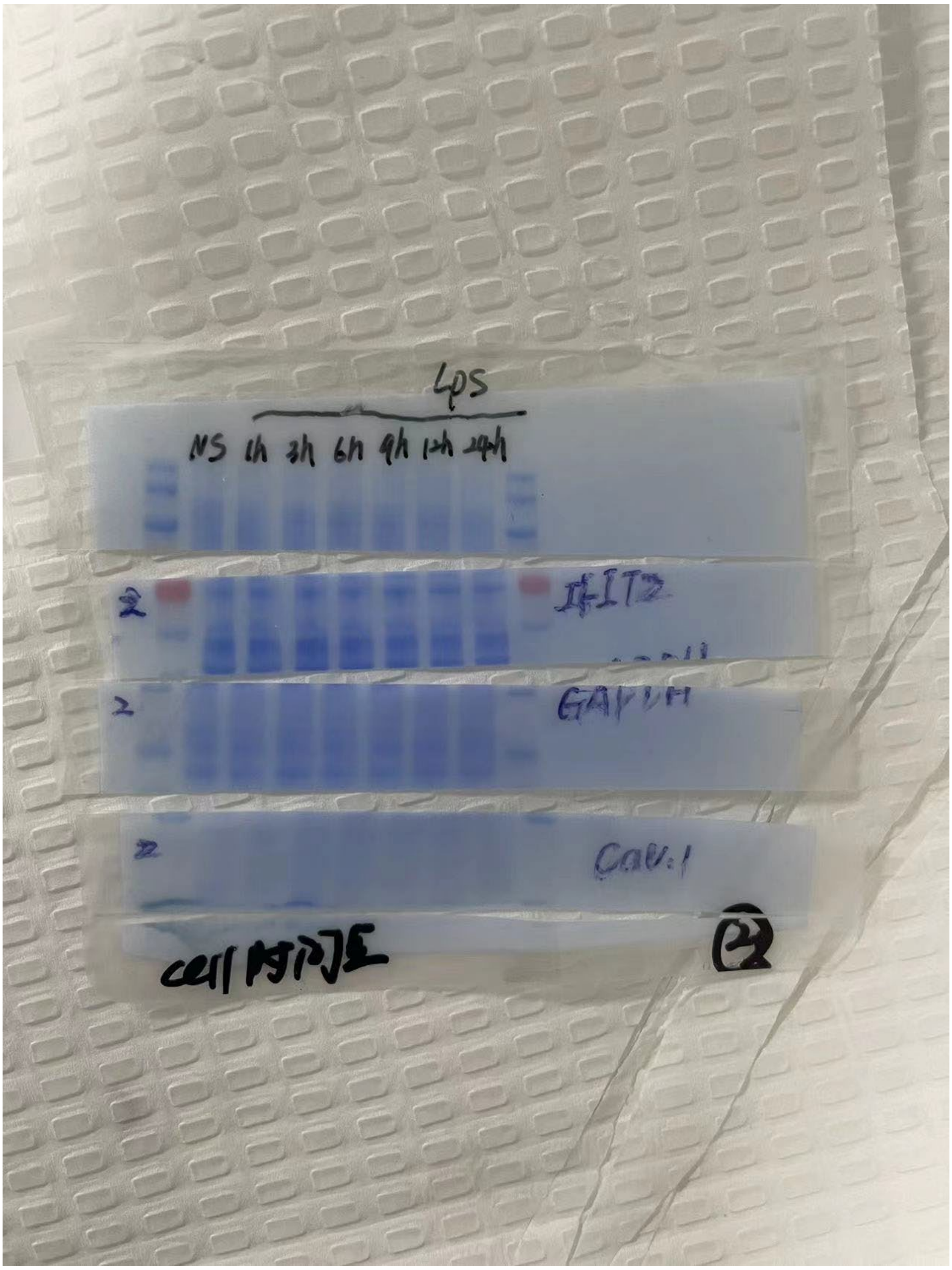

Fig6B-GAPDH&Cav1-4-全膜

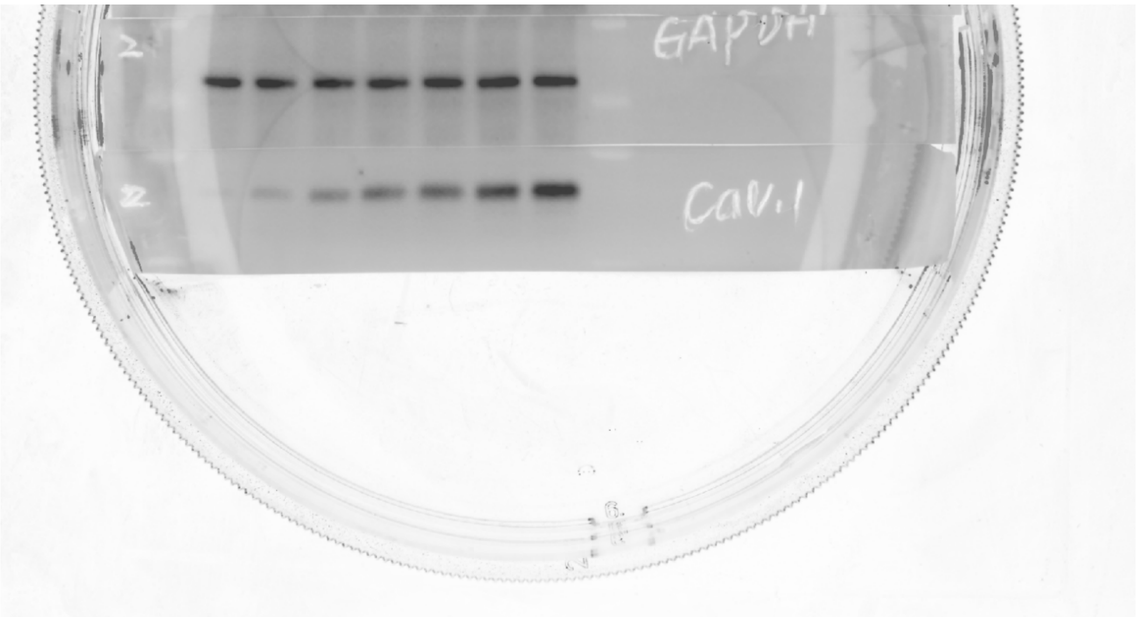

Fig6B-GAPDH&Cav1-4

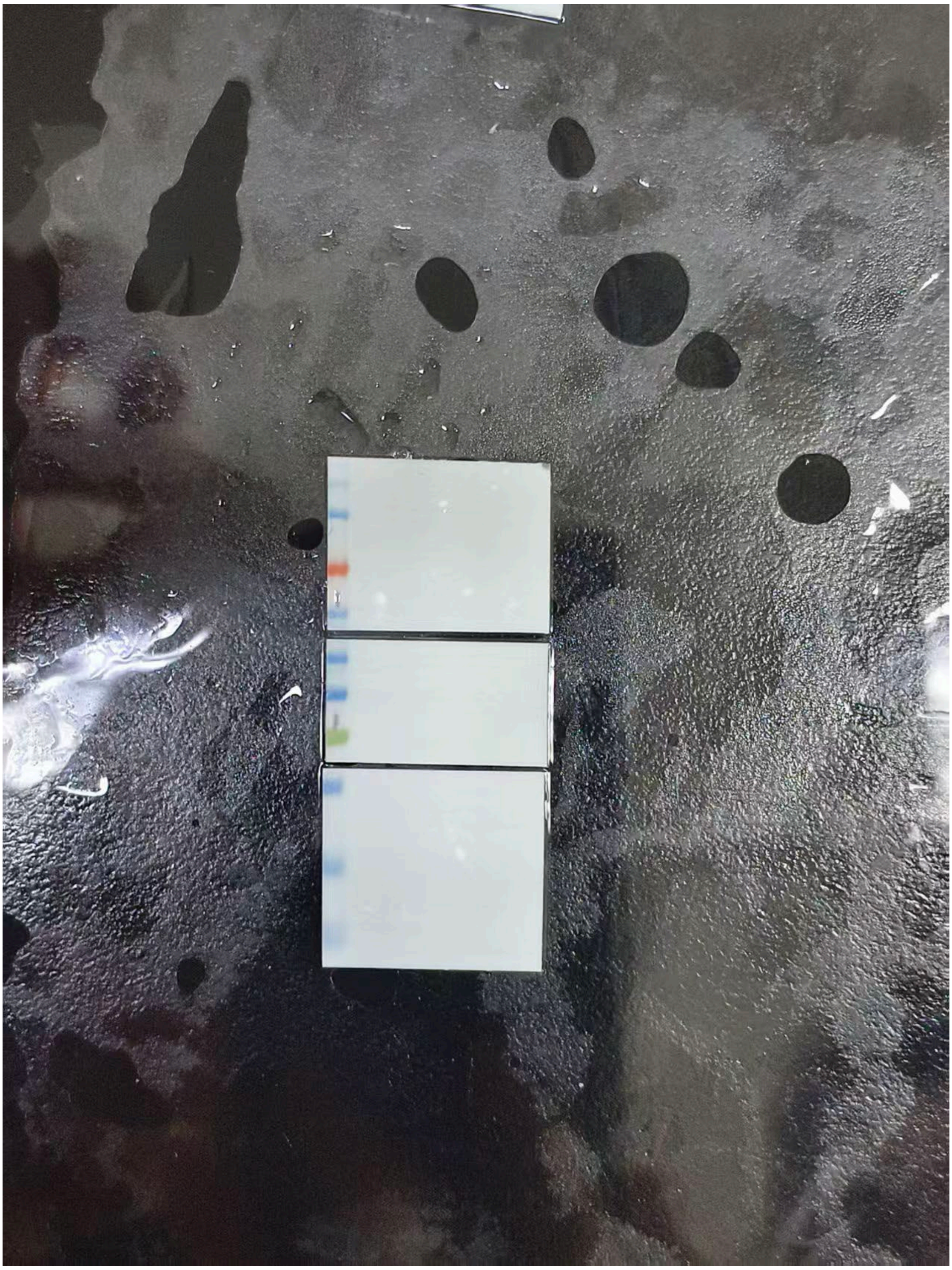

Fig6G-GAP&cGAS-1-全膜

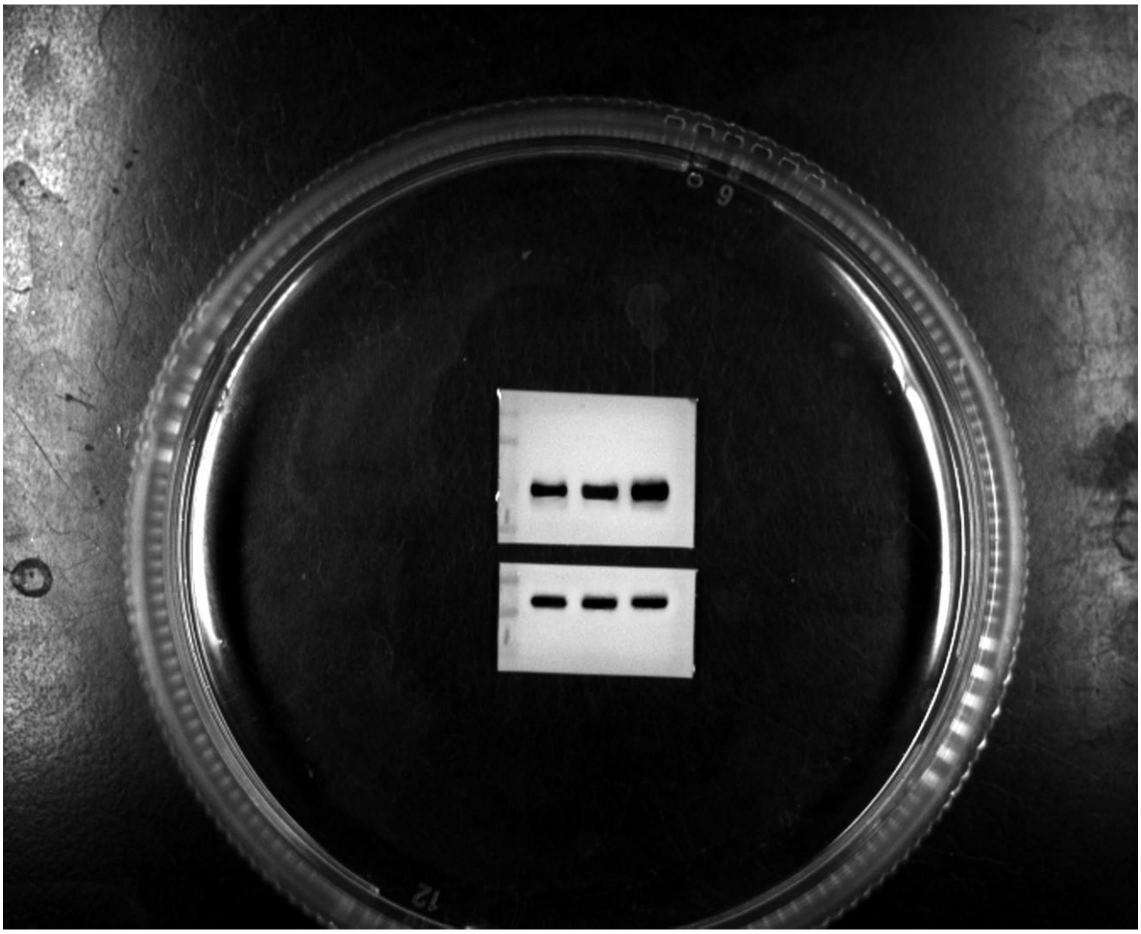

Fig6G-GAP&cGAS-1

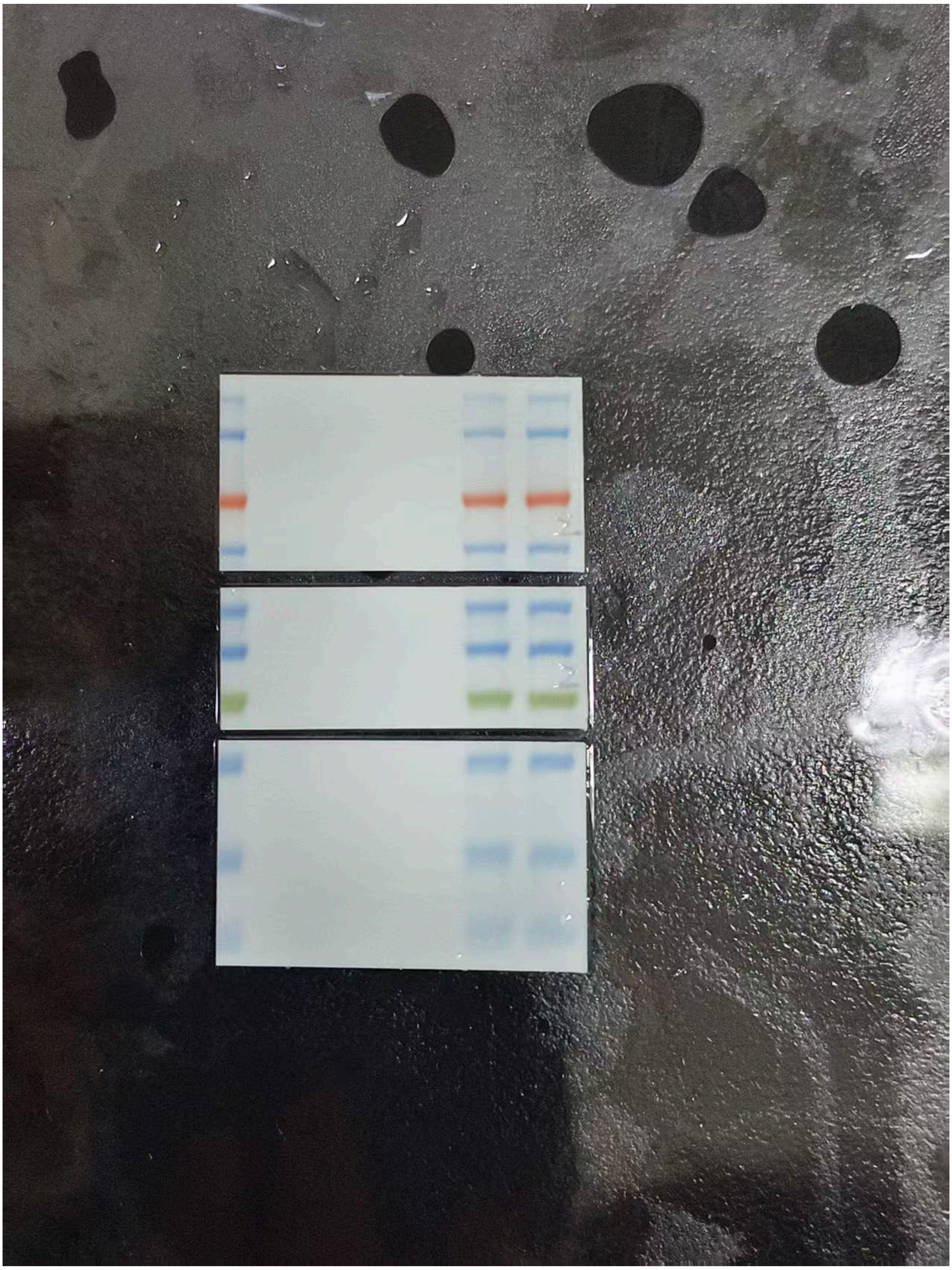

Fig6G-GAP&cGAS-2-全膜

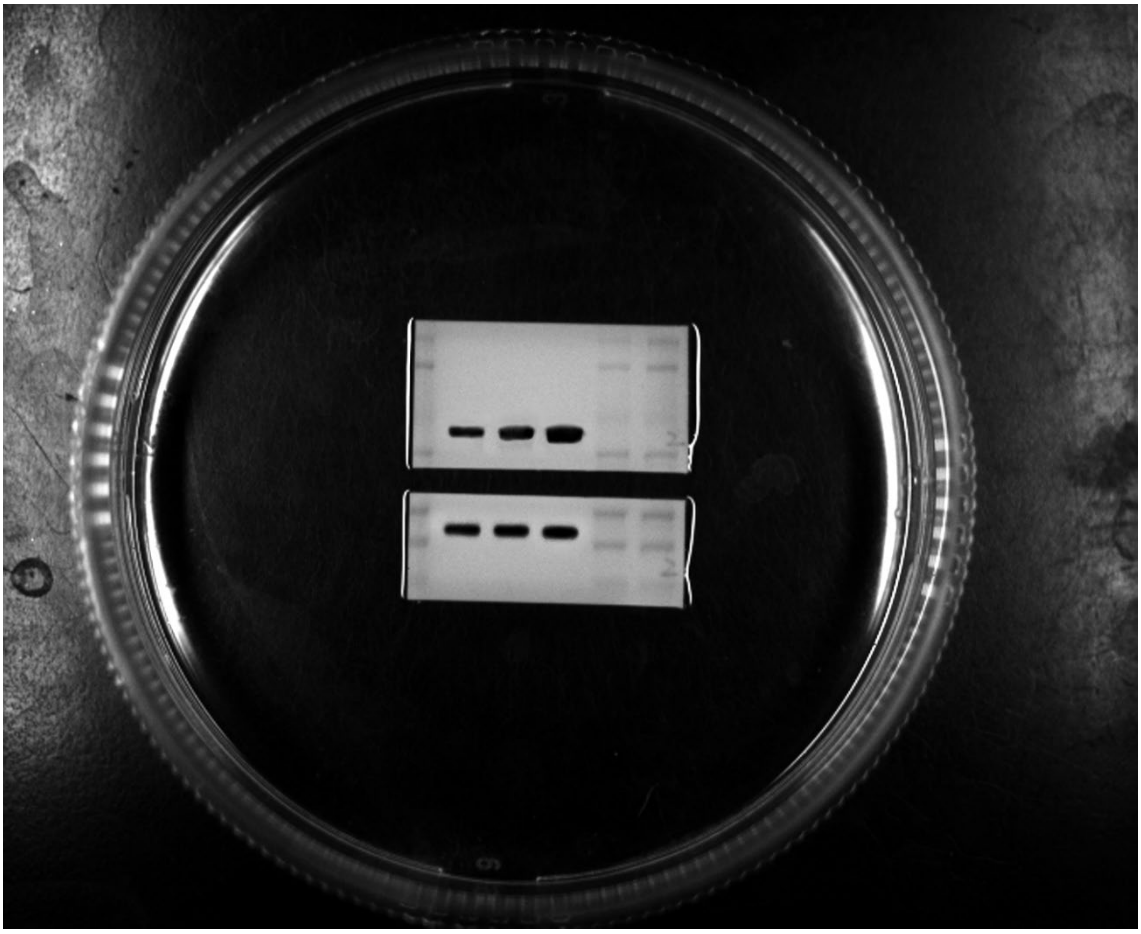

Fig6G-GAP&cGAS-2

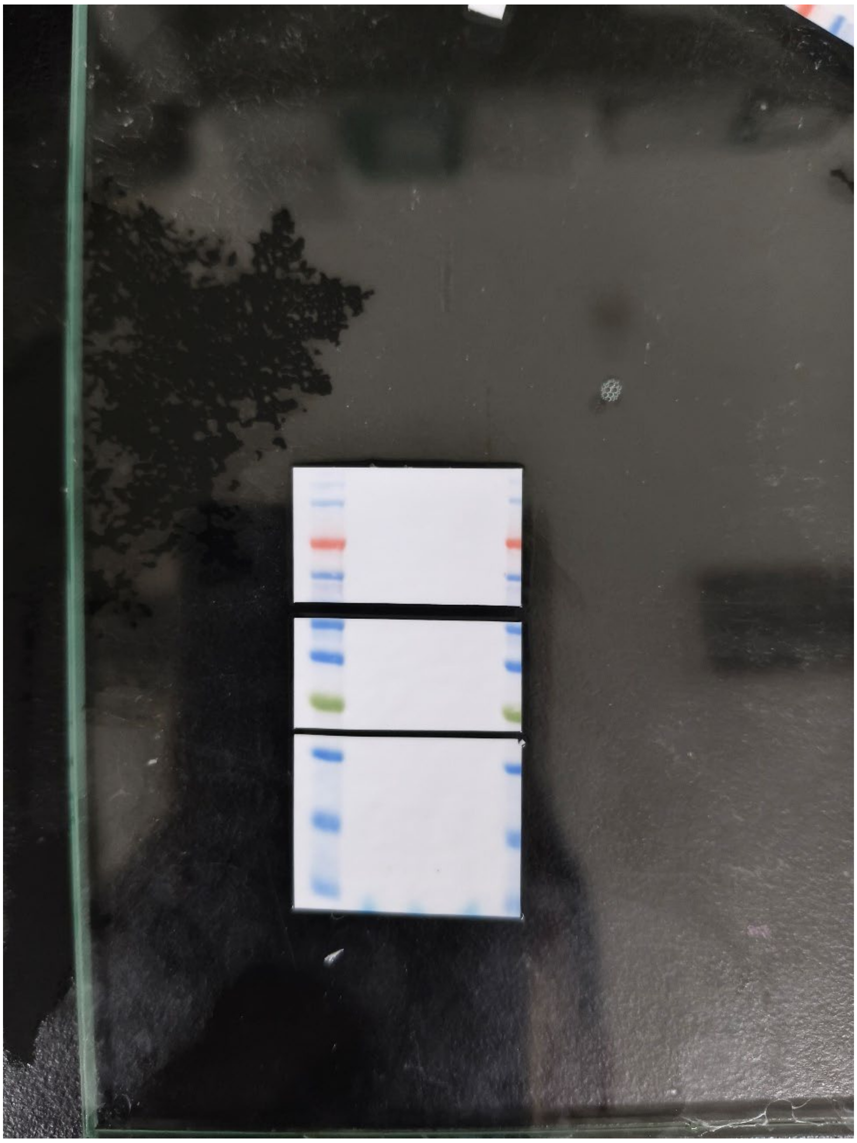

Fig6G-GAP&cGAS-3 全膜

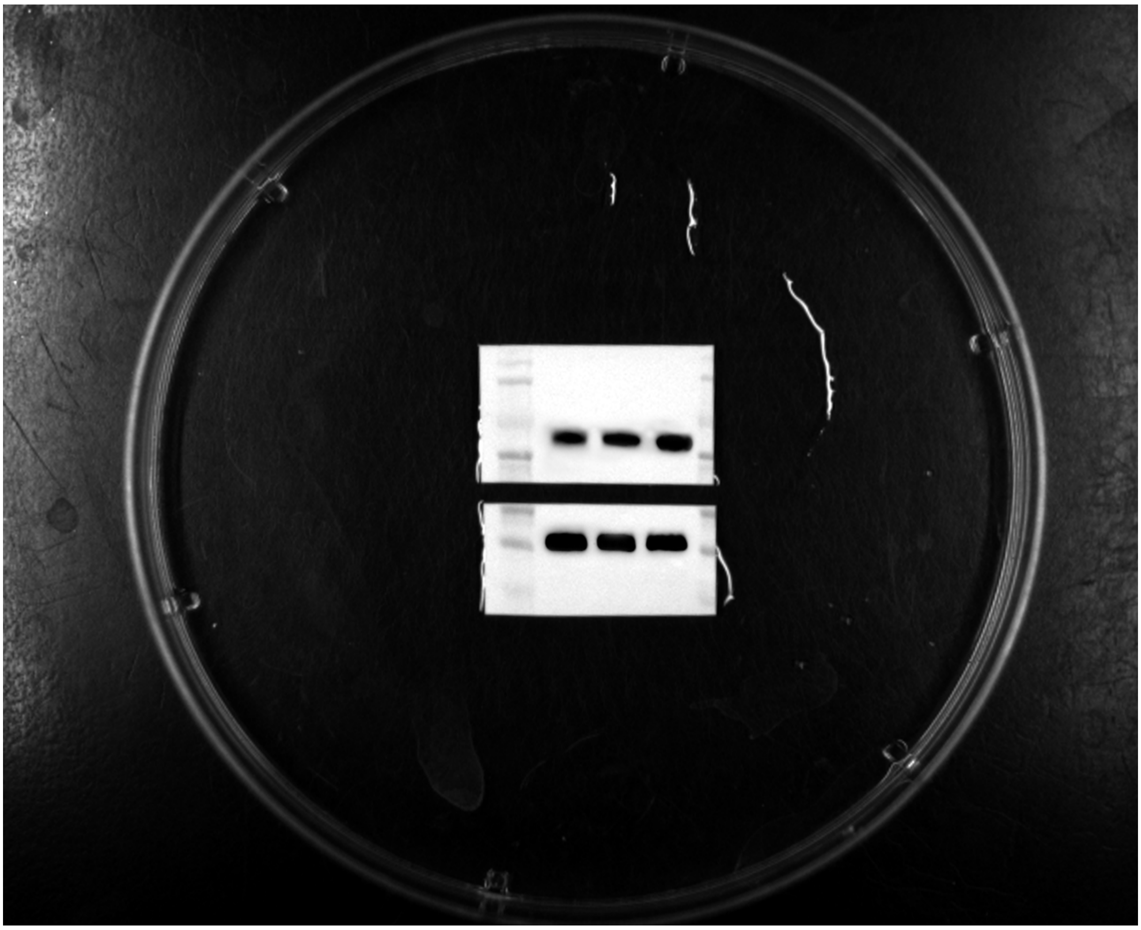

Fig6G-GAP&cGAS-3

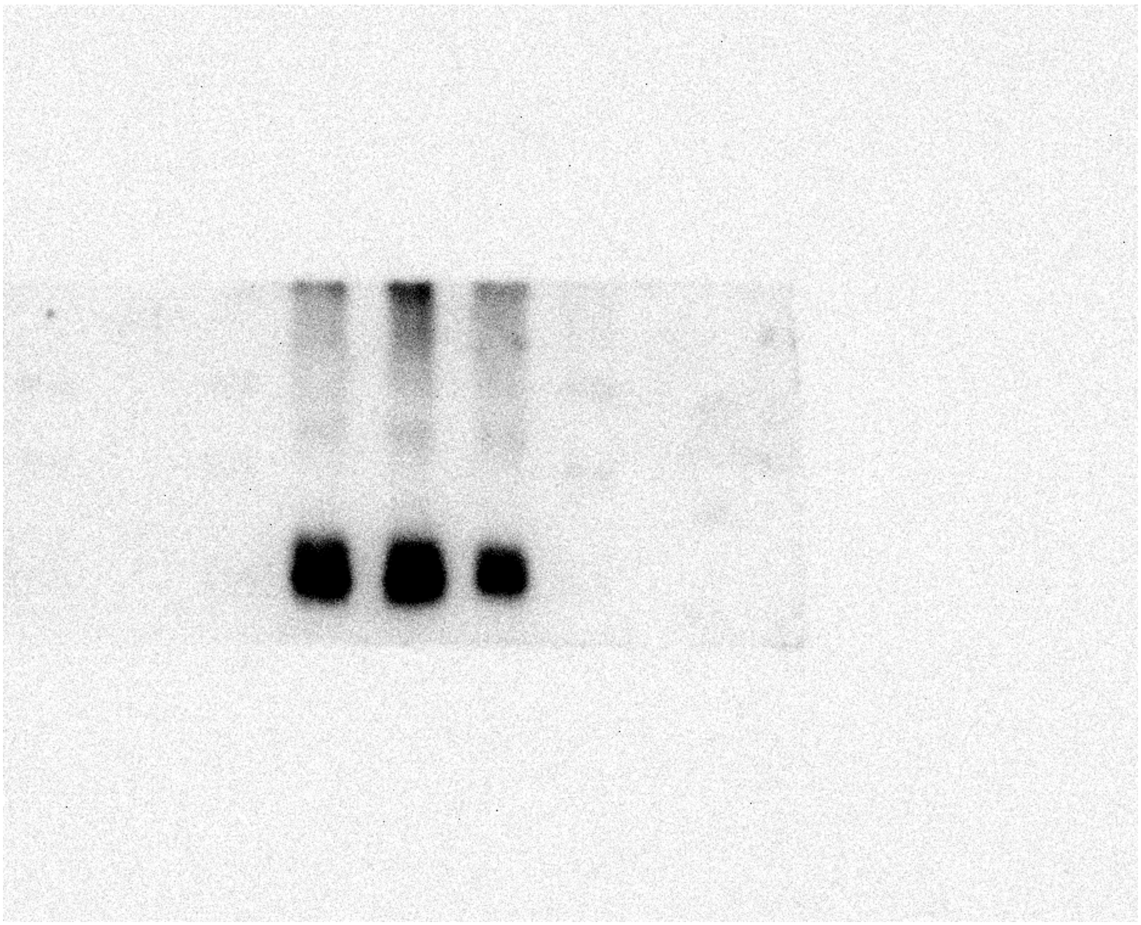

Fig6G-GAPDH-4

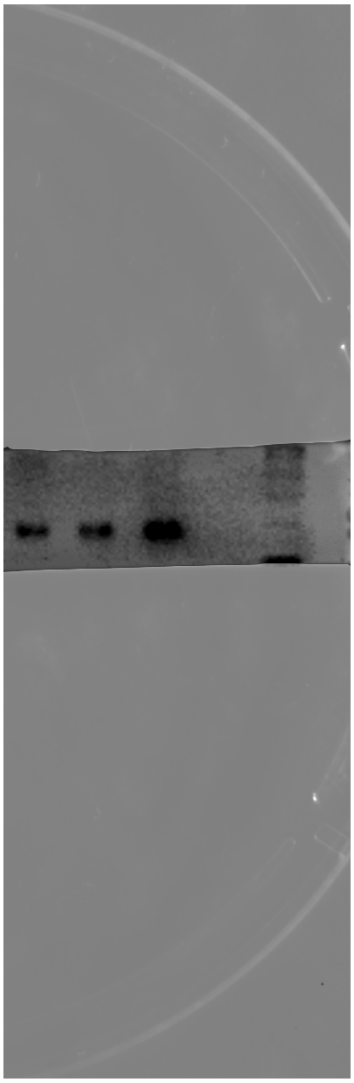

Fig6G-STING-4

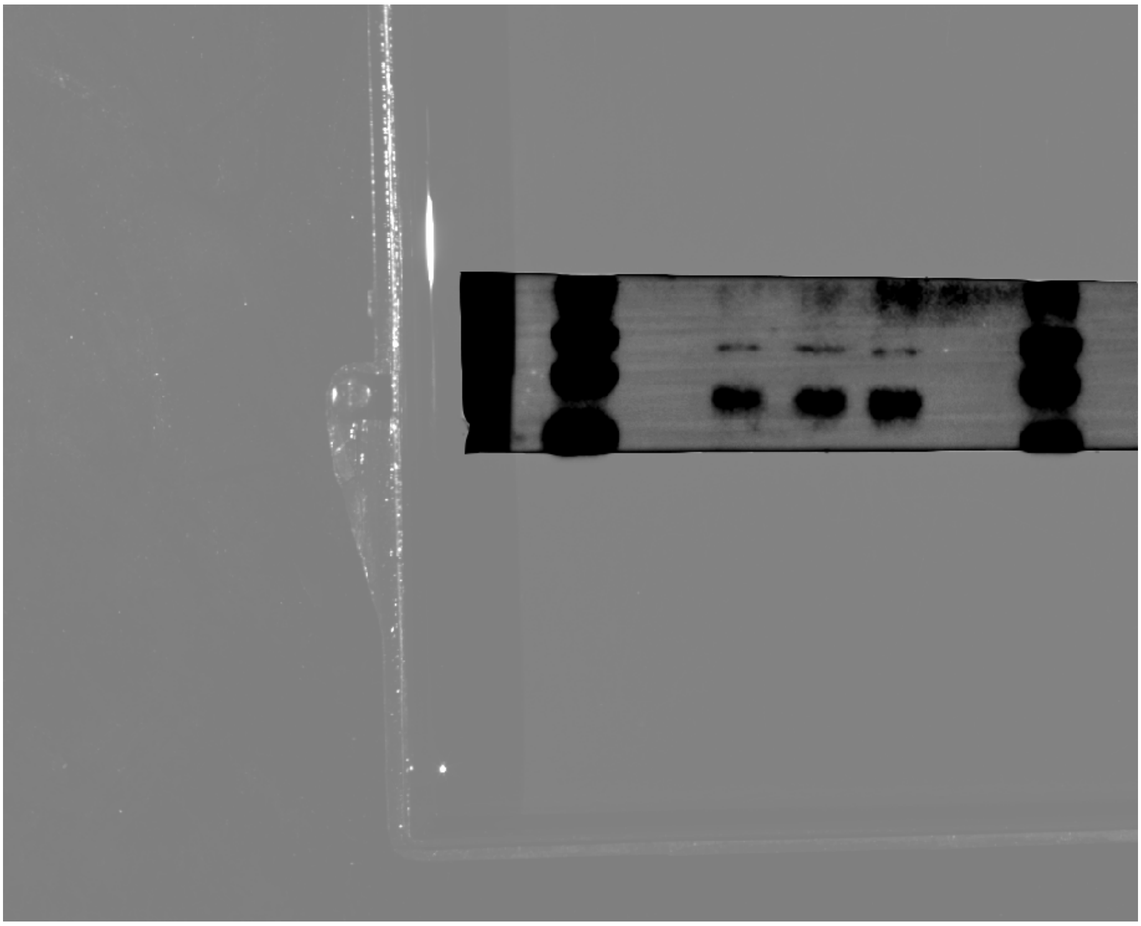

Fig6G-cGAS-4

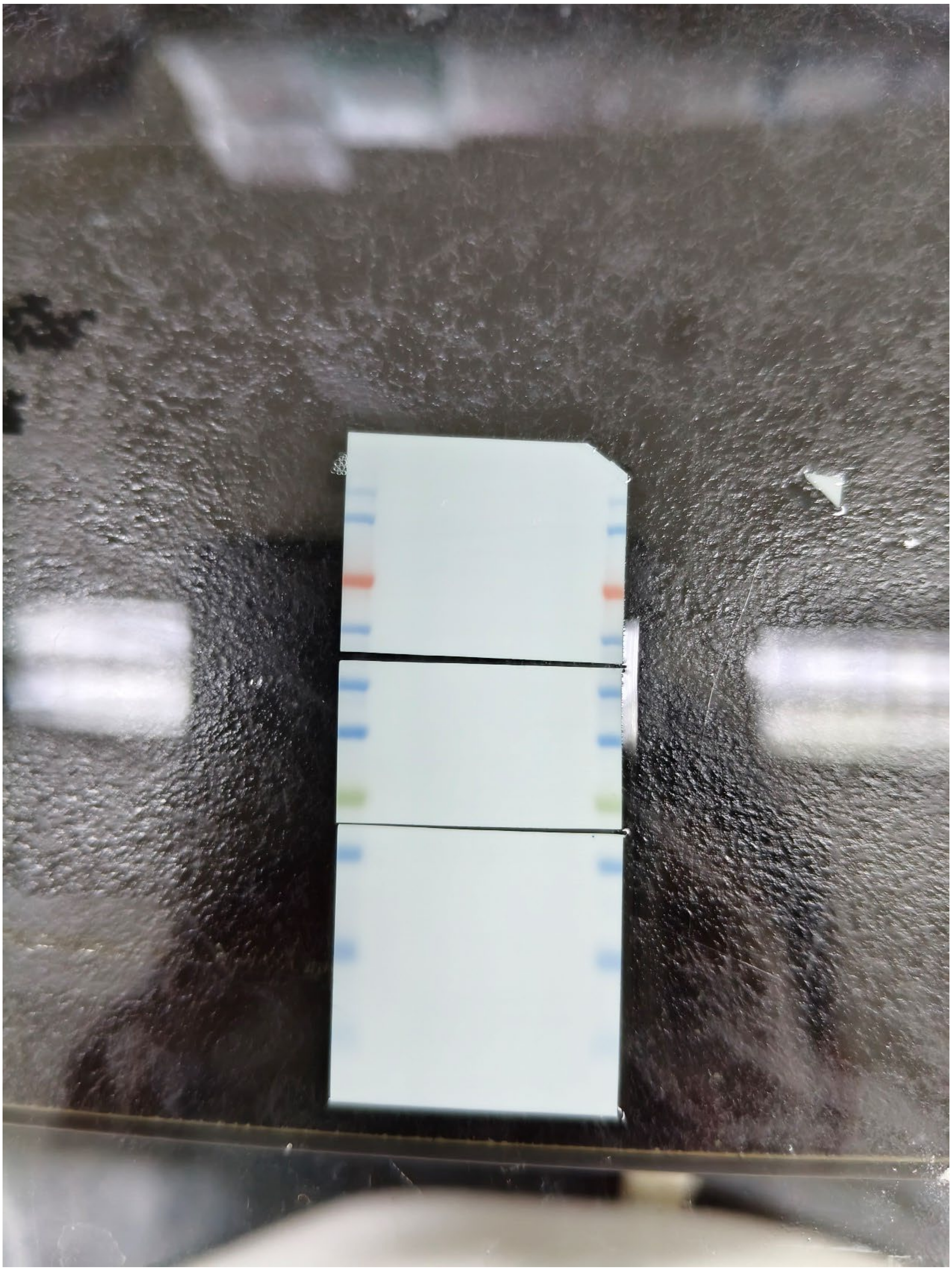

Fig6G-Tublin&STING-1-全膜

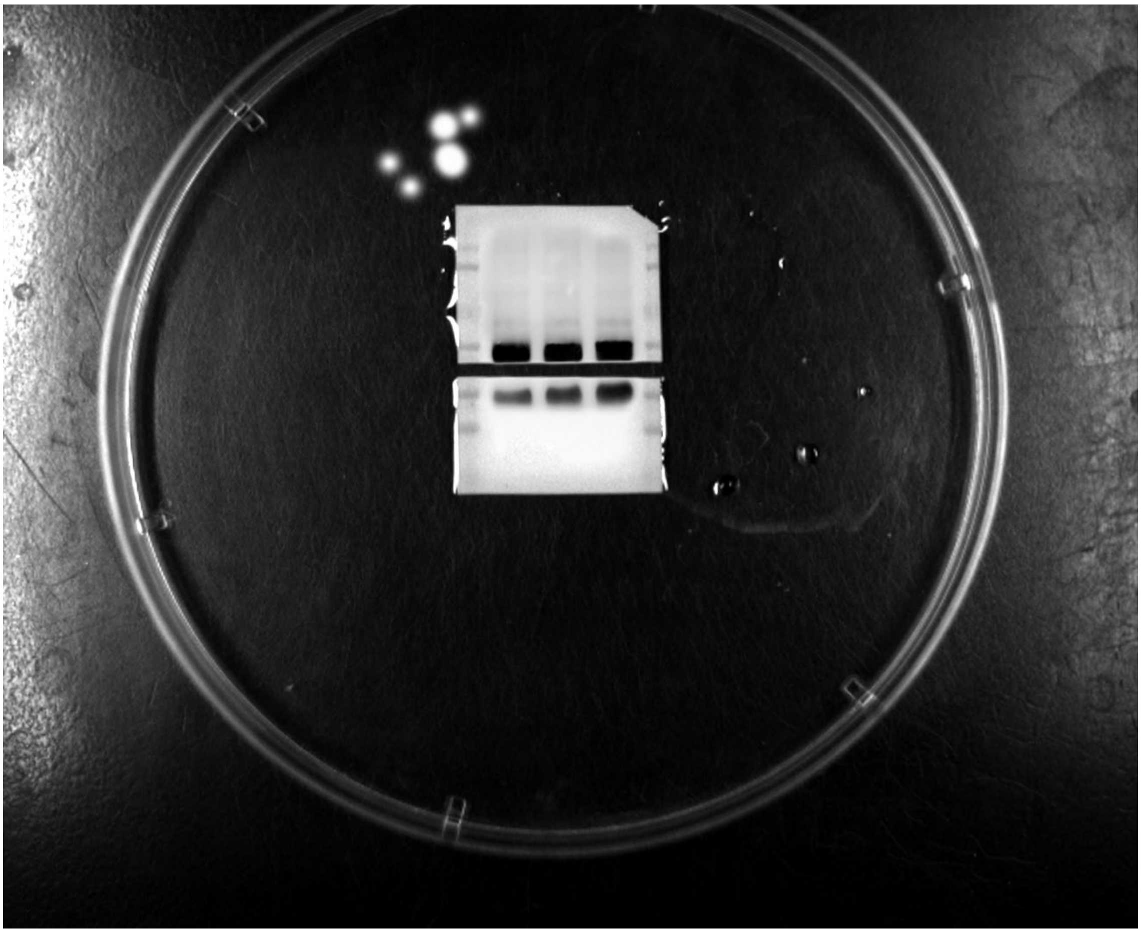

Fig6G-Tublin&STING-1

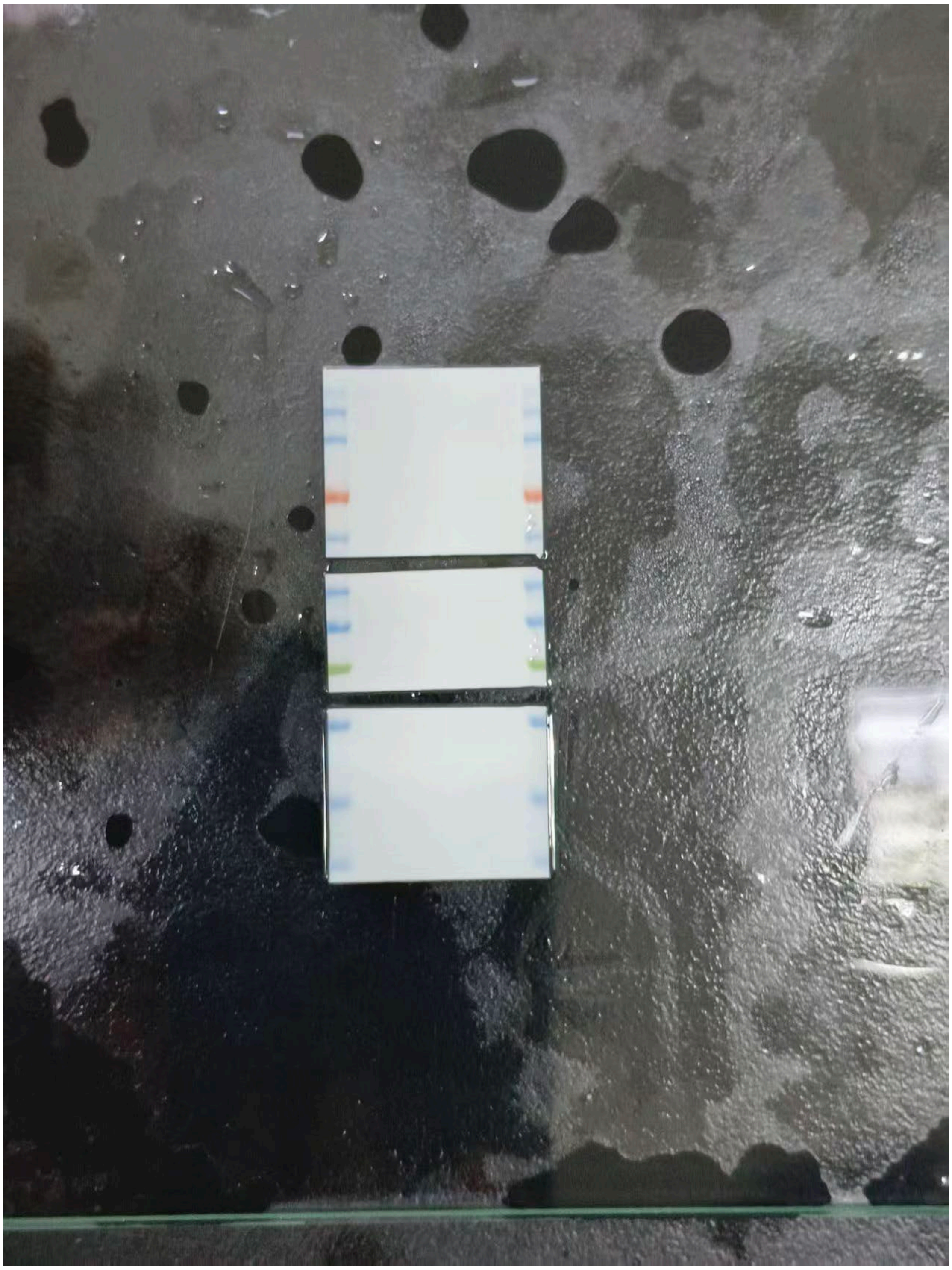

Fig6G-Tublin&STING-2-全膜

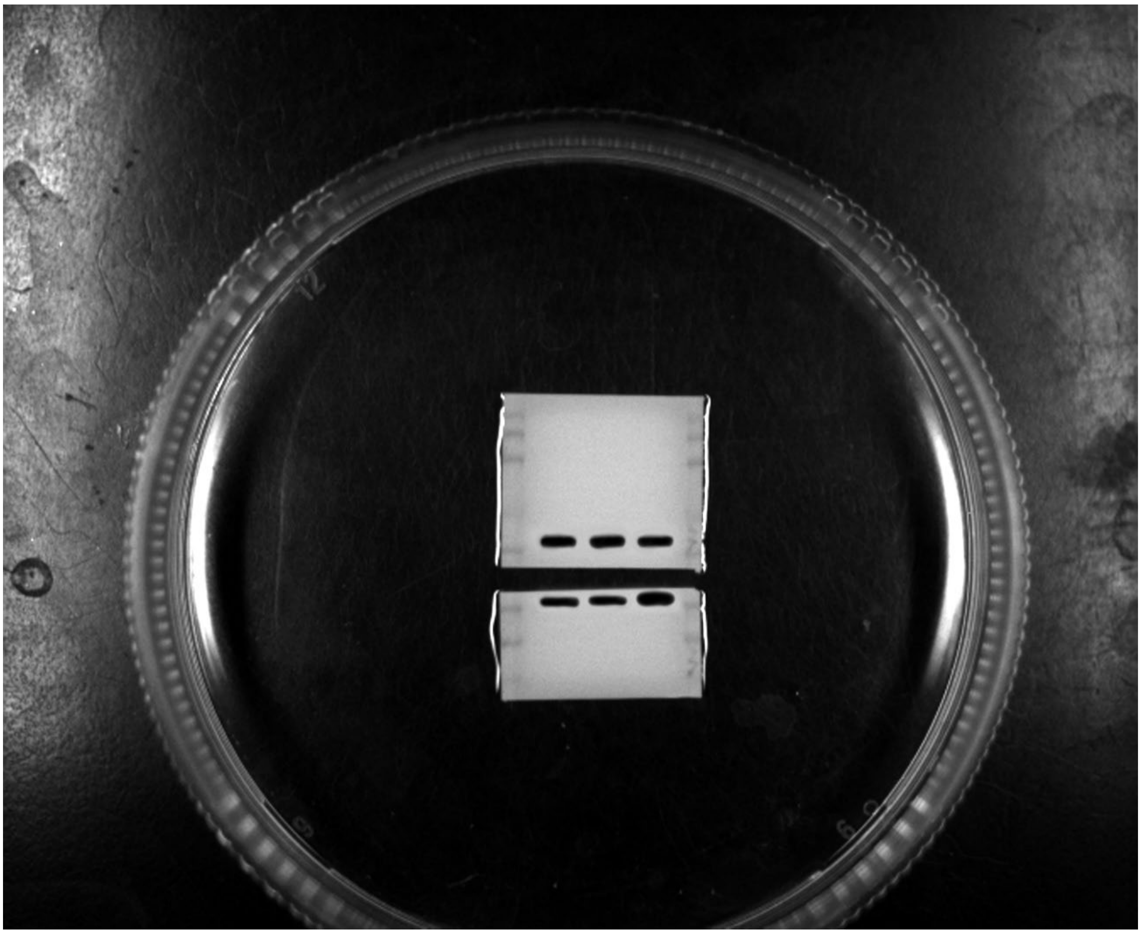

Fig6G-Tublin&STING-2

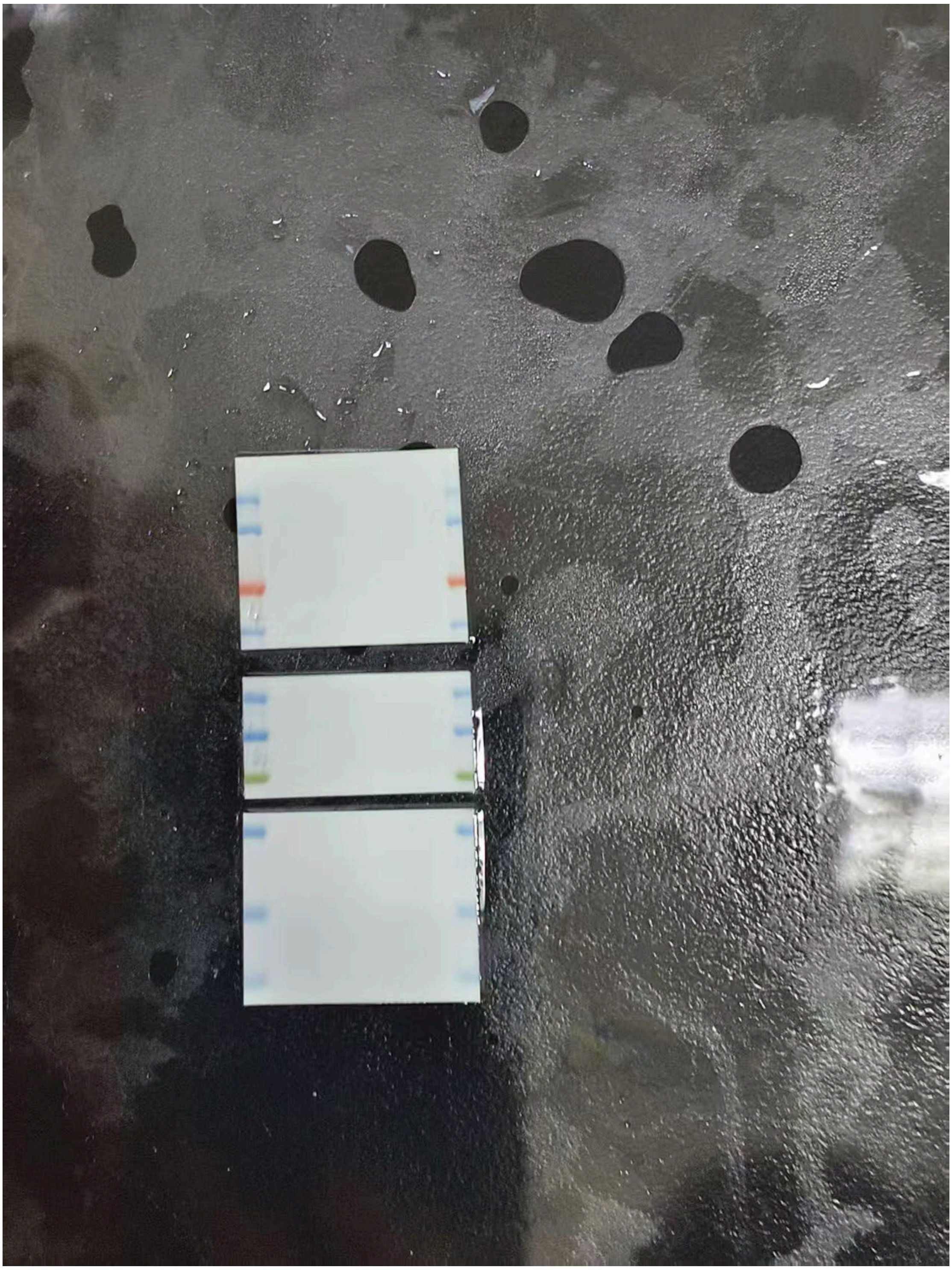

Fig6G-Tublin&STING-3-全膜.tif

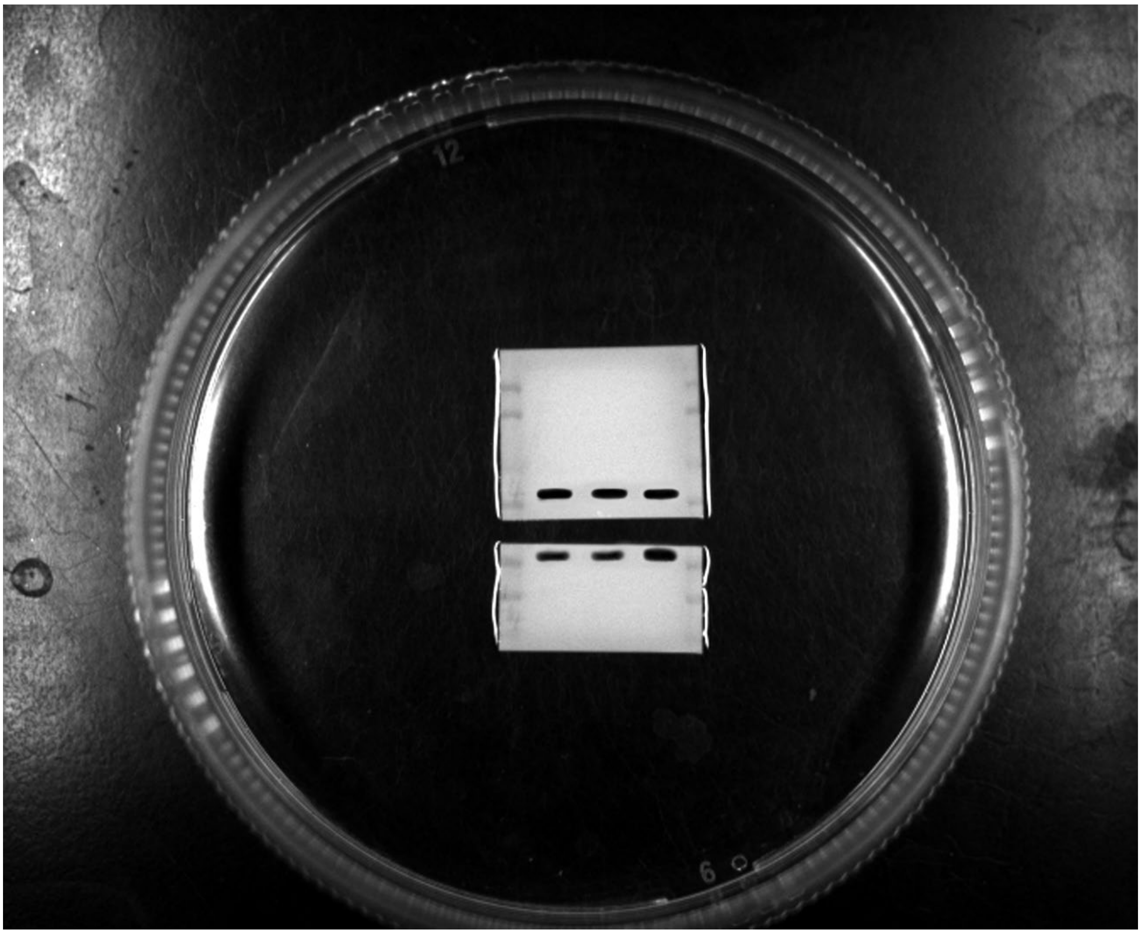

Fig6G-Tublin&STING-3

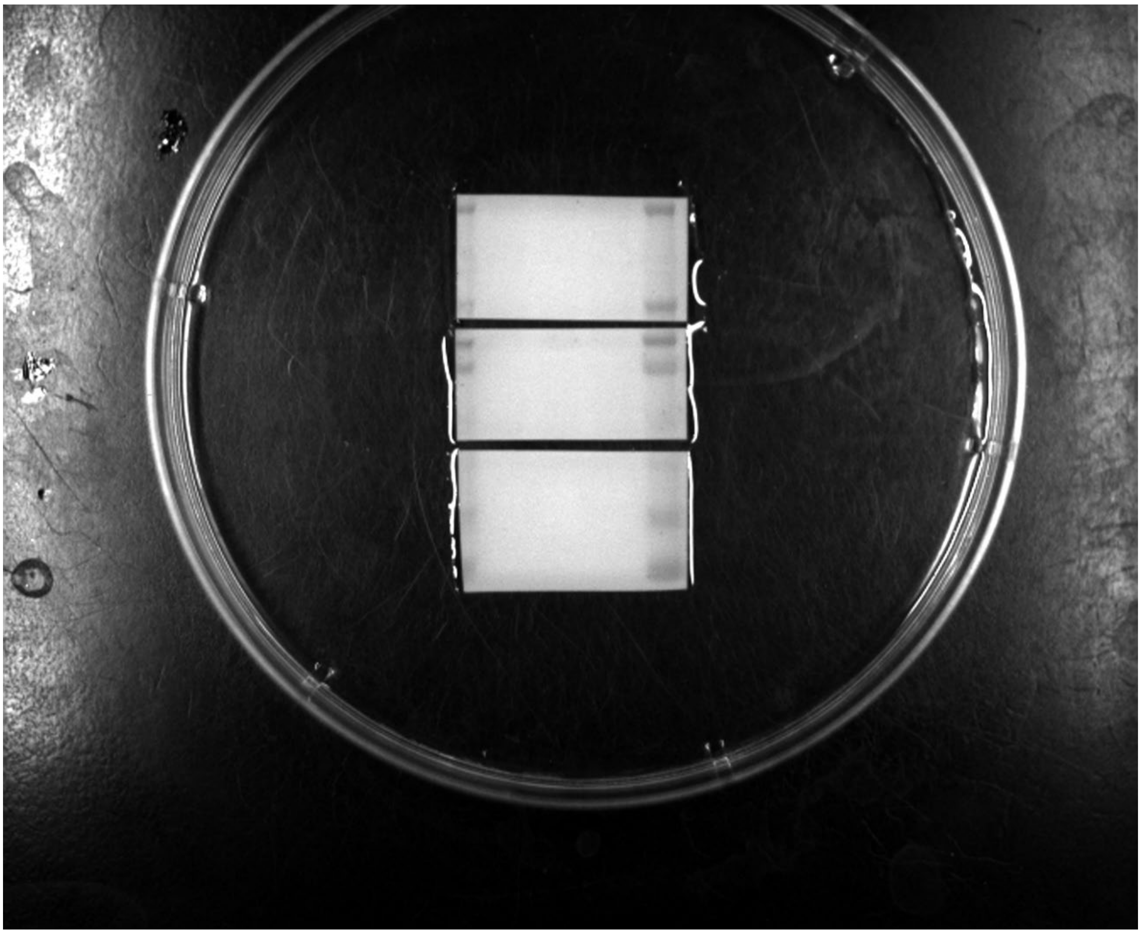

Fig6H-GAPDH&p62&LC3-1-全膜

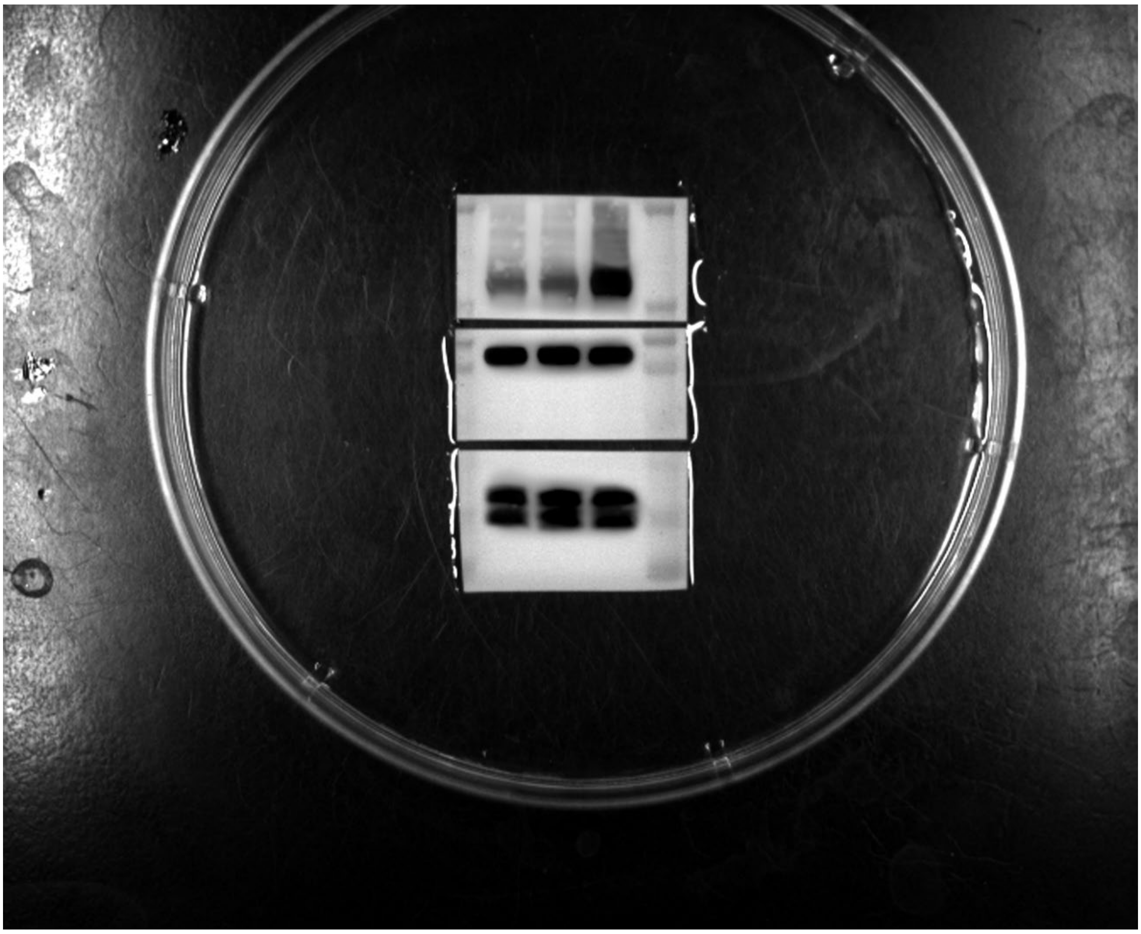

Fig6H-GAPDH&p62&LC3-1

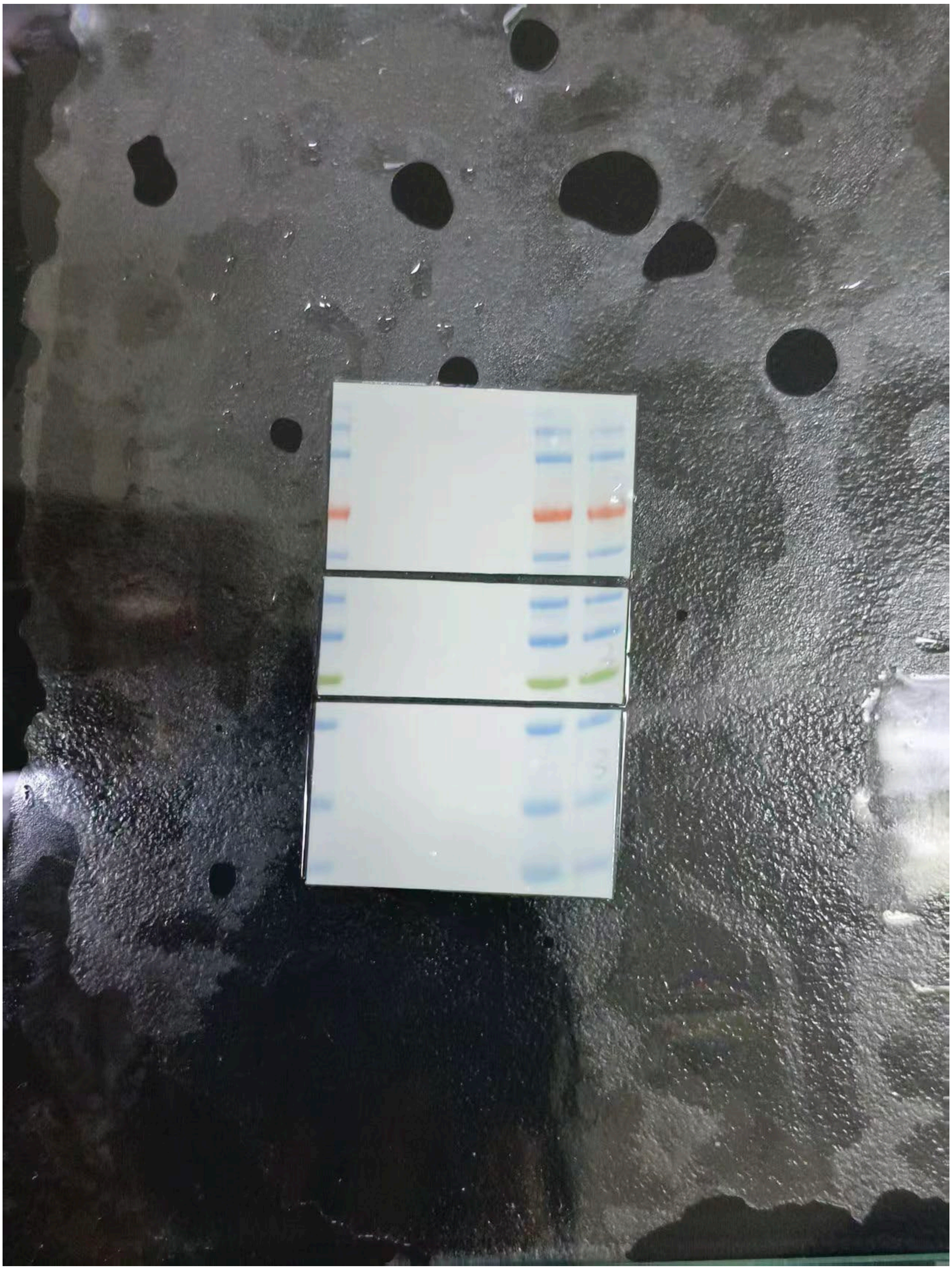

Fig6H-GAPDH&p62&LC3-2-全膜

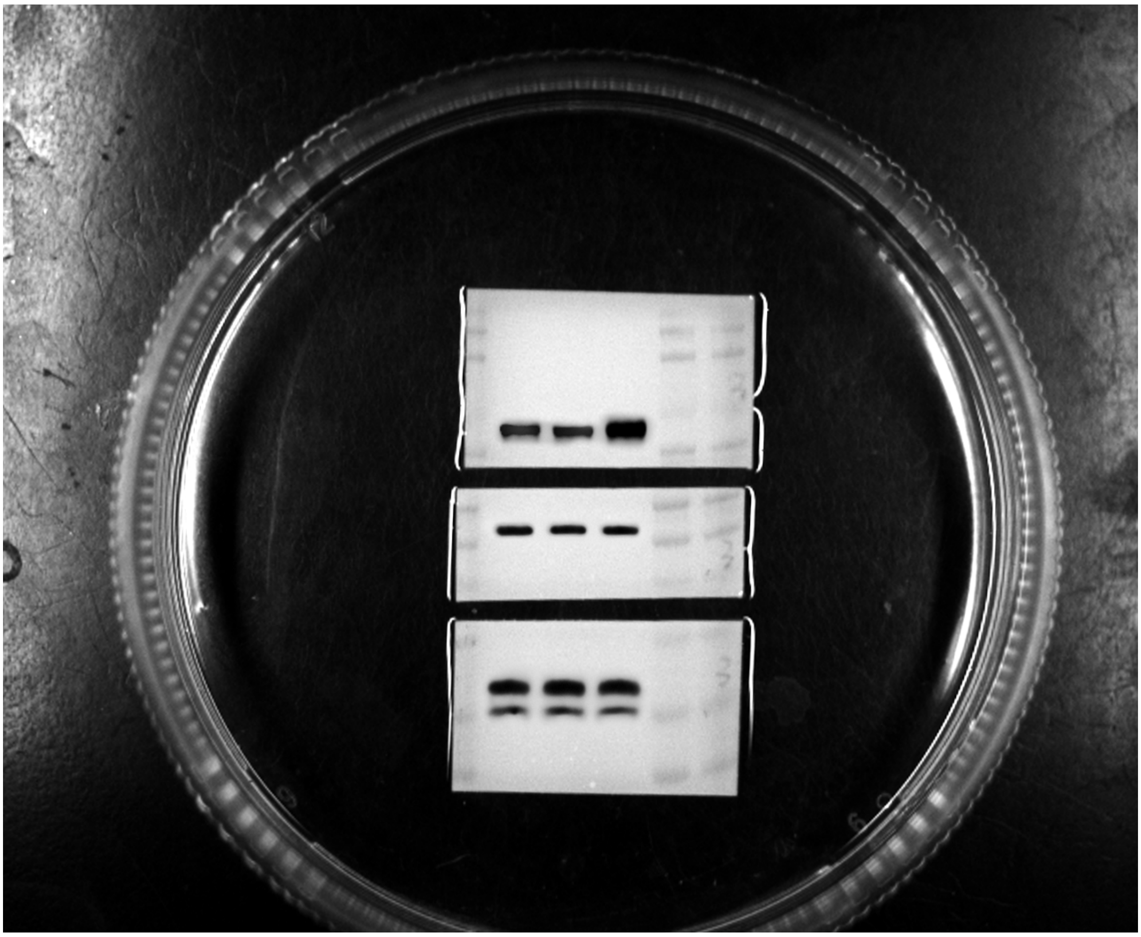

Fig6H-GAPDH&p62&LC3-2

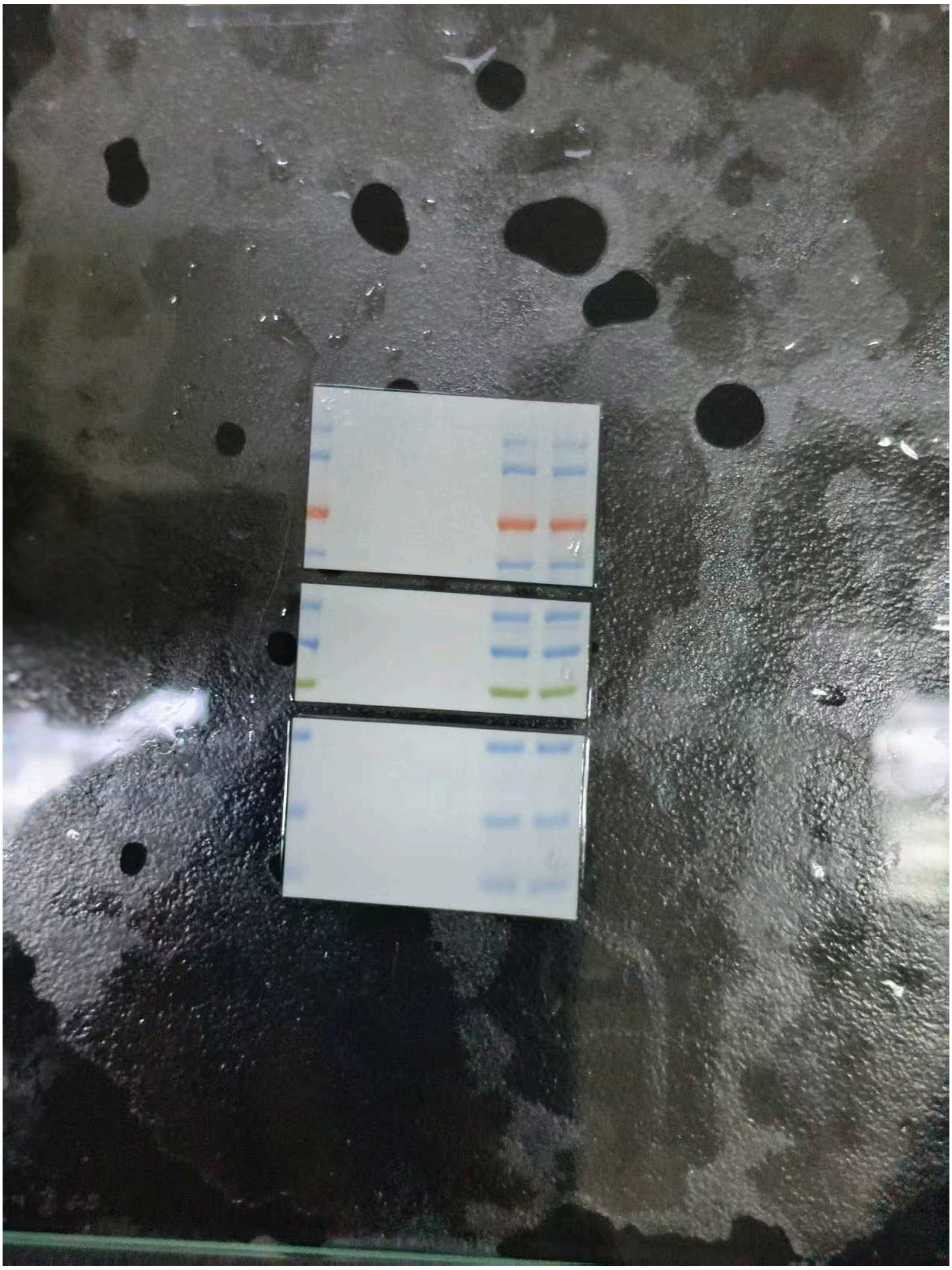

Fig6H-GAPDH&p62&LC3-3-全膜

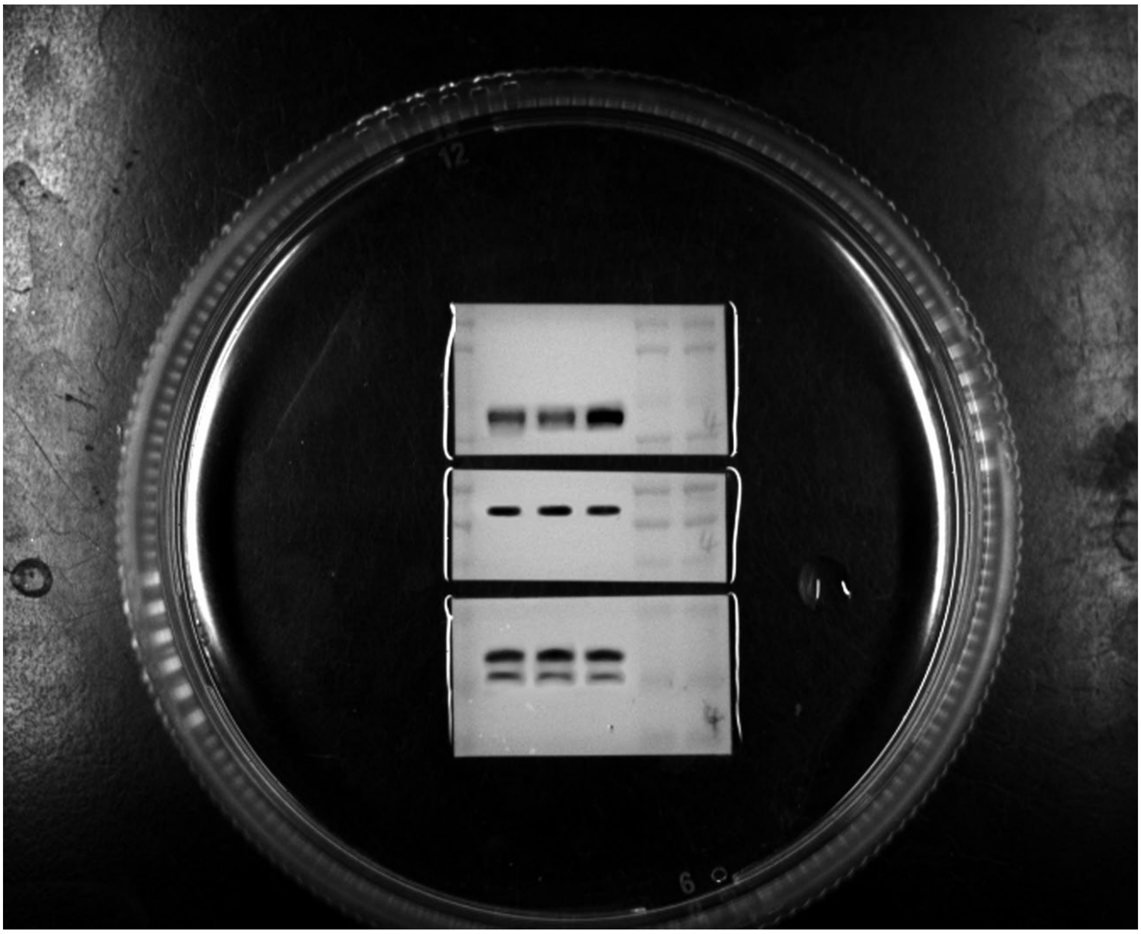

Fig6H-GAPDH&p62&LC3-3

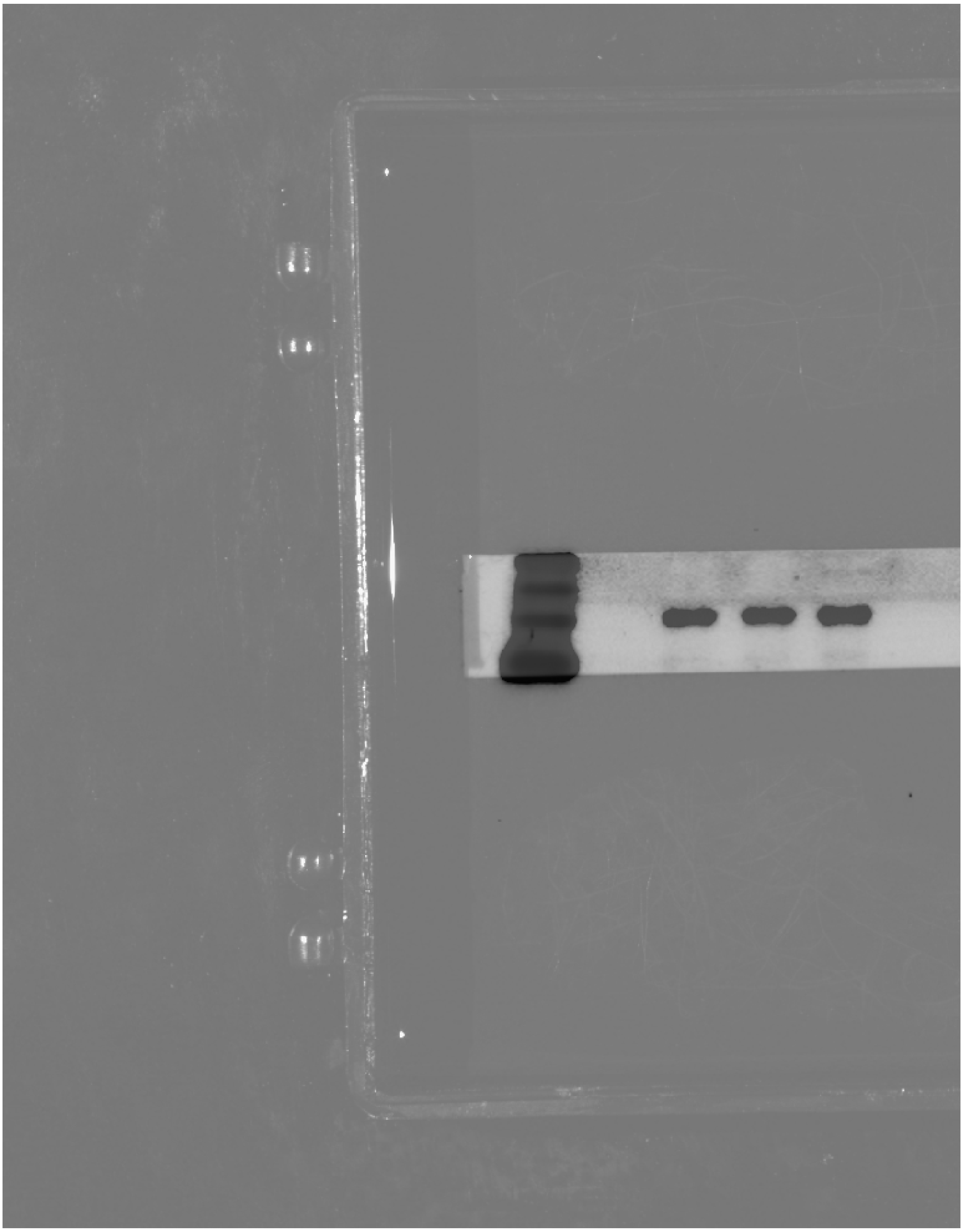

Fig6H-GAPDH-4

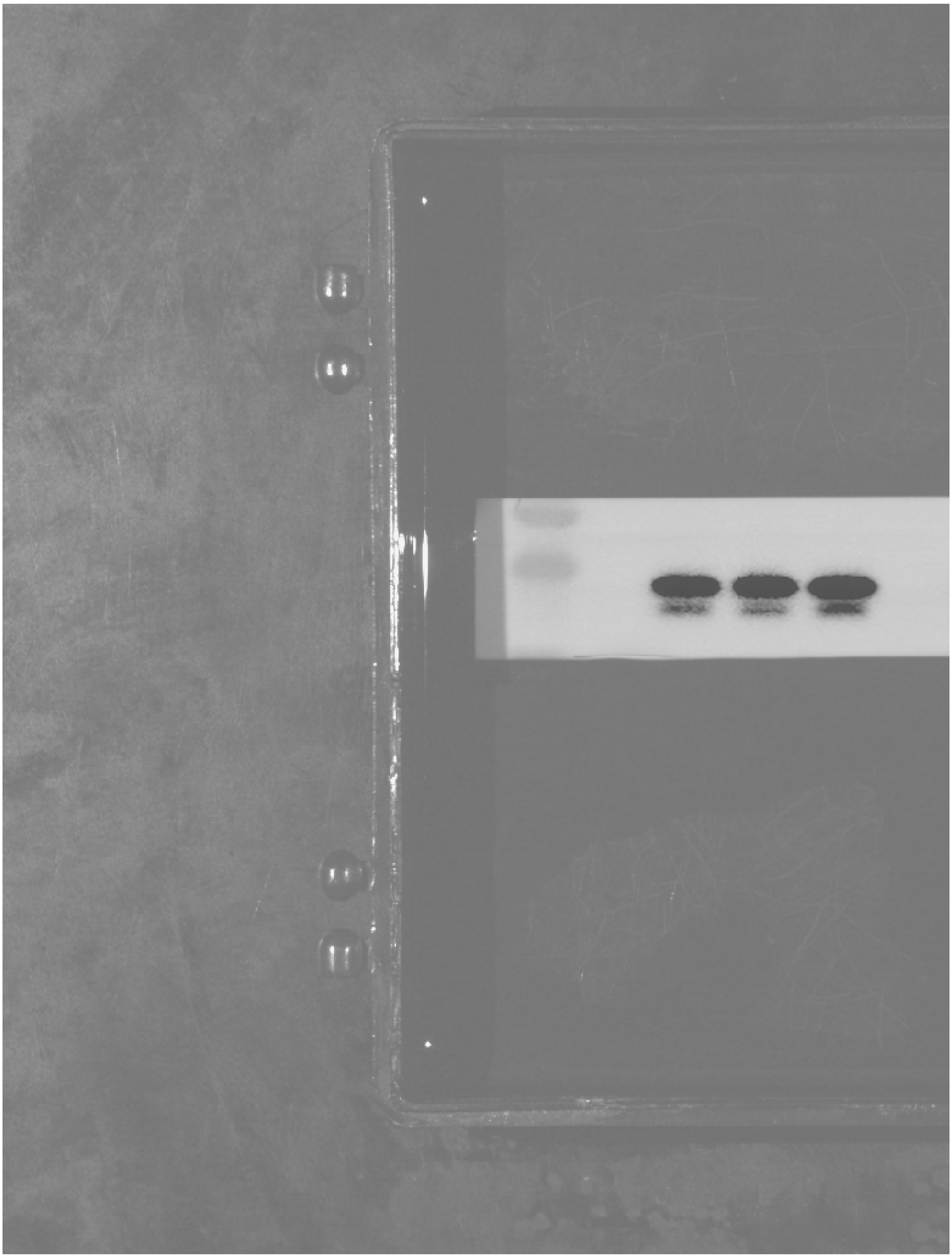

Fig6H-LC3-4

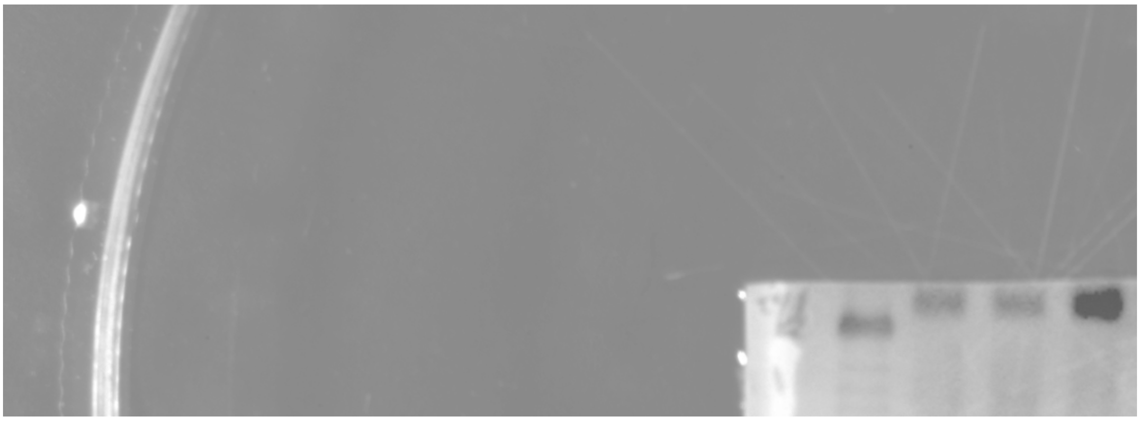

Fig6H-p62-4

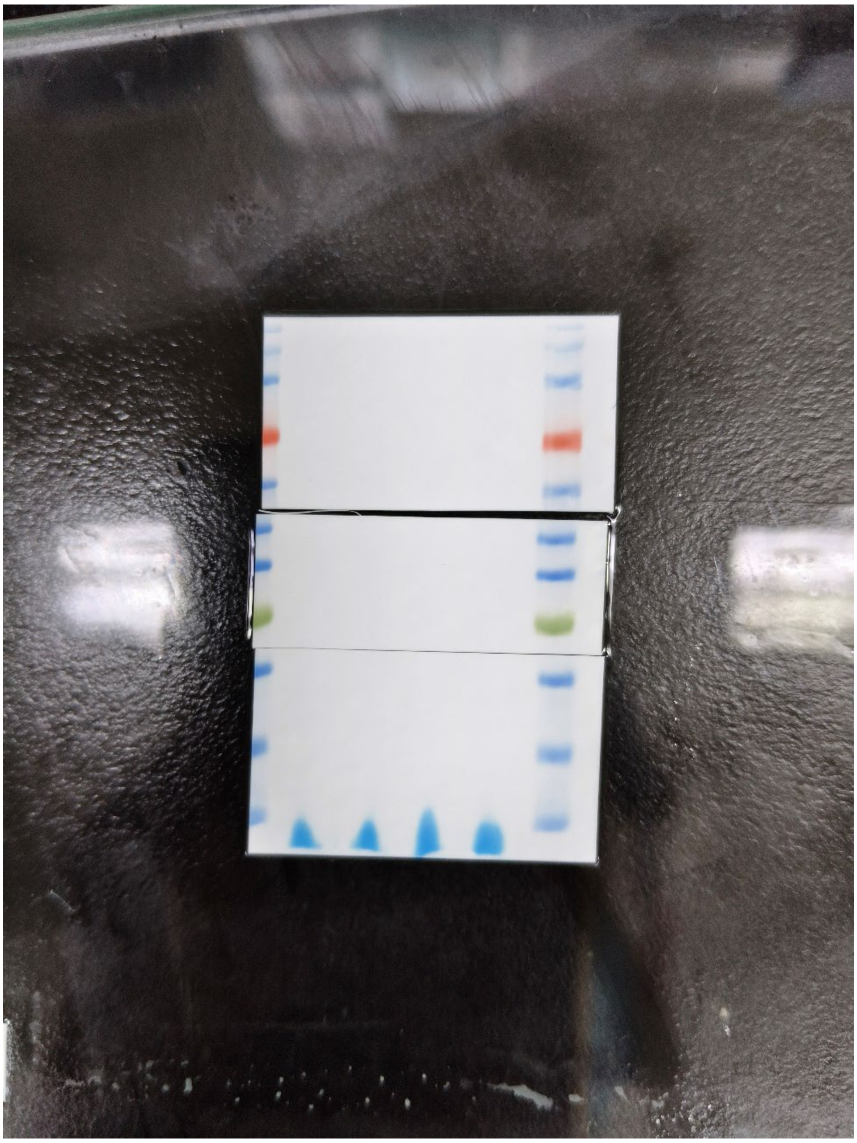

Fig7E-GAPDH&cGAS-1-全膜

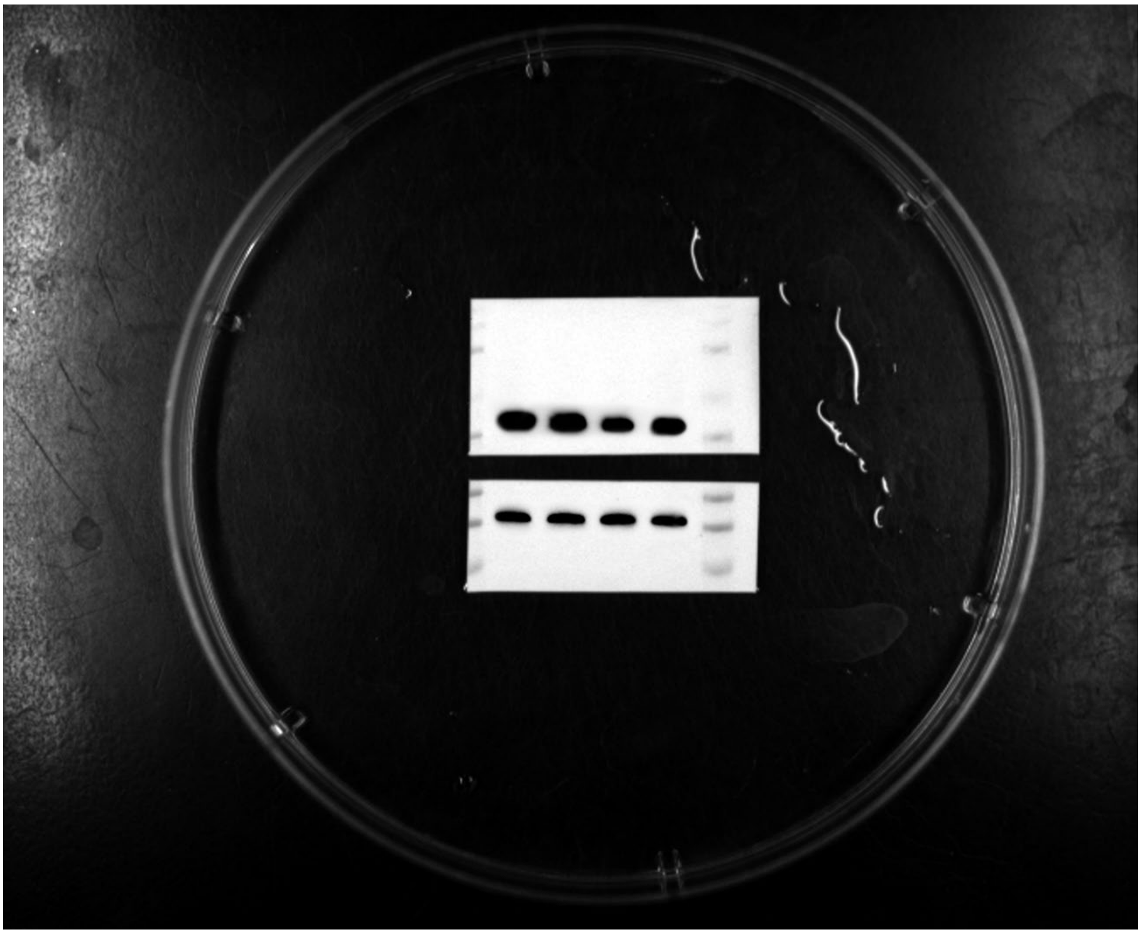

Fig7E-GAPDH&cGAS-1

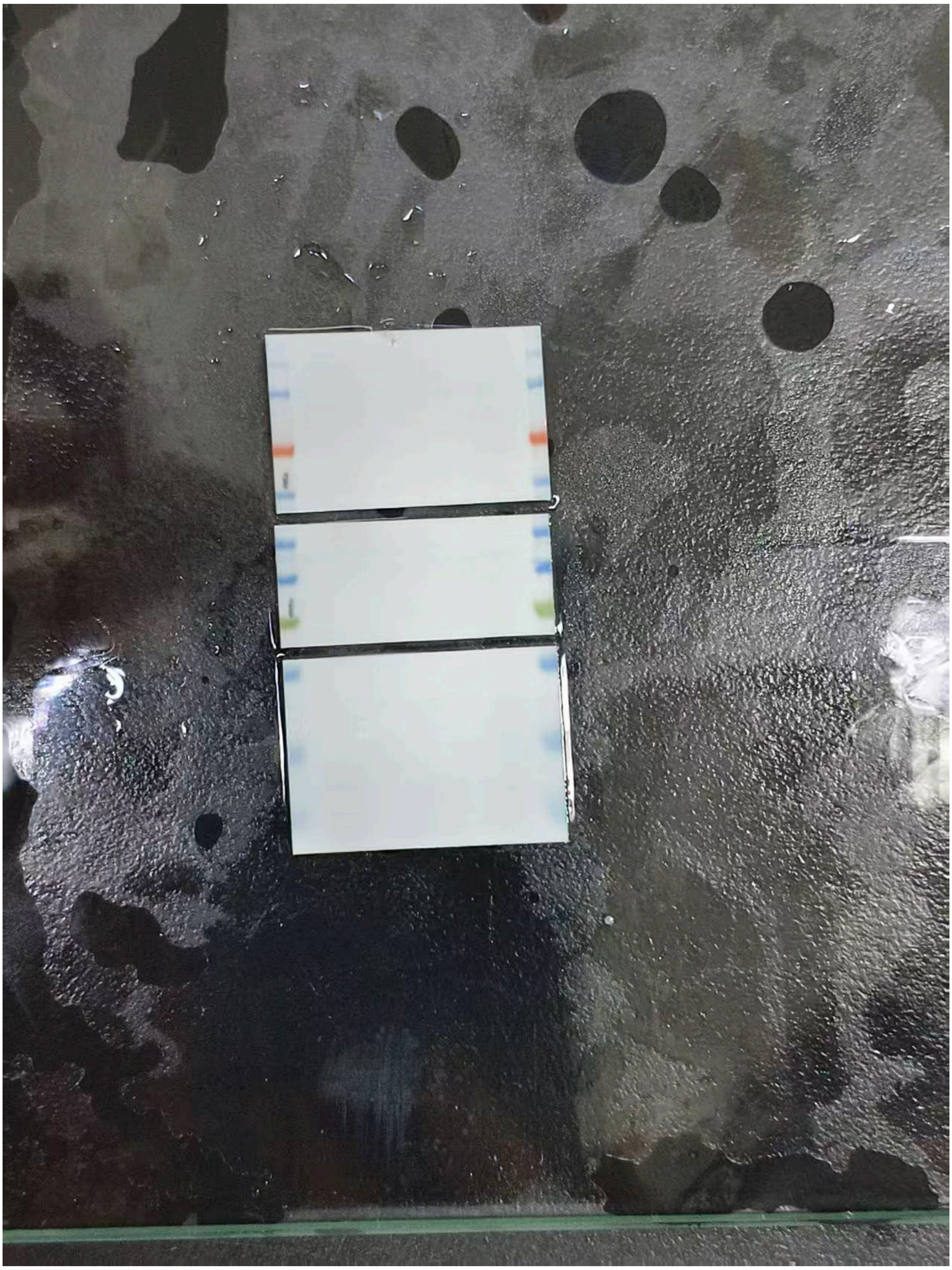

Fig7E-GAPDH&cGAS-2-全膜

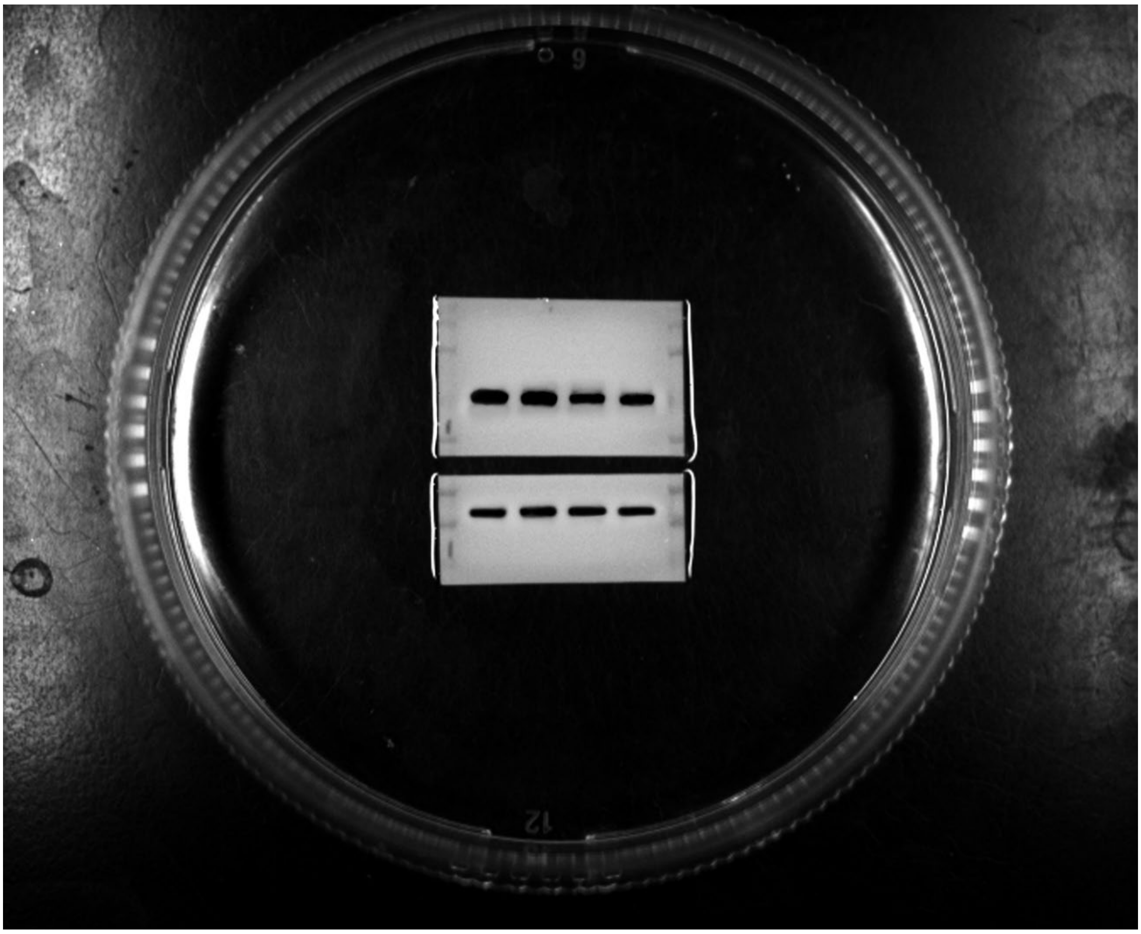

Fig7E-GAPDH&cGAS-2

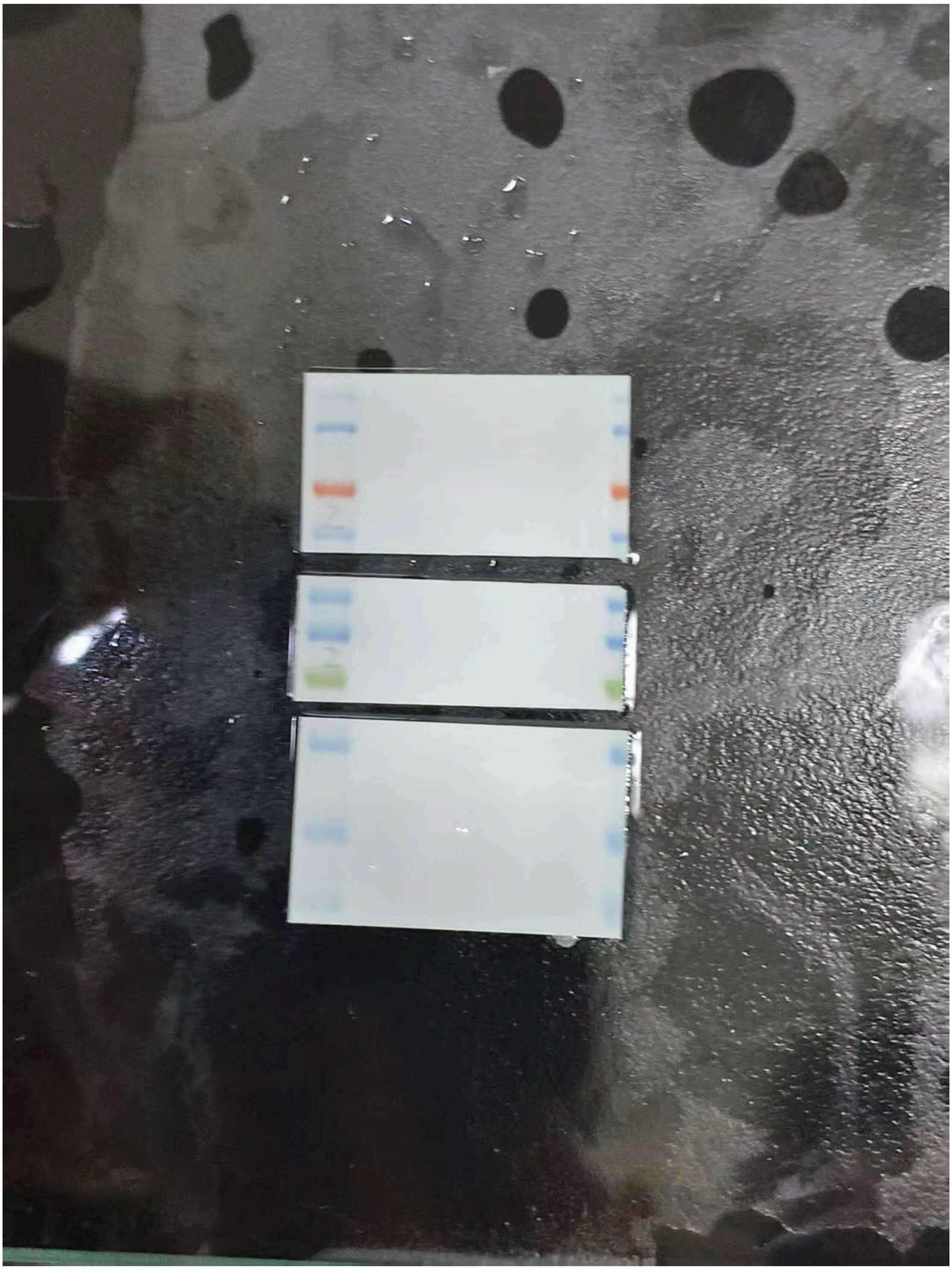

Fig7E-GAPDH&cGAS-3-全膜

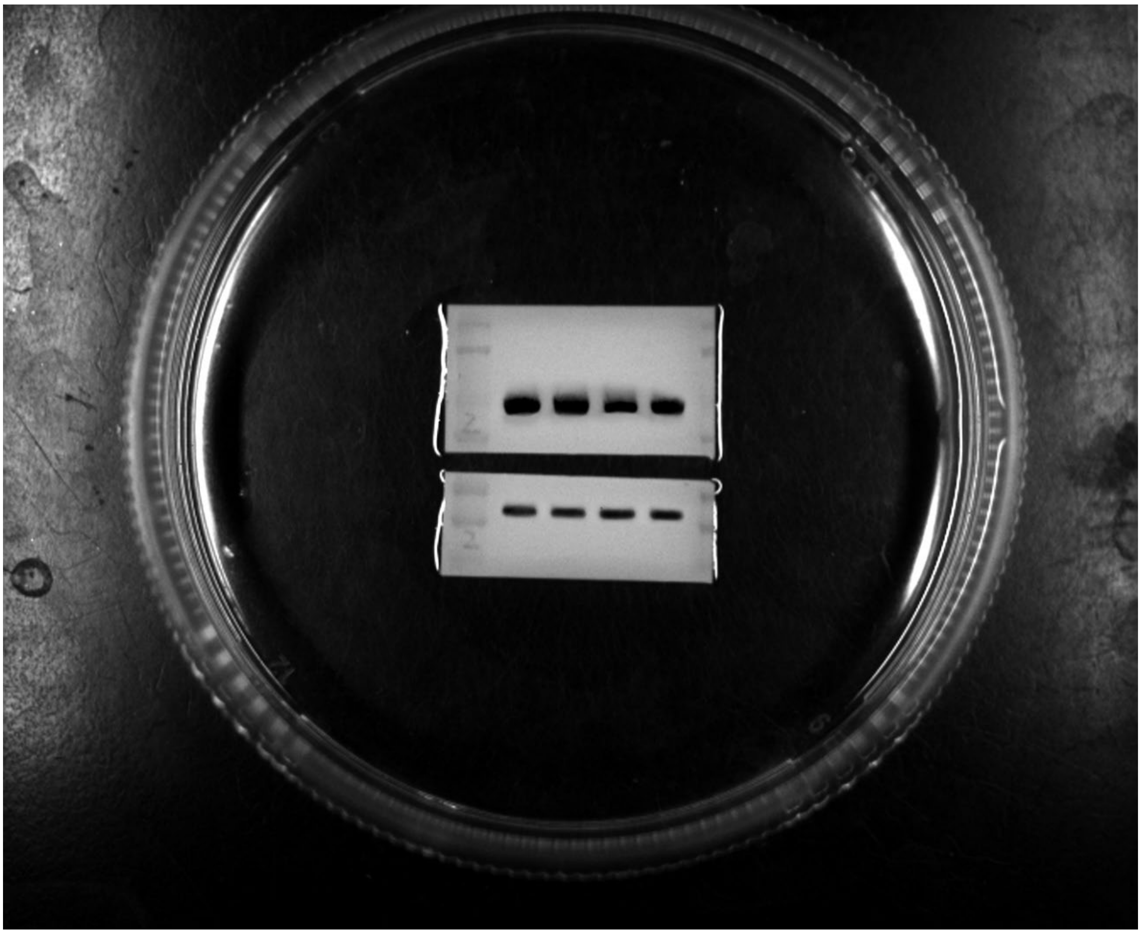

Fig7E-GAPDH&cGAS-3

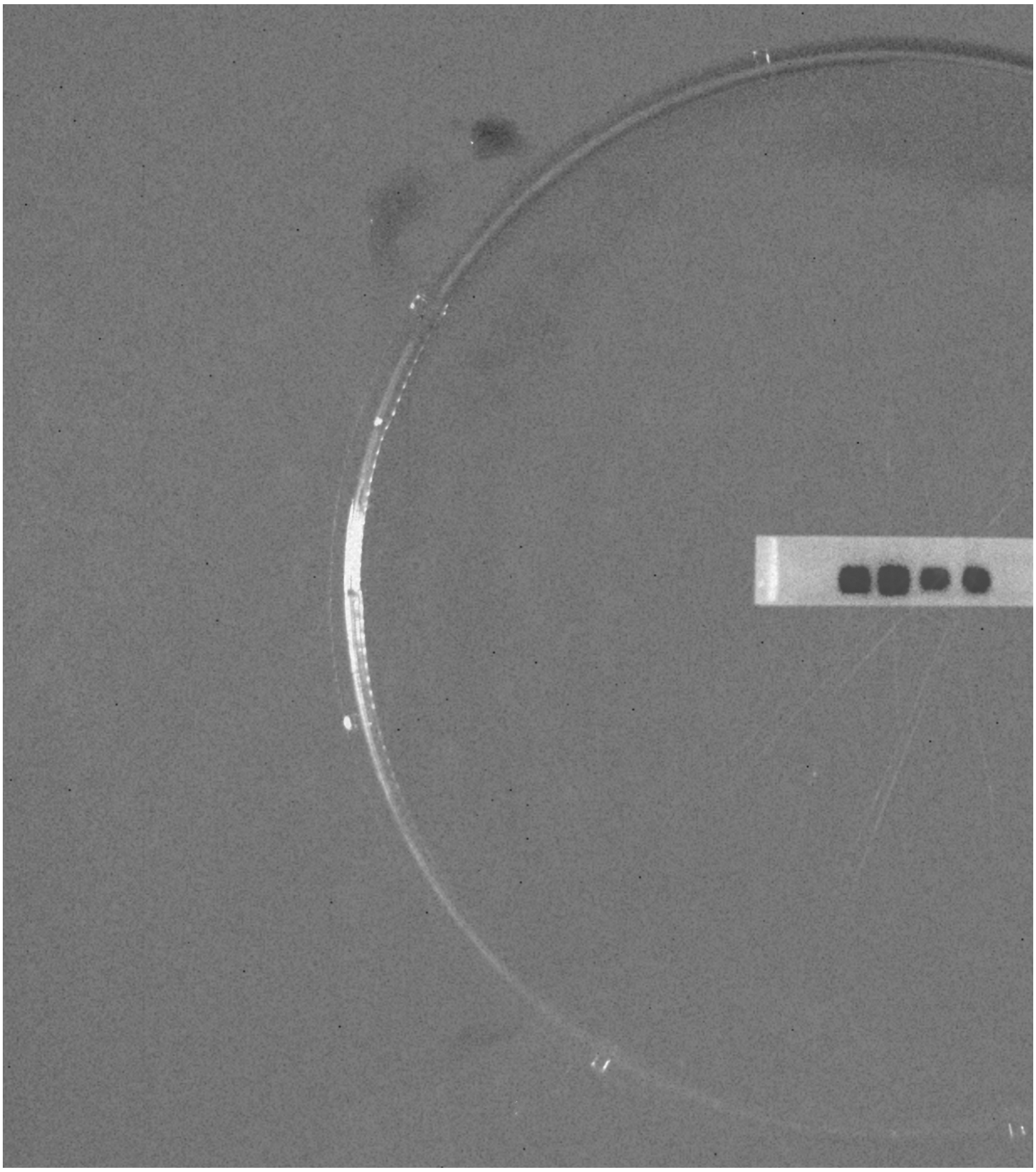

Fig7E-GAPDH-4

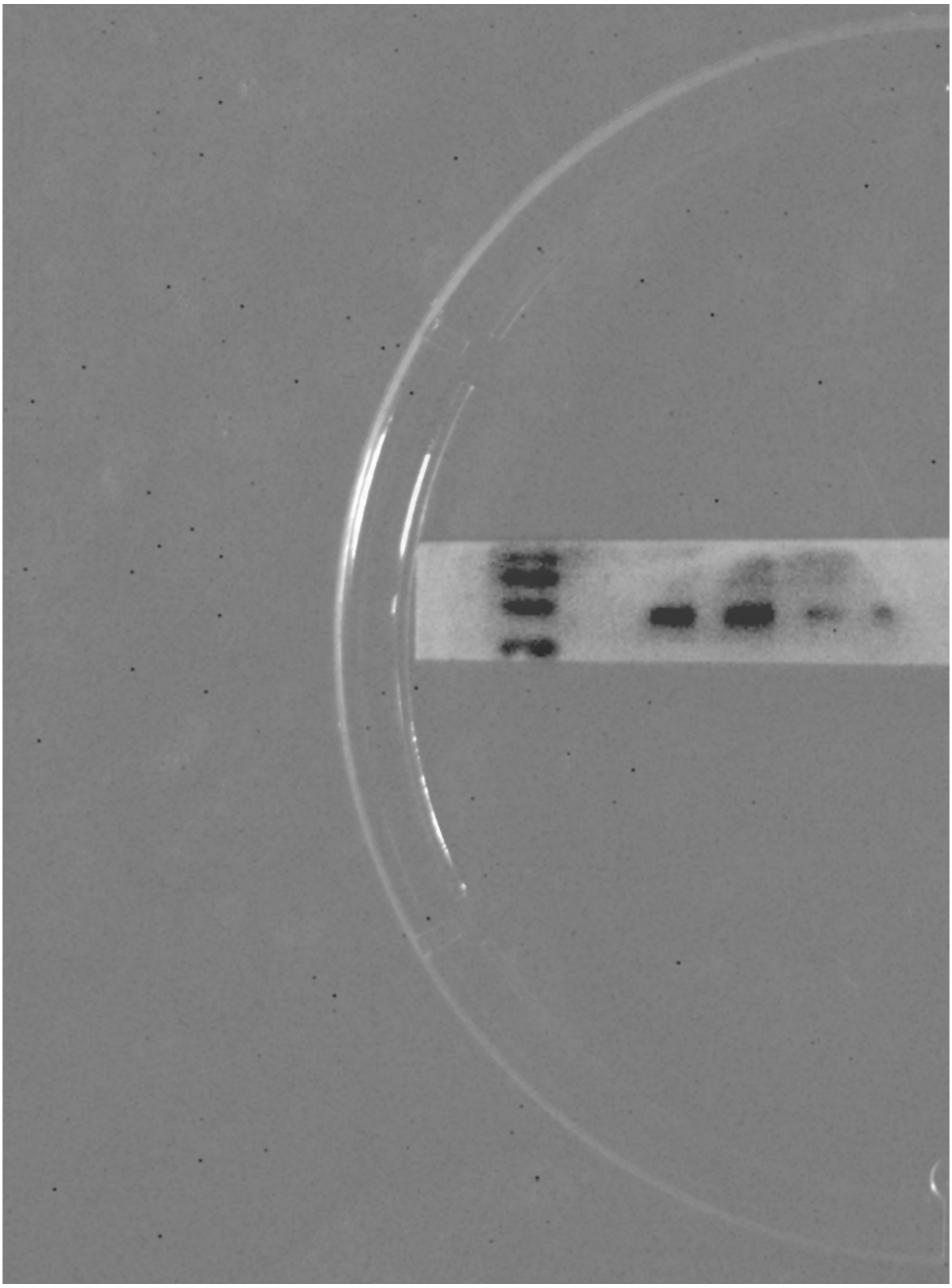

Fig7E-STING-4

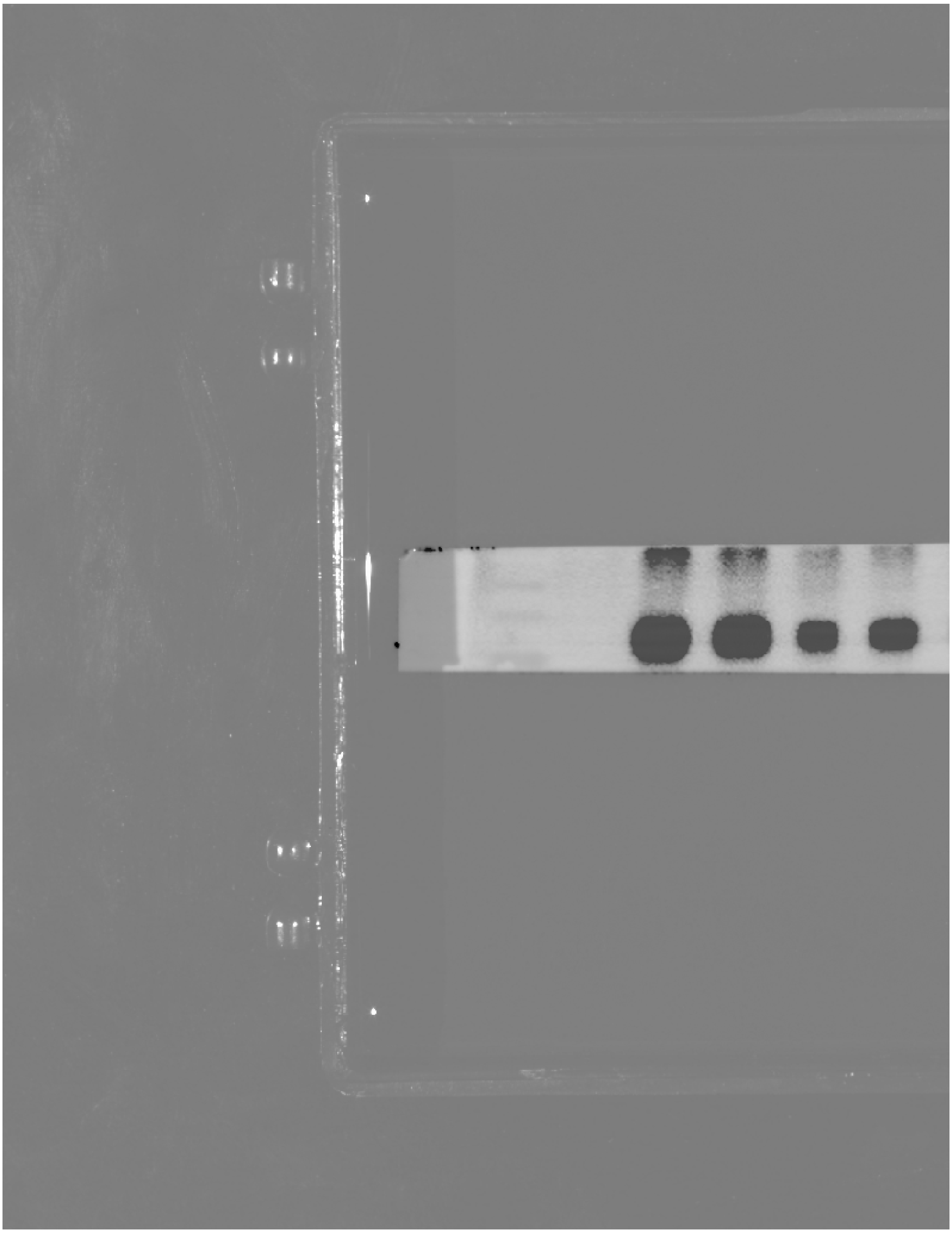

Fig7E-cGAS-4

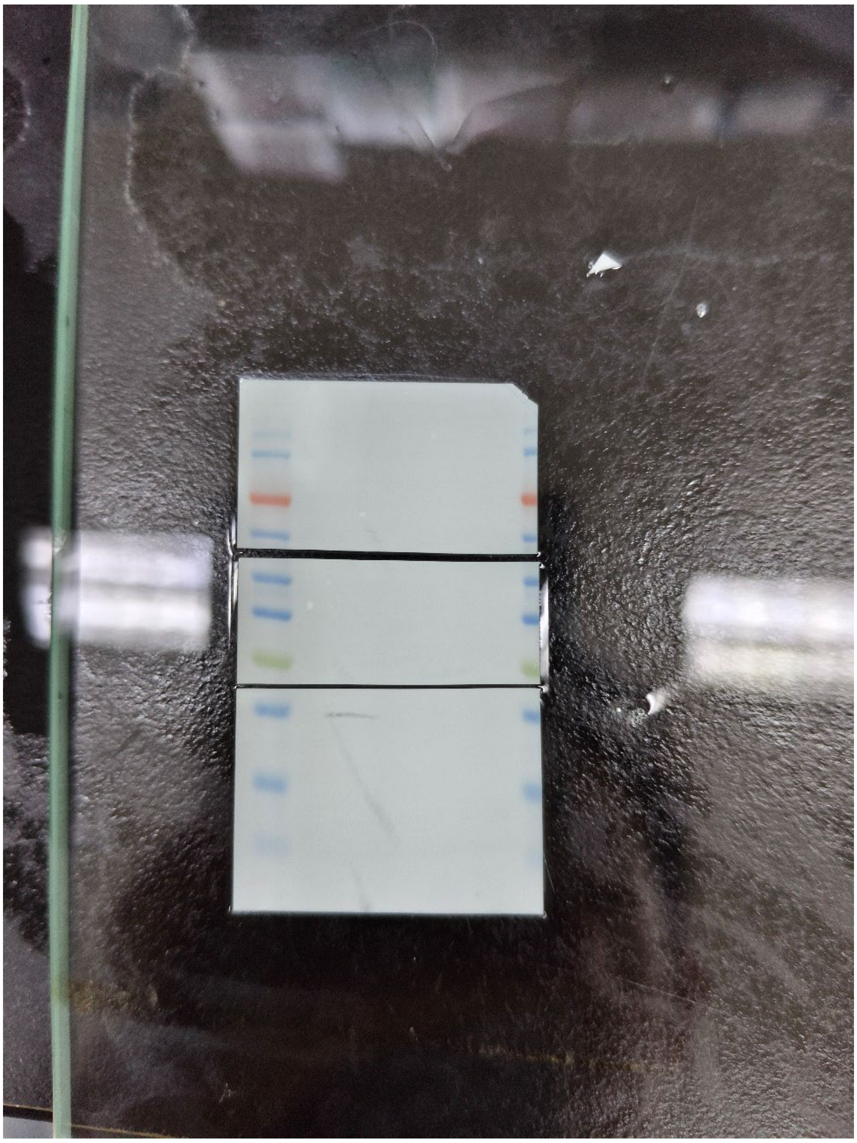

Fig7E-Tubin&STING-1-全膜

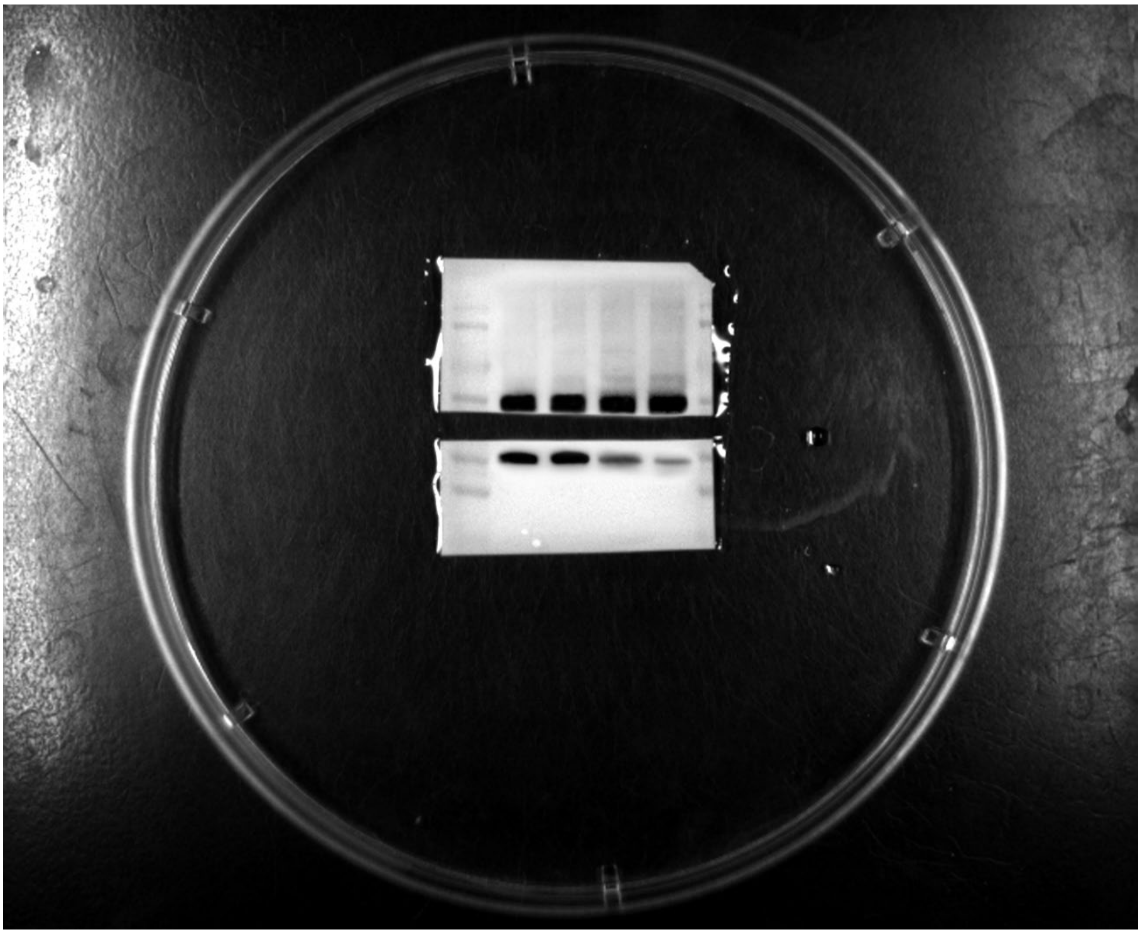

Fig7E-Tubin&STING-1

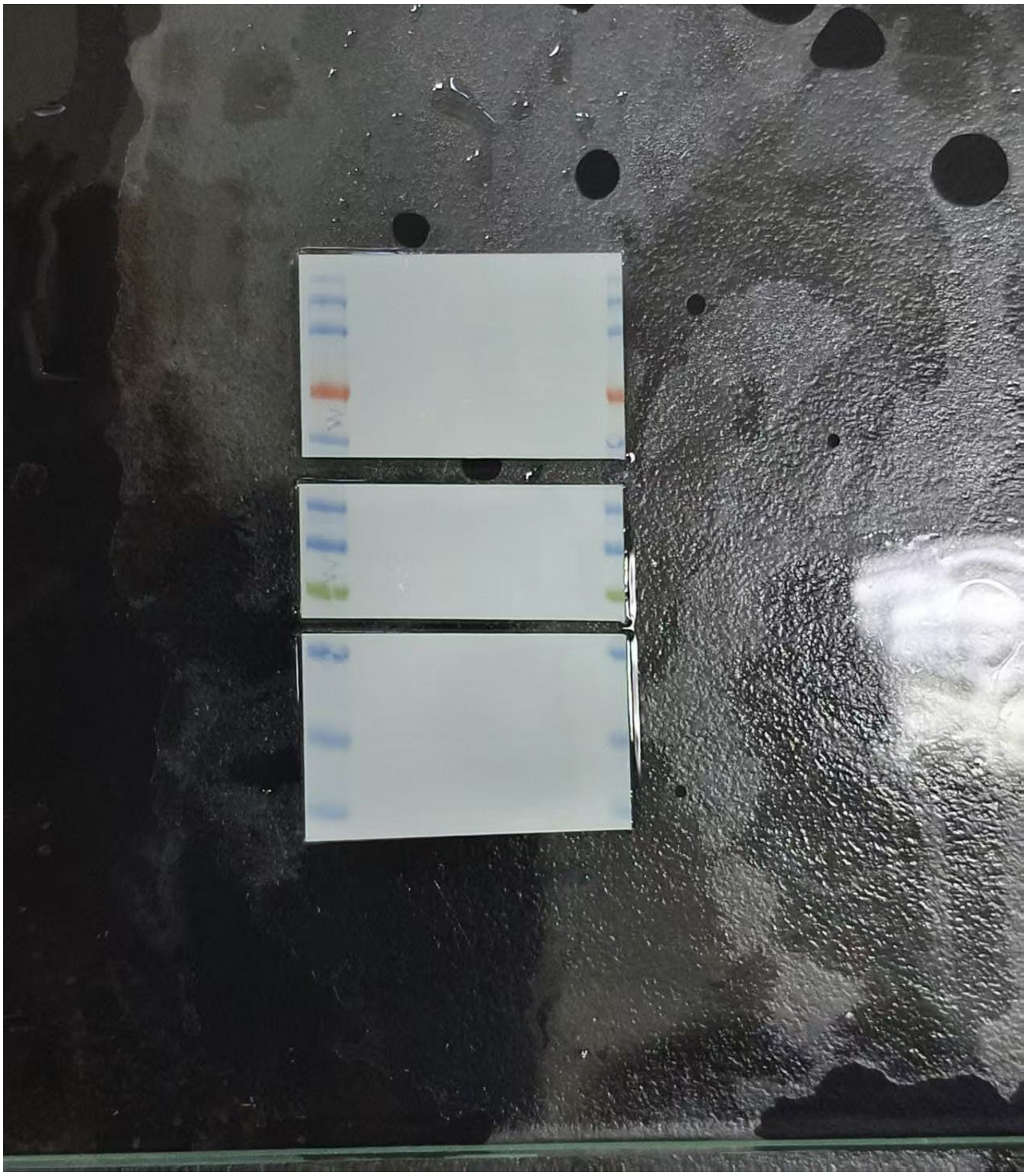

Fig7E-Tubin&STING-2-全膜

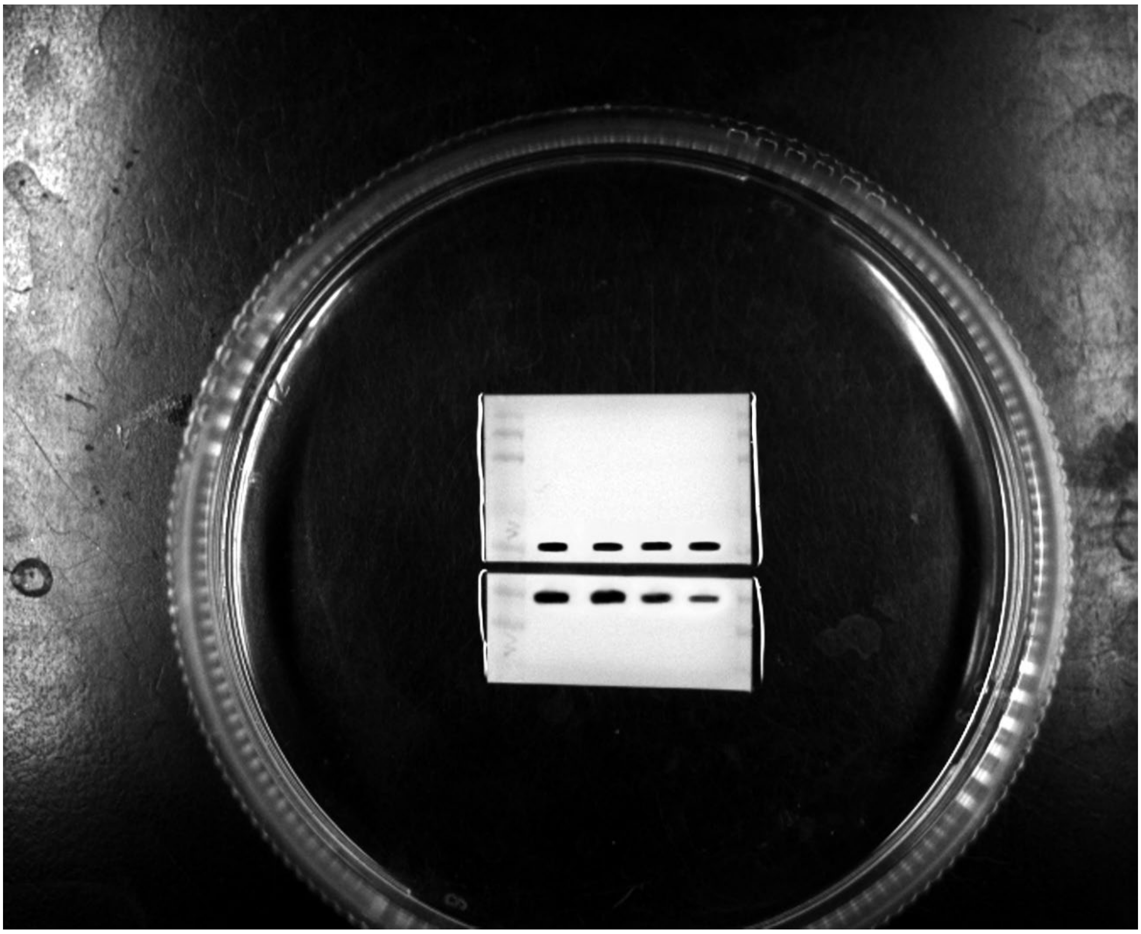

Fig7E-Tubin&STING-2

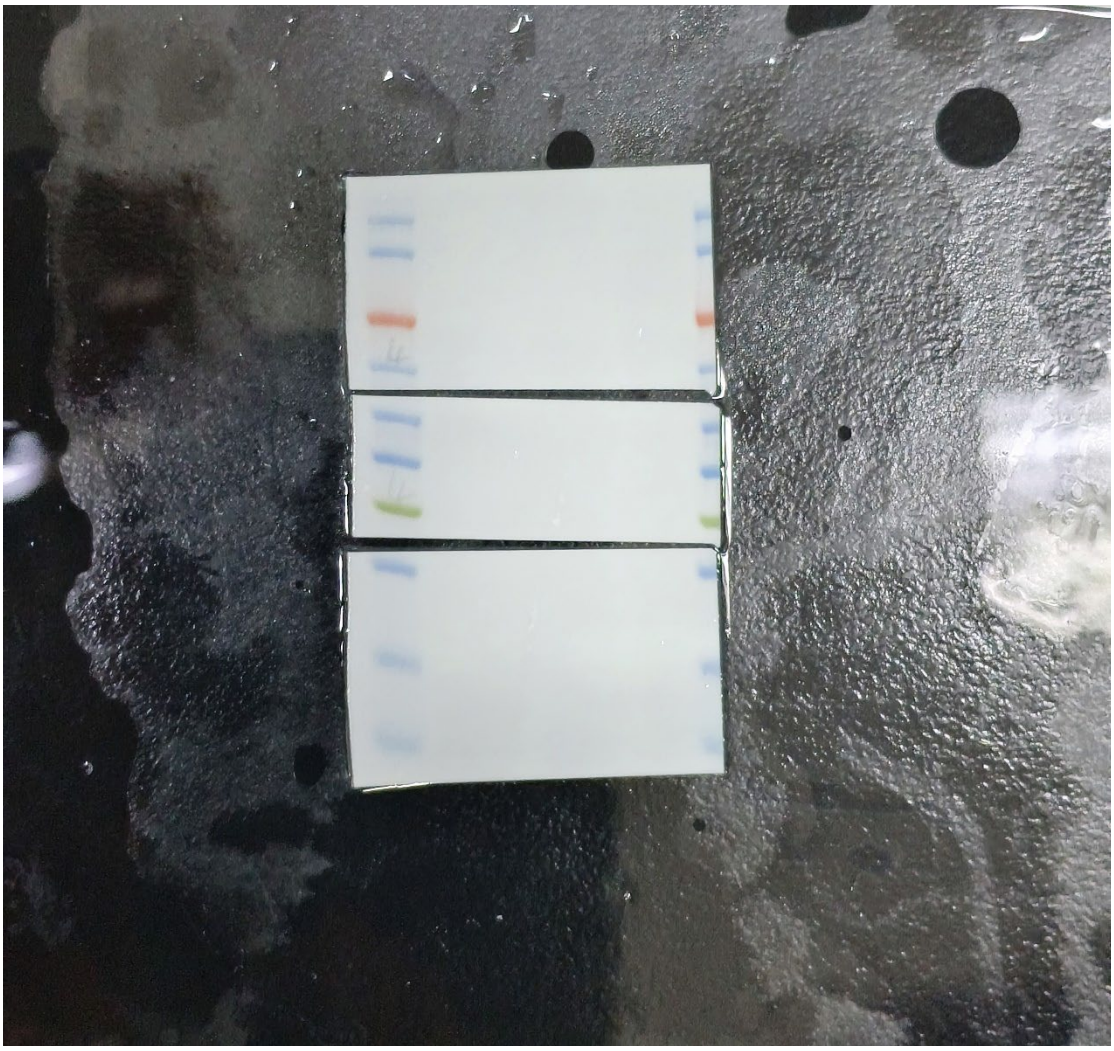

Fig7E-Tubin&STING-3-全膜

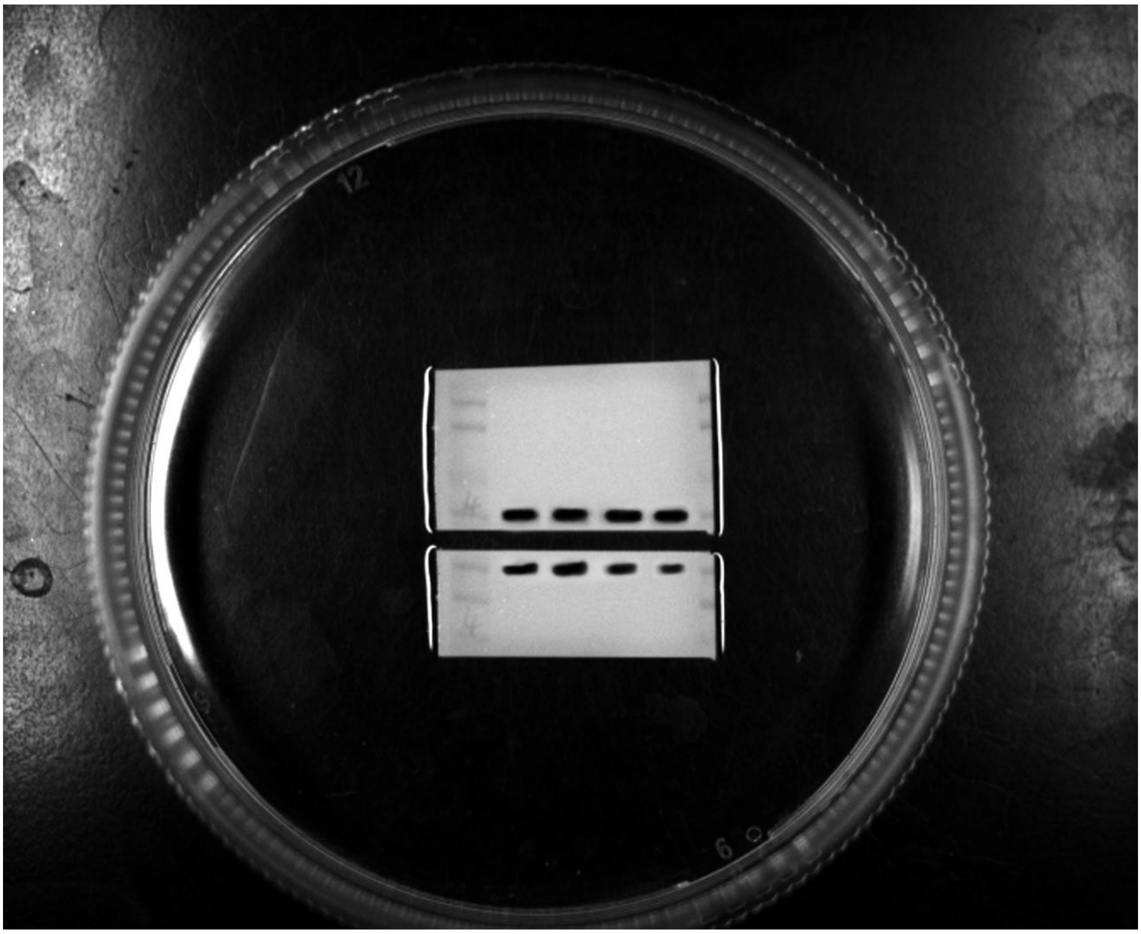

Fig7E-Tubin&STING-3

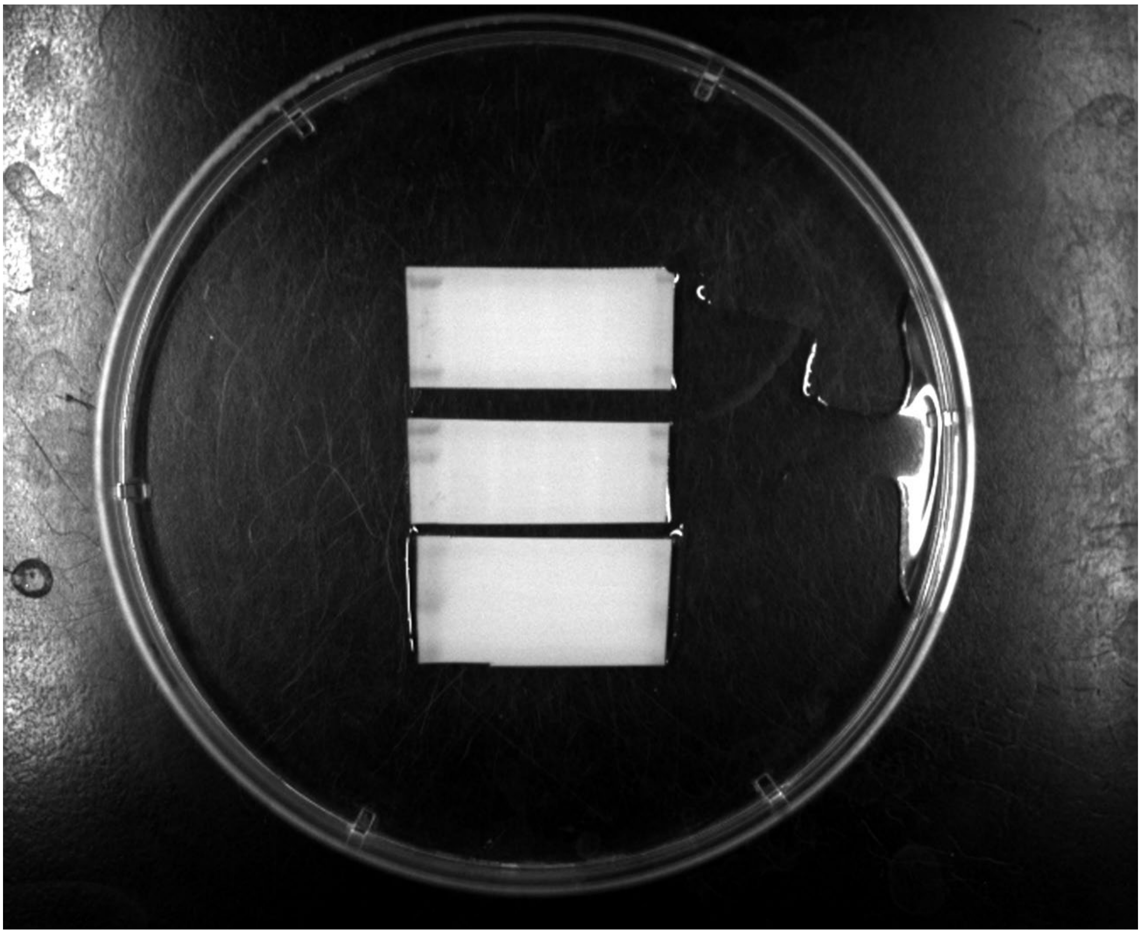

Fig7F-GAPDH&p62&LC3-1-全膜

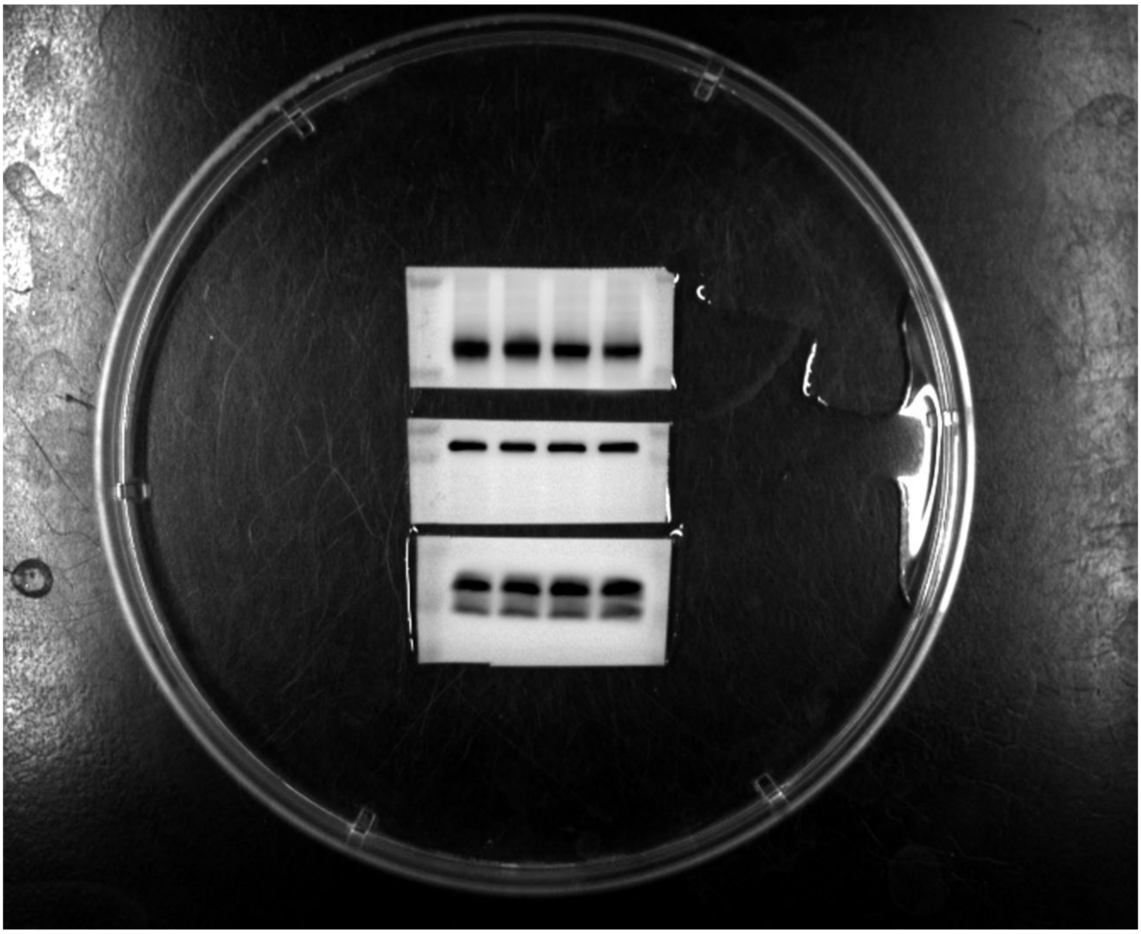

Fig7F-GAPDH&p62&LC3-
1-

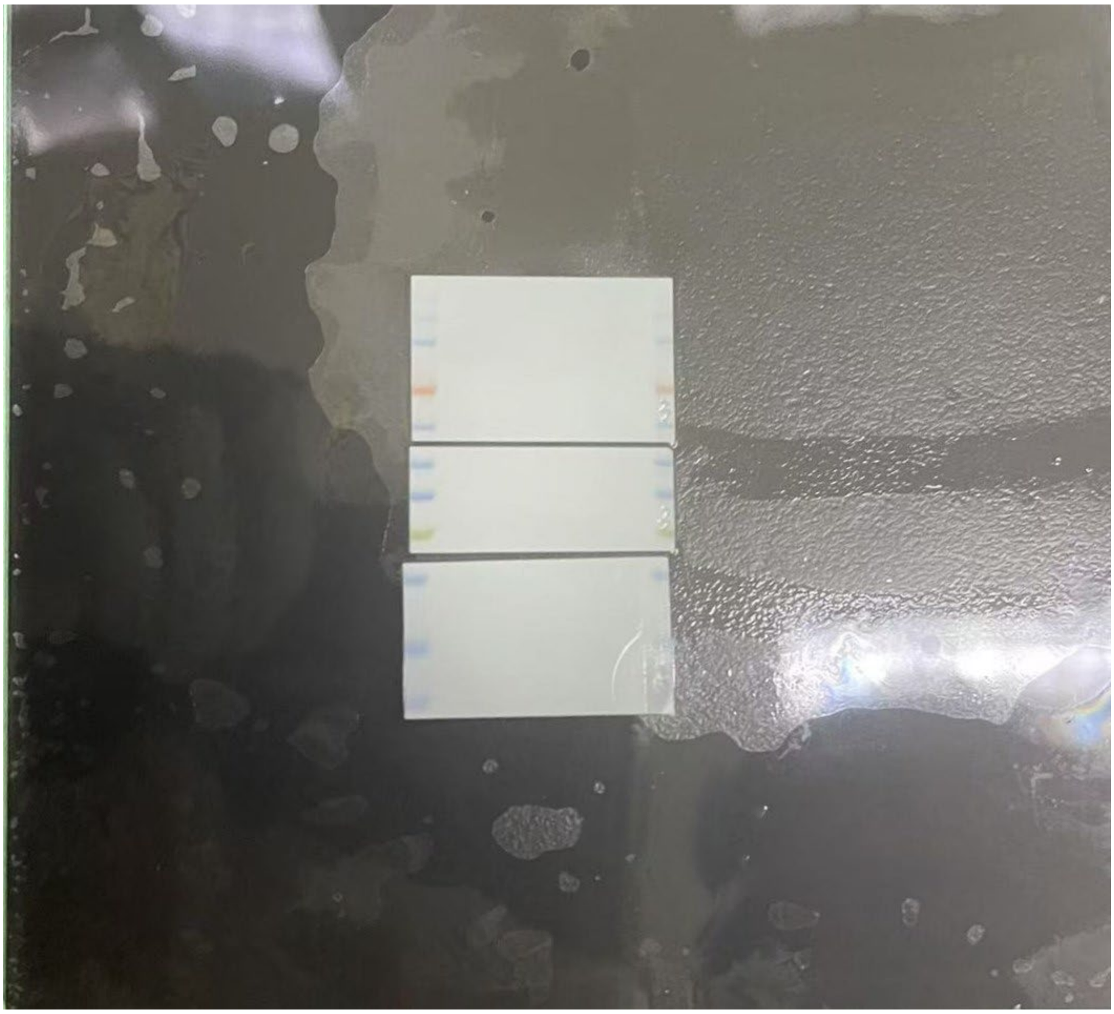

Fig7F-GAPDH&p62&LC3-2-全膜

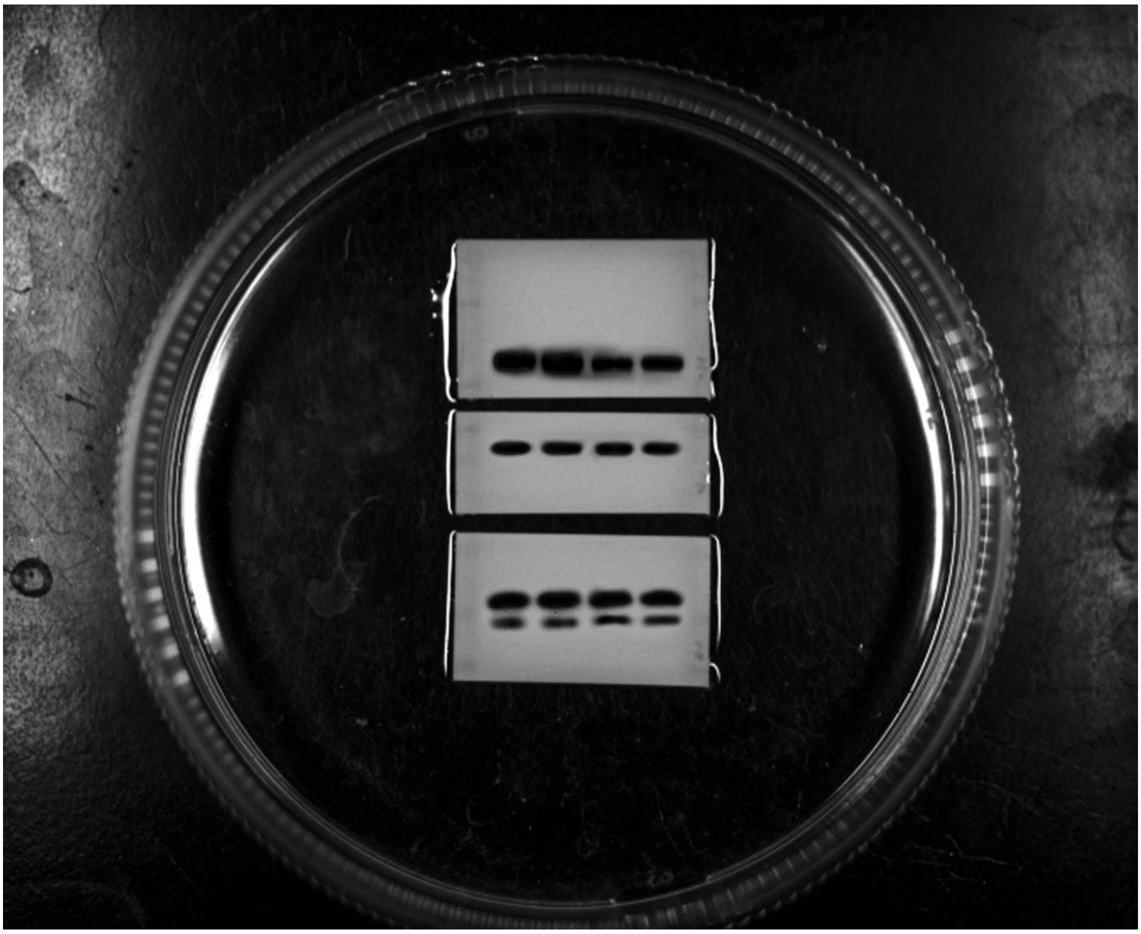

Fig7F-GAPDH&p62&LC3-2

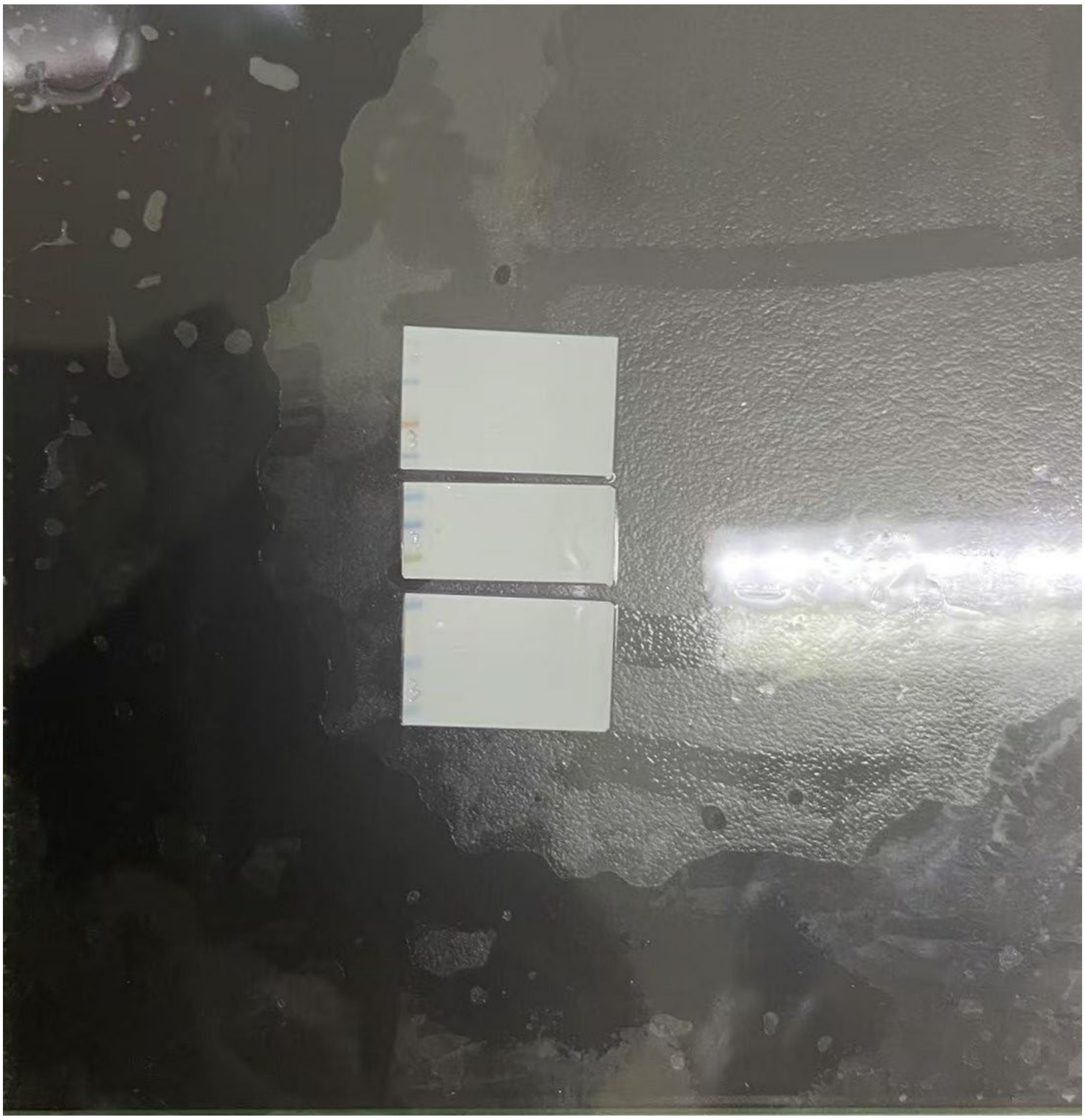

Fig7F-GAPDH&p62&LC3-3-全膜

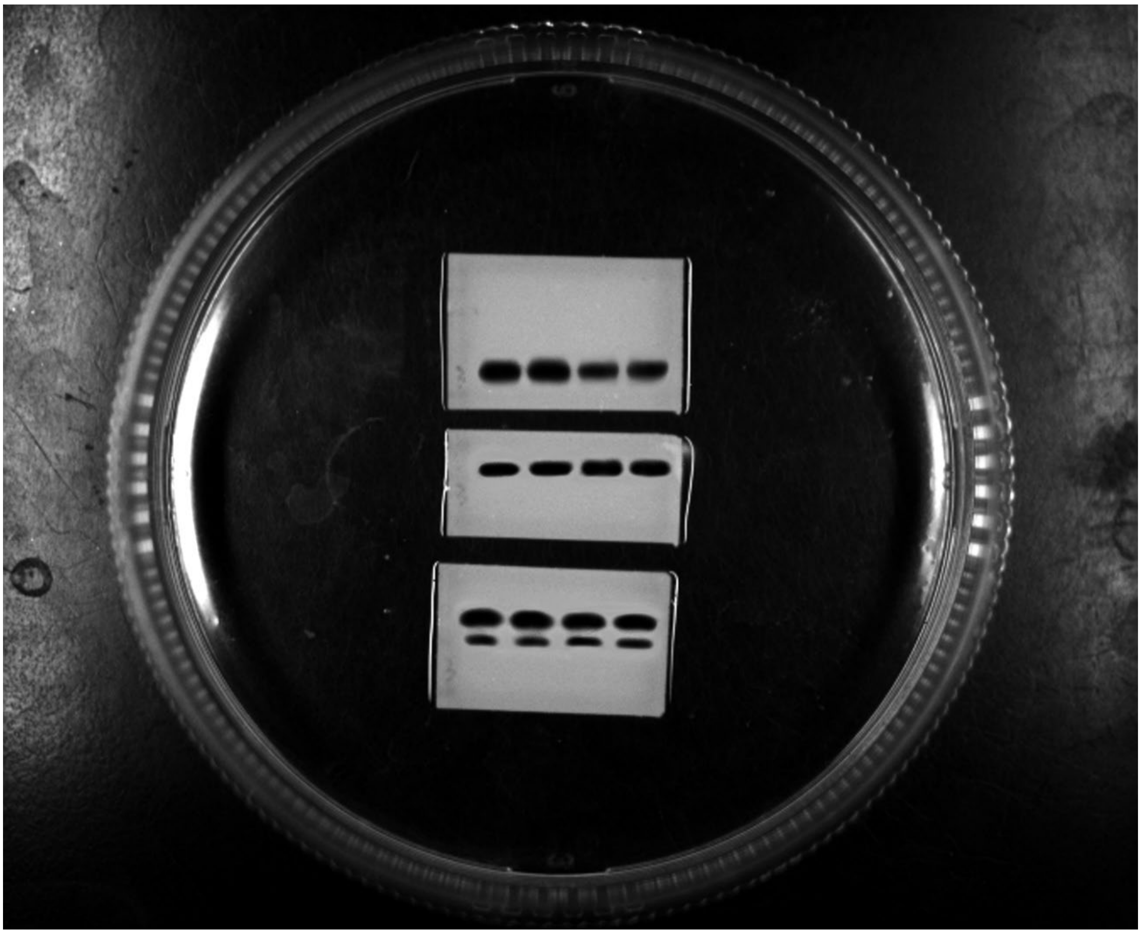

Fig7F-GAPDH&p62&LC3-3

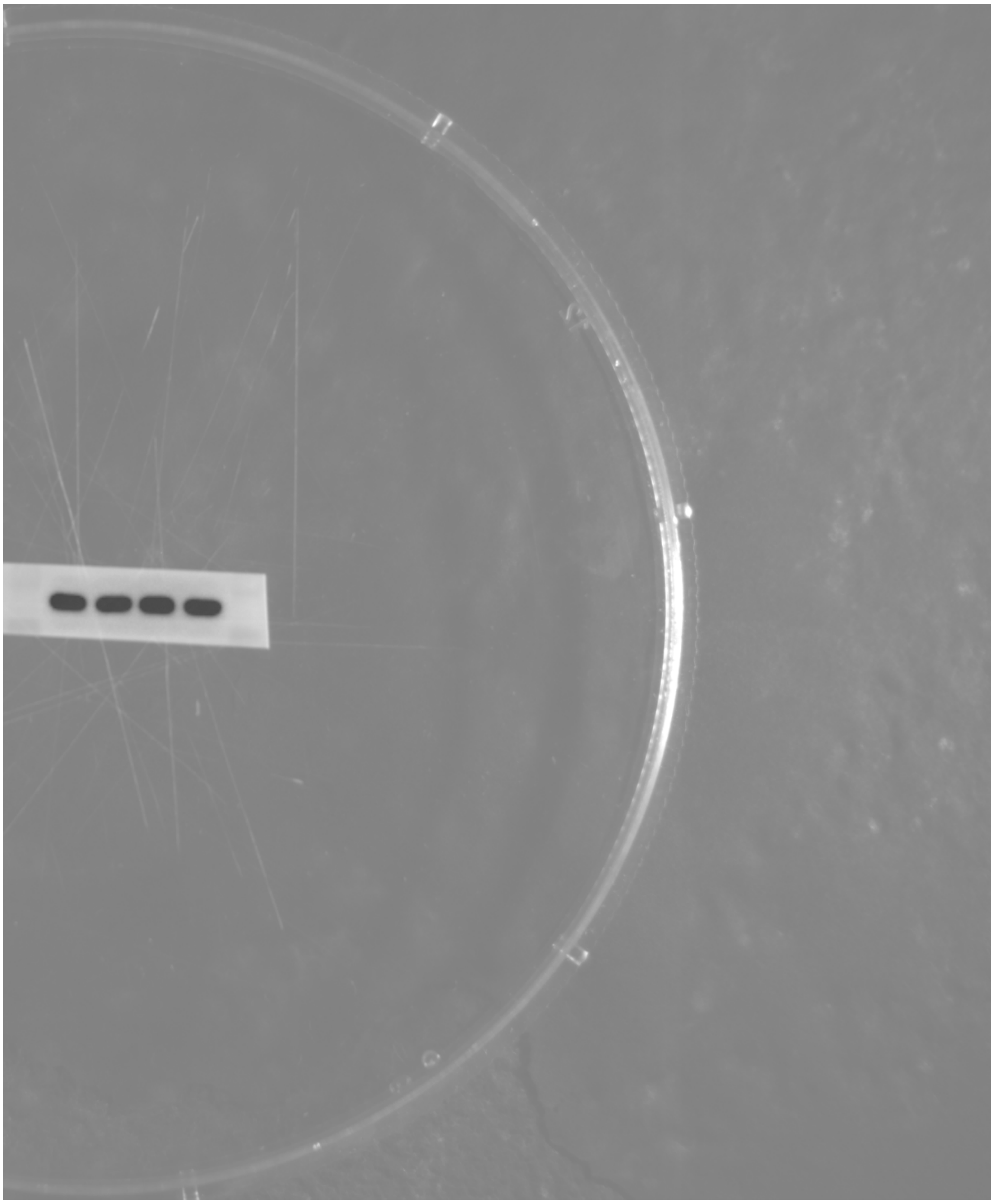

Fig7F-GAPDH-4

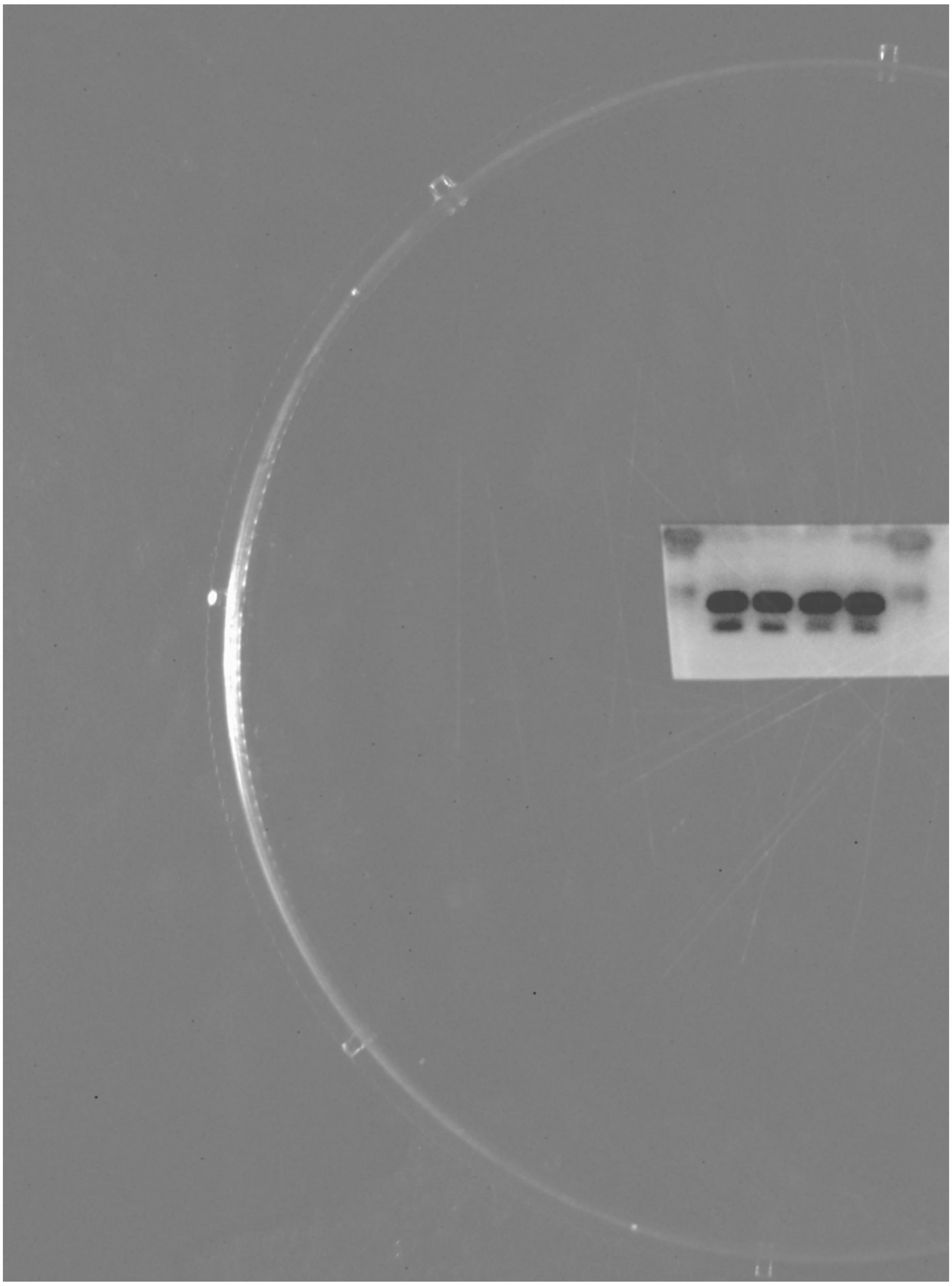

Fig7F-LC3-4

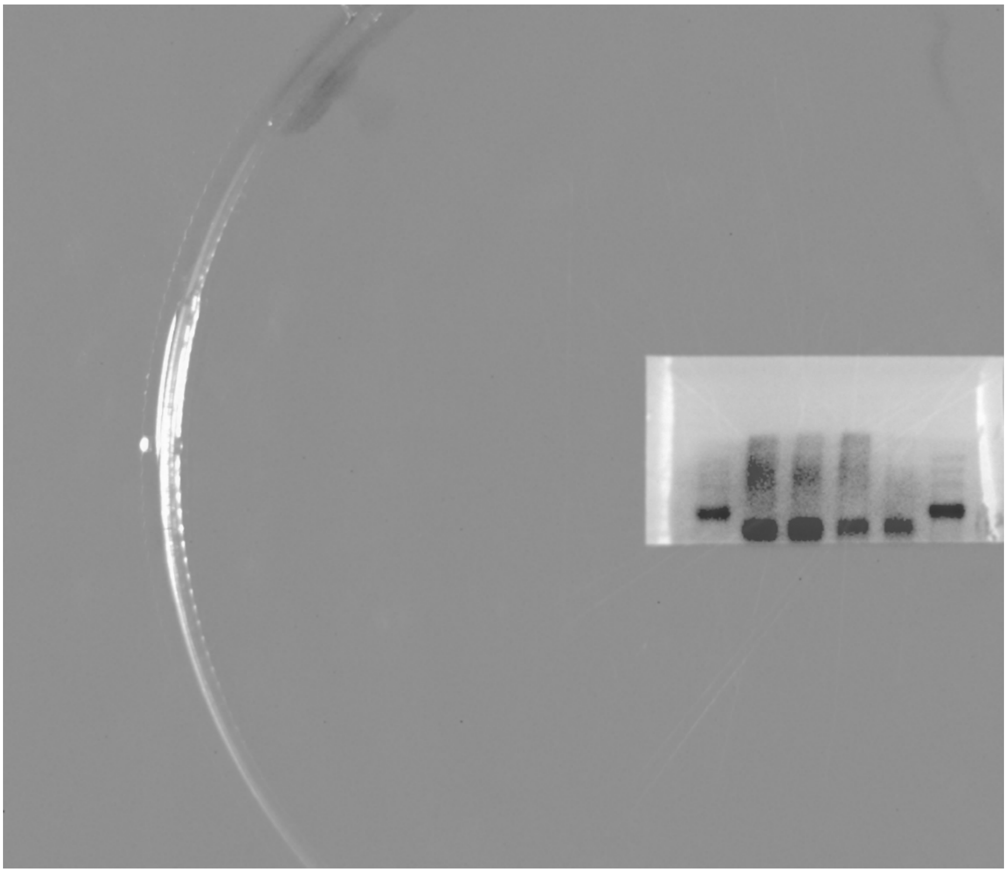

Fig7F-p62-4
